# Galantamine-Curcumin Hybrids as Dual-Site Binding Acetylcholinesterase Inhibitors

**DOI:** 10.3390/molecules25153341

**Published:** 2020-07-23

**Authors:** Georgi Stavrakov, Irena Philipova, Atanas Lukarski, Mariyana Atanasova, Dimitrina Zheleva, Zvetanka D. Zhivkova, Stefan Ivanov, Teodora Atanasova, Spiro Konstantinov, Irini Doytchinova

**Affiliations:** 1 Faculty of Pharmacy, Medical University of Sofia, 1000 Sofia, Bulgaria; alukarski@pharmfac.mu-sofia.bg (A.L.); matanasova@pharmfac.mu-sofia.bg (M.A.); dzheleva@pharmfac.mu-sofia.bg (D.Z.); zzhivkova@pharmfac.mu-sofia.bg (Z.D.Z.); ivanovs@umd.edu (S.I.); tatanasova@pharmfac.mu-sofia.bg (T.A.); skonstantinov@pharmfac.mu-sofia.bg (S.K.); 2 Institute of Organic Chemistry with Centre of Phytochemistry, Bulgarian Academy of Sciences, 1113 Sofia, Bulgaria; irena@orgchm.bas.bg; 3 Institute for Bioscience and Biotechnology Research, University of Maryland, Rockville, MD 20850, USA

**Keywords:** virtual screening, molecular docking, neurotoxicity, ADME, BBB permeability

## Abstract

Galantamine (GAL) and curcumin (CU) are alkaloids used to improve symptomatically neurodegenerative conditions like Alzheimer’s disease (AD). GAL acts mainly as an inhibitor of the enzyme acetylcholinesterase (AChE). CU binds to amyloid-beta (Aβ) oligomers and inhibits the formation of Aβ plaques. Here, we combine GAL core with CU fragments and design a combinatorial library of GAL-CU hybrids as dual-site binding AChE inhibitors. The designed hybrids are screened for optimal ADME properties and BBB permeability and docked on AChE. The 14 best performing compounds are synthesized and tested in vitro for neurotoxicity and anti-AChE activity. Five of them are less toxic than GAL and CU and show activities between 41 and 186 times higher than GAL.

## 1. Introduction

Most of the neurodegenerative diseases are associated with altered levels of the transmitter acetylcholine (ACh) in selected areas of the brain [1]. The enzyme acetylcholinesterase (AChE) catalyzes the hydrolysis of ACh in the cholinergic synapses. By inhibiting the AChE, the levels of ACh increase and the neurodegeneration improves symptomatically. Moreover, AChE interacts with the amyloid-β (Aβ) peptide throughout the peripheral anionic site (PAS) and forms highly neurotoxic AChE-Aβ complexes that promote the assembly of Aβ into amyloid fibrils [2]. The amyloid fibrils grow progressively and irreversibly to amyloid plaques—one of the main characteristics of Alzheimer’s disease (AD) [3]. Hypothetically, compounds binding simultaneously to the catalytic active site (CAS) and the PAS of AChE, are able both to prevent the ACh hydrolysis and the onset of amyloidogenesis. This hypothesis prompted the search for dual-site binding AChE inhibitors as multitarget agents for AD treatment [4,5,6,7,8,9,10,11,12,13,14,15,16,17,18]. Unfortunately, since 2003, no new drugs have been approved for the treatment of AD [19]. 

**Figure 1:**
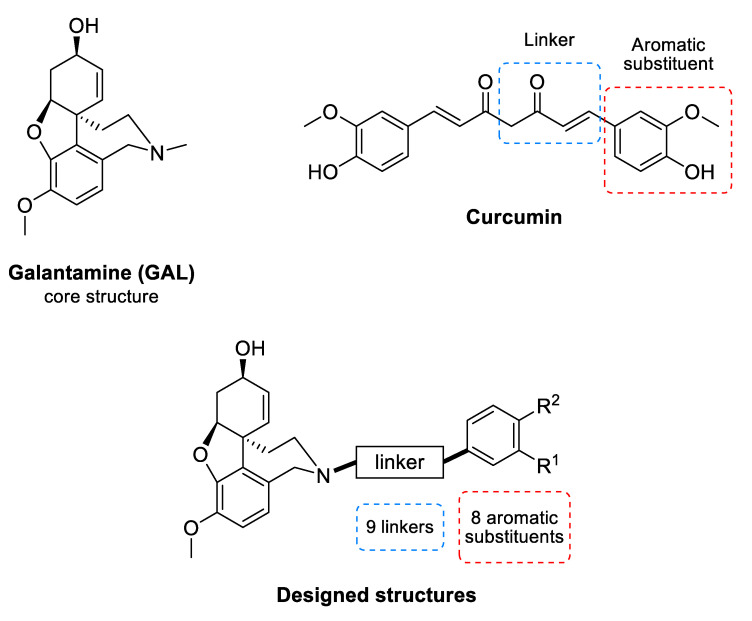
Common structure of the designed GAL-CU hybrid molecules.

Galantamine (GAL) (Figure 1) is an alkaloid isolated from the bulbs and flowers of *Leucojum aestivum* L. and *Galanthus* sp. It is an inhibitor of AChE [20] and is among the few drugs approved for the treatment of AD. In addition, GAL has been identified as an allosteric nicotinic acetylcholine receptor (nAChR) modulator [21]. The stimulation of nAChRs increases the intracellular Ca^2+^ levels and facilitates the release of noradrenaline. Both effects improve the cognitive function of the brain [21]. GAL treatment of rat microglia significantly increases the phagocytosis of amyloid β (Aβ) peptide and facilitates the clearance of Aβ in the brain of rodents with AD [22]. This multitarget action of GAL makes it a valuable drug for AD treatment and stimulates the search for new GAL derivatives with higher affinity to AChE [23,24,25,26,27,28,29,30,31].

Curcumin (CU) (Figure 1) is a natural polyphenolic compound isolated from the rhizomes of *Curcuma longa* L. It has a symmetrical molecular structure and its IUPAC name is (1E,6E)-1,7-Bis(4-hydroxy-3-methoxyphenyl)hepta-1,6-diene-3,5-dione. There are two more curcuminoids—demethoxycurcumin and bisdemethoxycurcumin. All three compounds are biologically active. The main features of the curcuminoid family are the following structural motifs: a benzene ring conjugated with α-β unsaturated carbonyl moiety, hydroxyl groups on the fourth position in the benzene ring, and methoxy group present in two of the three members at third position. Usually, CU exists as two tautomeric forms as one of the carbonyl groups transforms into an enolic group. CU binds to Aβ oligomers and fibrils and inhibits the β-sheet formation [32]. It was found that the aromatic methoxy and/or hydroxy groups interact with the V12 and 16KLVFFA21 residues of the Aβ [33,34], while the aryl rings make π-π stacking with the aromatic residues [32]. The enone group and the unsaturated carbon spacer are also essential for the anti-Aβ aggregation activity [33,34,35]. 

In the present study, we designed a set of GAL-CU hybrids as dual-site binding AChE inhibitors with potentially anti-amyloid aggregation activity. The hybrids underwent two-step virtual screening. First, they were screened for optimal ADME properties and blood-brain barrier (BBB) permeability and then, a docking-based virtual screening on human AChE was performed. The highest scored hybrids were synthesized and tested for cytotoxicity and AChE activity. Five of them were more active and less toxic than GAL and CU themselves. 

## 2. Results

### 2.1. Design of GAL-CU Hybrids 

**Figure 2:**
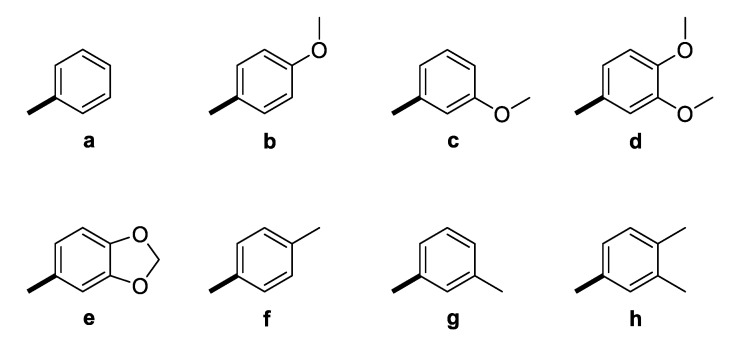
Aromatic substituents (Ar) used in the designed GAL-CU hybrid molecules.

The GAL-CU hybrid structures were composed of a core, a linker, and an aromatic group. The GAL molecule was used as a core. The linkers started from GAL amine’s methyl group and ended with aromatic moieties mimicking the CU structure (Figure 1). Eight aromatic substituents (assigned by the letters **a**–**h**) were designed to mimic the CU phenyl rings (Figure 2). The first substituent **a** consisted of a single phenyl ring. The substituents **b**–**d** contained a phenyl ring with methoxy groups at *m*- or *p*-position, or both. The substituent **e** was similar to **d**, but the two methoxy groups were replaced by a more constrained 1,3-dioxole ring. The remaining substituents **f**–**h** contained phenyl rings with bioisosteric methyl groups at *m*- or *p*-position, or both.

**Figure 3:**
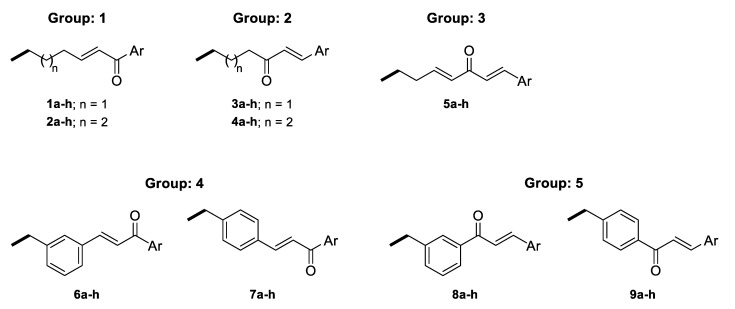
Linkers used in the designed GAL-CU hybrid molecules.

The linkers were designed to resemble the CU α-β unsaturated fragment. In our previous studies on GAL derivatives [36,37,38], it was found that a linker containing five to seven carbon atoms is optimal for dual-site binding to AChE. Seven types of linkers were suggested for the present GAL-CU hybrids and the designed compounds were divided into five groups according to the linker type (Figure 3). In the first two groups of linkers, the place of a double bond according to a carbonyl group was varied, as well as the linkers’ lengths. The third linker resembles the tautomeric form of CU, with one double bond on each side of the carbonyl group. The last two groups of linkers incorporate a benzene ring in the middle because of the possible π-π interaction with Tyr341 and Tyr337 in *h*AChE [20]. Both *m*- and *p*-isomers were considered. In total, 72 GAL-CU hybrids were designed in the present study.

### 2.2. ADME Filters 

The 72 newly designed GAL-CU hybrids were tested for BBB permeability, gastrointestinal (GI) absorption and non-specific interactions with numerous biological targets (PAINS filter), as described in Section 4. Only 44 compounds passed all criteria: 12 from group 1, 15 from group 2, 8 from group 3, 3 from group 4, and 6 from group 5.

### 2.3. Virtual Screening by Molecular Docking

The 44 molecules that passed the ADME filters were docked to human rAChE, as described in the Materials and methods section. Only docking poses with root mean square difference (RMSD) < 1.5 Å of the referent GAL structure were considered. The GoldScores of the newly designed compounds ranged from 114.54 to 88.28, and all were higher than the GoldScore of GAL (78.25) (Table 1). Among the top 33% of the best-scored compounds (GoldScore range 114.54–104.00) were compounds with aromatic substituents **a**, **b**, **c**, **f**, **g** and **h** and linkers from group 4 (series **6**) and group 5 (series **8**).

### 2.4. GAL-CU Hybrids Selected for Synthesis

The GAL-CU hybrids selected for synthesis are given in bold in Table 1. Among the top 33% of the best-scored compounds were selected from series **6** and **8**. Compounds **6a**, **6b**, **8b**, **8c**, **8f**, **8g**, and **8h** from the top range were selected for synthesis. In our previous studies, good correlations between the GoldScores and the experimental IC_50_ values to AChE of GAL derivatives were observed [36,37,38,39]. Here, in order to examine this relationship, several compounds from the middle (103.99–94.00) and low GoldScore range (93.99–84.00) were selected for synthesis as well. Compounds **4b**, **4e**, **4f** and **4h** were selected from the middle range, while compounds **4a**, **4c** and **4g** were selected from the low range of GoldScore. In total, 14 GAL-CU hybrids were selected for synthesis.

### 2.5. Synthesis of Selected GAL-CU Hybrids

#### 2.5.1. Synthesis of Hybrids 4a–c,e–h 

**Figure 4:**
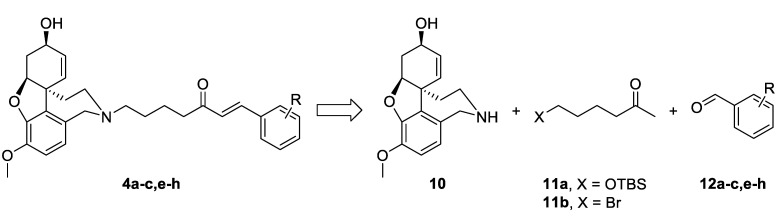
Retrosynthetic analysis of compounds **4a**–**c**,**e**–**h**.

The synthetic strategy used in the present study relied on the construction of linkers binding different aromatic moieties to Norgalantamine (NOR) **10** (Figure 4). Ketones of the type **11** were foreseen as key intermediates, due to possible Aldol condensation with chosen benzaldehydes **12a**–**c**,**e**–**h**. Further on, we envisaged the introduction of a leaving group on the aliphatic end of the linker, because of the final nucleophilic substitution with NOR **10** towards the desired target compounds **4a**–**c**,**e**–**h** (Figure 4).

**Scheme 1:**
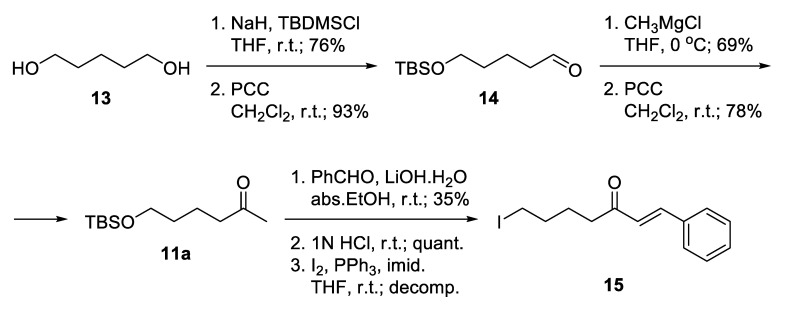
Attempted synthesis of iodo-linkers.

Notably, 1,5-Pentandiol **13** was considered as an available and inexpensive starting material (Scheme 1). It allows independent functionalization of its two hydroxyl groups. Monoprotection as *tert*-butyldimethylsilyl ether and subsequent mild oxidation proceeded with good yields to give the unstable aldehyde **14 [40]**. The latter was immediately subjected to Grignard attack to give a racemic secondary alcohol, which was oxidized to ketone **11a [41]**. Aldol condensation of the ketone with benzaldehyde was accomplished using catalytic amounts of lithium hydroxide monohydrate as a base in absolute ethanol. The product was isolated in low yield. Thus, the hydrocarbon skeleton of the desired linker was generated. The deprotection of the silyl ether proceeded smoothly in slightly acidic media, and the free alcohol was transformed into iodide **15**, which unfortunately decomposed during the reaction. Obviously, the iodide was not the proper choice for a leaving group because of its high reactivity.

**Scheme 2:**
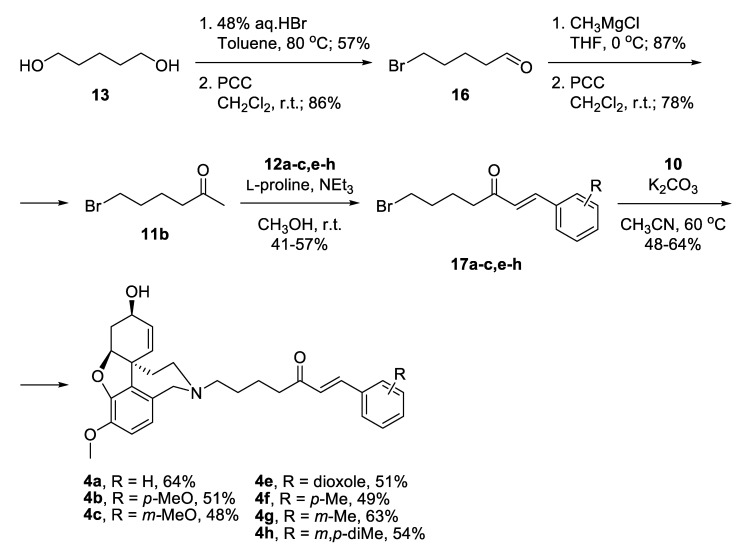
Synthesis of compounds **4a**–**c**,**e**–**h** via bromo-linkers.

Alternatively, a monobromination of 1,5-pentandiol **13** proceeded with excellent selectivity because the product is water-insoluble and leaves the aqueous phase where the reaction takes place (Scheme 2) [42]. Mild oxidation afforded 5-bromopentanal **16 [43]**, which was attacked by CH_3_MgCl and subsequently oxidized to give 6-bromohexan-2-one **11b**. A key feature for the success of this shorter route was the application of Aldol condensation under mild reaction conditions. The desired bromo-linkers **17a**–**c**,**e**–**h** were synthesized after days stirring of the bromo-ketone **11b** with chosen benzaldehyde **12a**–**c**,**e**–**h** and catalytic amounts of L-proline, and triethylamine in methanol media [44]. The products of condensation were isolated in moderate yields. Nucleophilic substitution of the bromo-linkers with NOR **10** in the presence of K_2_CO_3_ as a base afforded the desired target compounds **4a**–**c**,**e**–**h** in 48% to 64% yield (Scheme 2).

#### 2.5.2. Synthesis of Hybrids 6a and 6b

**Figure 5:**
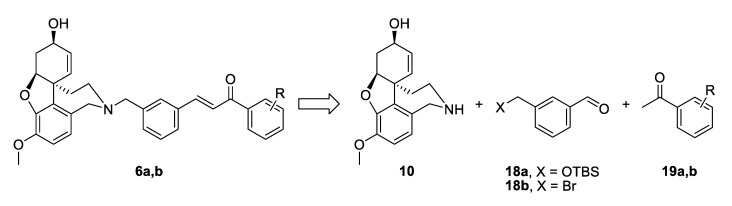
Retrosynthetic analysis of compounds **6a**,**b**.

The synthetic strategy towards compounds **6a** and **6b** again included two key steps: Aldol condensation for the linker construction and nucleophilic substitution with NOR **10** at a later stage (Figure 5). The difference compared to compounds type **4** was the change of places of aldehyde and ketone, due to the change of positions of carbonyl group and double bond in the conjugated enone system. Thus, we planned the synthesis of benzaldehydes of type **18**, subsequent condensation with chosen acetophenones **19a**,**b** and a final attachment to the alkaloid core (Figure 5). 

**Scheme 3:**
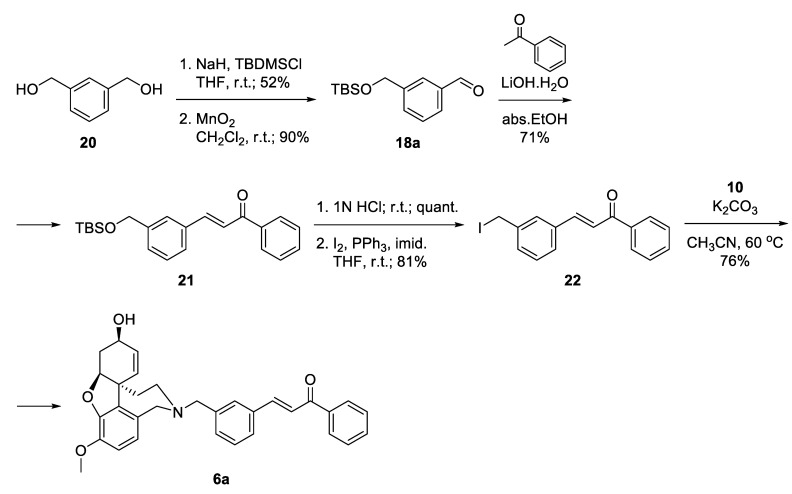
Synthesis of **6a** via an iodo-linker.

The synthesis of **6a** started with the monoprotection of 1,3-phenylenedimethanol as *tert*-butyldimethylsilyl ether and its oxidation with manganese(IV) oxide to give benzaldehyde **18a [45]**. Aldol condensation of the latter with acetophenone in the presence of 20 mol% LiOH. H_2_O in ethanol afforded ketone **21 [46]**, which was deprotected quantitatively and the resulting alcohol was converted into iodide **22** applying Appel reaction. The iodo-linker was attached to NOR **10** to give the desired product **6a** in 76% yield, after 6 steps and 20% overall yield (Scheme 3).

**Scheme 4:**
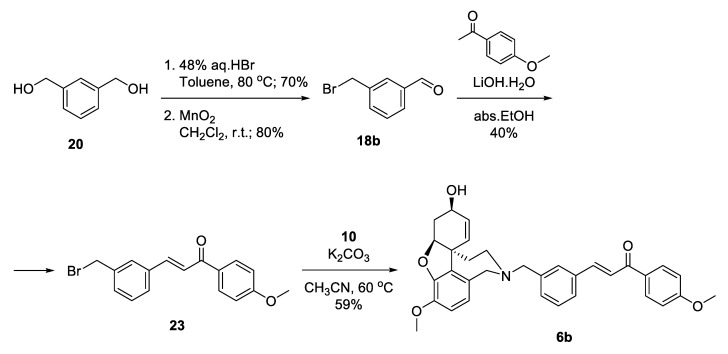
Synthesis of **6b** via a bromo-linker.

Aiming for optimization of the synthesis of **6b**, we decided to skip the silyl protection by introducing the leaving group into the very first step (Scheme 4). Monobromination of 1,3-phenylenedimethanol **20** proceeded selectively with good yield, and the product was oxidized to give benzaldehyde **18b**. Aldol condensation of the latter with acetanisole proceeded with catalytic ammounts of LiOH.H_2_O in ethanol to give **23** in moderate yield. Substitution of the bromo-linker with NOR resulted in the desired product **6b** in 59% yield, after 4 steps and 13% overall yield (Scheme 4). Although the synthetic pathway to **6b** was 2 steps shorter in comparison with **6a**, the overall yield was lower. When applying Aldol condensations in the presence of benzyl bromides, a concurrent nucleophilic substitution poisons the base. 

#### 2.5.3. Synthesis of Hybrids 8b,c,f,g,h

**Figure 6:**
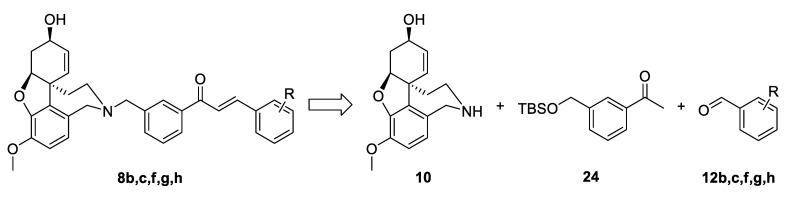
Retrosynthetic analysis of compounds **8b**,**c**,**f**,**g**,**h**.

For the synthesis of the target structures **8b**,**c**,**f**,**g**,**h**, we implemented the strategy developed for compounds of the type **4**. We planned the synthesis of acetophenone **24**, its condensation with chosen benzaldehydes **12b**,**c**,**f**,**g**,**h**, and finally the attachment of the constructed linkers to the galantamine core (Figure 6). 

**Scheme 5:**
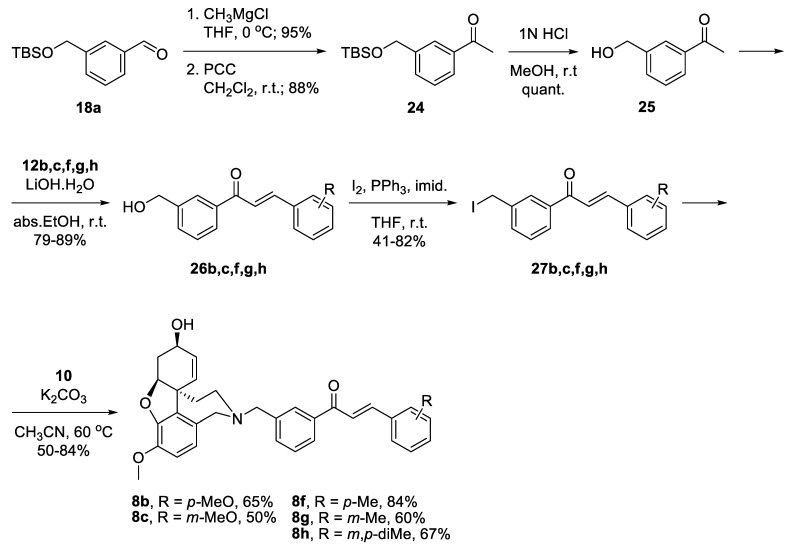
Synthesis of **8b**,**c**,**f**,**g**,**h**.

Benzaldehyde **18a** was converted into 3-(hydroxymethyl)acetophenone **25** via reaction with CH_3_MgCl, oxidation of the resulting alcohol to **24** and deprotection. Aldol condensation proceeded in good yields with the chosen benzaldehydes **12b**,**c**,**f**,**g**,**h** [47]. The resulting alcohols **26b**,**c**,**f**,**g**,**h** were converted into the corresponding iodides **27b**,**c**,**f**,**g**,**h**. Reactions of the latter with NOR **10** gave the desired target compounds **8b**,**c**,**f**,**g**,**h** (Scheme 5). 

The detailed synthetic procedures, analytical data and copies of ^1^H and ^13^C NMR spectra for the target compounds are given in a Supplementary File.

### 2.6. Neurotoxicity of the GAL-CU Hybrids

The neurotoxicity of the GAL-CU hybrids was tested on Neuro-2A cells, as described in Materials and methods. The IC_50_ values were in the range from 7.91 to 52.53 µM (Table 1). The IC_50_s for GAL and CU were >50 and 26.30 µM, respectively. Only five of the hybrids were less toxic than CU. One of them, compound **8b**, is among the best-scored compounds; two of them, compounds **4e** and **4f**, are from the middle GoldScore range; and two of them, compounds **4a** and **4b**, belong to the low GoldScore range.

### 2.7. Anti-AChE Activity

The anti-AChE activity of the GAL-CU hybrids, less toxic than GAL and CU, was measured in vitro by Ellman’s method, as described in Materials and Methods. As the docking studies were performed on *rh*AChE, while for the in vitro measurements was used AChE from electric eel (*ee*AChE), it was reasonably to question their relevance. The UniProt alignment of *rh*AChE (UniProt: P22303) and eeAChE (UniProt: O42275) have shown that all 17 residues forming the binding gorges are identical [36]. Additionally, our previous experience has shown that the docking scores predicted for *rh*AChE correlate well with the experimental binding affinity measured on *ee*AChE [36,37,38].

The IC_50_ values of the tested compounds are given in Table 1. For comparison, the IC_50_ values of GAL and CU as single molecules are given. GAL is a medium AChE inhibitor with IC_50_ = 3.52 µM, while CU is less active with IC_50_ = 68 µM. However, GAL-CU hybrids are more active than both of them. Compound **4b** is the most active with *IC_50_* = 0.02 µM, being 186 times more active than GAL. The compounds **4a**, **4e** and **4f** have *IC_50_* values in the range of 0.033 to 0.046 µM and are between 110 and 75 times more active. Unexpectedly, the best-scored compound **8b** is the least active among the 5 tested compounds, having *IC_50_* of 0.086 µM and only 41 times higher activity than GAL.

The correlation coefficient *r* between GoldScores and pIC_50_ (–logIC_50_) values of the novel hybrids, GAL and CU was 0.803. Omitting the outlier **8b** yielded *r* = 0.938.

### 2.8. Physicochemical Properties and PK Parameters

The molecular weights (*Mw*) of the tested compounds were in the range from 459.58 to 523.62 g/mol (Table 2). The *pK_a_* values varied in a short range from 6.58 to 7.78 and were close to the *pK_a_* of GAL 7.92. The GAL-CU hybrids were more lipophilic than GAL—their *logP* values were between 4.48 and 4.99, *logD_7.4_*s – between 3.92 and 4.83, while the corresponding values of GAL were 1.75 and 1.12. According to *f_B_*, compounds **4a**, **4b**, **4e** and **4f** were moderate bases, 70% ionized at physiological pH like GAL, but **8b** was a very weak base—only 13% is ionized. Although the hybrids were more lipophilic than GAL, they had polar surface areas (*PSA*) wider than that of GAL, but lower if normalized to unit mass. There was only one hydrogen-bond donors (*HBD*) in the molecules (H from ammonium cation), while the number of hydrogen-bond acceptors (*HBA*) depended on the number of O-atoms, but for all active hybrids, was up to 10.

The predicted steady state volume of distribution (*VD^ss^*) values of the hybrids **4a**, **4b**, **4e** and **4f** were almost three times bigger than the *VD^ss^* of **8b**. All compounds had comparable lipophilicity, but different ionized fractions at pH 7.4. The fraction ionized as a base affects positively *VD^ss^* [49,50,51]. *VD^ss^* reflects the drug ability to cross membranes and to bind in tissues [52]. The moderate bases **4a**, **4b**, **4e** and **4f** have *VD^ss^* between 4.71 L/kg and 5.88 L/kg which are close to the mean value of *VD^ss^* 5.90 L/kg for bases in Obach’s database [53,54]. The very weak base **8b** has *VD^ss^* of 1.85 L/kg, which is close to the mean *VD^ss^* of 1.94 L/kg for neutral molecules [54,55].

The tested compounds bind extensively to plasma proteins (PP). The predicted free fraction of drug in plasma (*f_u_*) were between 0.029 and 0.094, i.e., the extent of PP binding is in the range 91–97%. Lipophilicity has been identified as a major determinant for the PP binding of basic drugs [56]. GAL has lower *logP* and *logD_7.4_*, which results in a lower PP binding and *f_u_* = 0.83 (Table 2).

The predicted total clearance (*CL*) values were in the range between 0.305 and 0.466 L/h/kg. Apparently, the GAL-CU hybrids are low-clearance compounds with *CL*s below 37% of hepatic blood flow (Q_H_ = 1.26 L/h/kg) [57]. The half-lives (*t_1/2_*) were calculated from the corresponding *VD^ss^* and *CL* values. For the compounds **4a**, **4b**, **4e** and **4f**, they varied between 7.01 h and 10.51 h, while the half-life of **8b** was shorter, close to that of GAL. The moderate half-lives allow convenient multiple-dose regimens.

### 2.9. Molecular Dynamics Simulation of the Complex 4b-AChE

**Figure 7:**
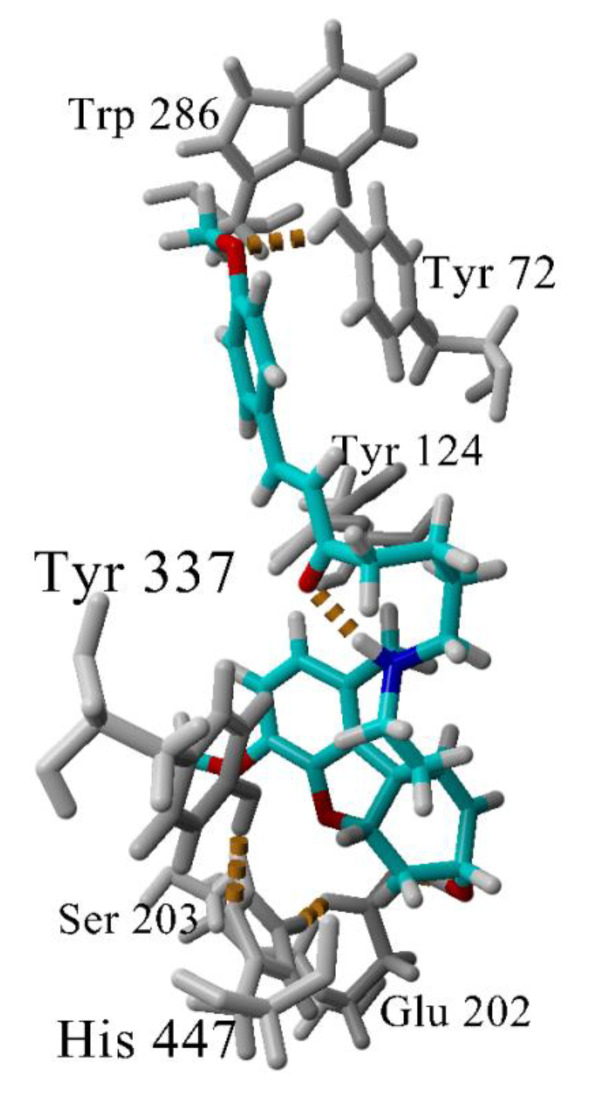
Hydrogen bonds (in yellow dashes) in the complex **4b**-AChE. Most of the time, the GAL’s ammonium group makes a hydrogen bond with Tyr337. At the given snapshot (45 ns), the N-atom makes an intramolecular hydrogen bond with the linker carbonyl oxygen. A second hydrogen bond is formed between the methoxyphenyl O-atom and Tyr72. The latter bond determines the higher affinity of **4b** to AChE.

The interactions between the most active GAL-CU hybrid **4b** with the enzyme AChE were investigated further by molecular dynamics (MD), simulating the complex **4b**-AChE for 100 ns in explicit water molecules as a solvent. The compound remained inserted within the binding site and the complex was stable during the simulation. The analysis of the MD trajectory in the vicinity of the phenyl –OCH_3_ revealed that the O-atom makes an additional hydrogen bond with Tyr72 (Figure 7) which is absent in the complex **4a**-AChE. This additional hydrogen bond might be crucial for the better binding to the enzyme.

## 3. Discussion

A combinatorial library of 72 GAL-CU hybrid compounds was designed in the present study as dual-site binding AChE inhibitors. They were constructed from GAL binding core, eight aromatic substituents mimicking the CU molecule and nine linkers, gathered in five groups. Initially, the compounds were screened for GI absorption, BBB permeability and target specificity. Forty-four compounds passed these tests and moved to molecular docking on AChE. The GoldScores of the tested compounds showed that all of them were potential good binders, better than GAL and CU themselves. The compounds were checked for synthetic feasibility and 14 of them were selected for further synthesis. The synthesized hybrids were tested initially for neurotoxicity on Neuro-2A cells, and those of them that were less toxic than CU underwent a test for affinity to AChE. The anti-AChE tests showed that all five hybrids were between 41 and 186 times more active than GAL.

**Figure 8:**
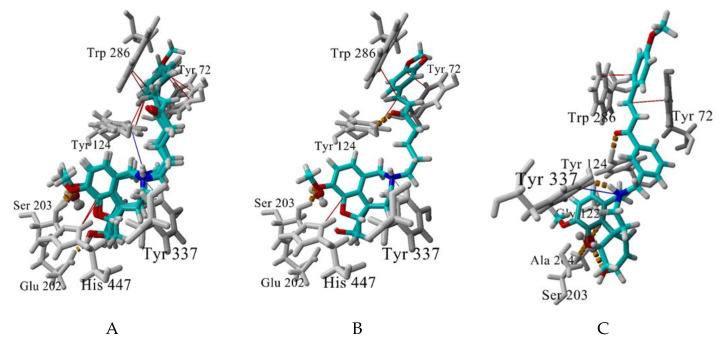
Best-scored docking poses between the compounds **4a**,**b**,**f** (**A**), **4e** (**B**), and **8f** (**C**) and AChE. Hydrogen bonds are shown in yellow dashes, cation-π interactions are shown in blue lines and π-π (including p-π) interactions, are given in red lines. The structural water molecule within the binding site is presented in ball-and-stick. Interactions are visualized by YASARA (http://www.yasara.org).

The best-scored docking poses of the tested for AChE affinity compounds **4a**, **4b**, **4e**, **4f** and **8b** and their interactions with the enzyme are visualised at Figure 8. The GAL binding core is well embedded in the bottom of AChE gorge, forming a dense network of hydrogen bonds: between GAL’s ammonium group and Tyr337, between OCH_3_ and Ser203, and between OH and Glu202. The structural water molecule here is involved in the network as well. A cation-π interaction is formed between the positively charged ammonium atom and Tyr337 and π-π contacts between the aromatic ring and His447 (Figure 8A,B). An extra T-shaped π-π contact is formed between the aromatic rings of **4a**, **4b** and **4f** and Tyr124 (Figure 8A).

The linker also takes part in the interaction with the enzyme. The sp^2^ C atoms make p-π contacts with Tyr72 and/or Trp286. Additionally, the oxygen atom of the carbonyl group forms a hydrogen bond with Tyr124 (**4e** and **8b**, Figure 8B,C). Surprisingly, no π-π stacking was observed between the linker’s benzene and any of the aromatic residues in the binding site. The terminal aromatic moieties fit well into the PAS. There are two different orientations, but all of them make π-π stacking with Trp286 and/or Tyr72.

The newly synthesized GAL-CU hybrids are weak bases, highly lipophilic, moderate or weakly ionized at physiological pH. They bind to plasma proteins in >90%, distribute extensively, cross the BBB, and are cleared slowly with half-lives up to 12 h.

A special attention deserves compound **4b** as the most active and the least toxic compound in the set. It is very close to **4a** and **4f**, having only one additional methoxy group bound in the terminal phenyl ring. The molecular docking did not detect any significant difference between **4a**, **4b** and **4f** in their interactions with the enzyme AChE. However, the two-and-a-half-fold difference in the activities compared to **4a** made us look at the complex **4b**-AChE by MD. The MD simulation showed that the phenyl -OCH_3_ group of **4b** forms an additional hydrogen bond with Tyr72 (Figure 7). At some of the initial frames, His287 also is coming quite close to -OCH_3_ but not enough to form a long-lasting hydrogen bond. The additional hydrogen bond with Tyr72 which is absent in the complex **4a**-AChE explains the better binding of **4b** to AChE.

In summary, the designed, synthesized and tested in the present study hybrid molecules between GAL and CU, showed higher affinities to AChE and less neurotoxicities than both galantamine and curcumin themselves. The novel structures have optimal ADME properties and are able to cross the BBB. They could be considered as perspective multi-target drug candidates. The most active compound **4b** will be directed to more detailed in vitro and in vivo studies.

## 4. Materials and Methods

### 4.1. Compounds

Galantamine hydrobromide was purchased from Galen-N Ltd., Sofia, Bulgaria. 1,5-Pentandiol, 98%; 1,3-benzenedimethanol, 98%; acetophenone, 98%; benzaldehyde, 98%; 4-methoxybenzaldehyde, 98%; piperonyl alcohol, 98%; 4-methylbenzaldehyde, 97%; 3-methylbenzaldehyde, 97%; 3,4-dimethylbenzaldehyde; iodine, resublimiert p.a.; triphenylphosphine, 99%; L-(-)-proline, 99+%; *tert*-butyldimethylchlorsilan, 98%; manganese(IV) oxide, 88%, electrolytically precipitated, active; sodium hydride, 60% dispersion in mineral oil; pyridinium chlorochromate, 98%; lithium hydroxide monohydrate, 98+%; hydrobromic acid, pure, ca. 48 wt% solution in water were purchased from Acros Organics, Alfa Aeser or Merck, Sofia, Bulgaria. 

### 4.2. ADME Prediction

Two free online servers were used to predict blood brain barrier (BBB) permeability of the studied compounds—SwissADME (http://www.swissadme.ch) [58] and BBB Predictor (https://www.cbligand.org/BBB/predictor.php) [59]. SwissADME server predicts permeability based on BOILED-Egg (brain or intestinal estimated permeation) method, based on the lipophilicity and polarity of small molecules [60]. The BBB predictor uses support vector machine (SVM) and LiCABEDS (ligand classifier of adaptively boosting ensemble decision stumps) algorithms on four types of fingerprints for 1593 compounds with known BBB permeability [61]. Additionally, GI absorption and PAINS (pan assay interference structures) alerts of the compounds were checked by SwissADME online platform. The GI absorption prediction is based on the BOILED-Egg method [60]. PAINS alerts help identification of frequent hitters or promiscuous compounds in many biochemical high-throughput screens based on substructural features [62].

### 4.3. Virtual Screening by Molecular Docking

The hybrid molecules were constructed with Avogadro software and minimized with MMFF94s force field, then structures were docked into the X-ray structure of human recombinant acetylcholinesterase (*rh*AChE, pdb ID: 4EY6, R = 2.40 Å) [20]. The docking simulations were performed by GOLD v.5.2.2 (CCDC Ltd., Cambridge, UK) using a protocol previously optimized in terms of scoring function, rigid/flexible ligand and binding site, radius of the binding site, presence/absence of a water molecule (HOH846) within the binding site, number of genetic algorithm (GA) runs [41,42,43,44]. The docking simulations in the present study were performed at the following settings: scoring function GoldScore, flexible ligand, flexible binding site, radius of the binding site 10 Å, a structural water molecule within the binding sire (HOH 846), 100 GA runs. The following amino acid residues were set as flexible during docking calculations: Tyr72, Asp74, Trp86, Tyr124, Ser125, Trp286, Phe297, Tyr337, Phe338, Tyr341. GAL from X-ray structure was used as a referent molecule. The best-scored compounds were analyzed for synthetic feasibility and protein-ligand interactions. 

### 4.4. Neurotoxicity Test

Murine neuroblastoma Neuro-2A cells (German collection DSMZ, Braunschweig, Germany) were cultivated under standard conditions: complete medium (90% DMEM, 10% heat-inactivated FBS, and non-essential amino acids); 37 °C and 5% CO_2_ in fully humidified atmosphere. The cell line was kept in the logarithmic growth phase by splitting 1:4 once a week using trypsin/EDTA. About 30% of the cells grew like neuronal cells. For the experimental evaluation of the cytotoxicity Neuro-2A, cells were plated in 96-well flat-bottomed cell culture plates at the recommended density of 1 × 10^6^ cells/25cm^2^. After 24 h, the cells were treated with various concentrations of the investigational compounds, and after 72-h incubation, an MTT-dye reduction assay was performed [63]. Briefly, at the end of incubation, an MTT stock solution (10mg/mL in PBS) was added (0.01 mL/well). Plates were further incubated at 37 °C for 4 h. Next, the formazan crystals were dissolved by the addition of 0.110 mL/well 5% formic acid in 2-propanol (*v*/*v*). Absorption was measured at 580nm wavelength on an automated ELISA reader Labexim LMR1. At least six wells per concentration were used, and data were processed using the GraphPad Prism 5.0 software 2.0 (San Diego, CA, USA).

### 4.5. Assessment of AChE Inhibitory Activity 

AChE activity was assayed as described by Ellman et al. [64], with some modifications [65]. Fifteen μL of *Electrophorus electricus* AChE (Sigma-Aldrich, Darmstadt, Germany) in buffer phosphate (pH 7.6) and 15 μL of the tested compounds (1.4–4350 μM in methanol) were dissolved in 200 μL in the same buffer, and plated in 96-well plates. The mixtures were incubated for 30 min at room temperature before the addition of 30 μL of the substrate solution (15 μL 0.5 M DTNB, 15 μL 0.6 mM ATCI in buffer, pH 7.6). The absorbance was read on a Microplate Reader Biochrom EZ 800 at 403 nm, after three minutes. Enzyme activity was calculated as a percentage compared to an assay using a buffer without any inhibitor, according to the following equation:

AChE inhibition (%) = ((Abs_control_ − Abs_sample_)/Abs_control_) × 100 (1)

where Abs_control_ is the absorbance of the control, containing 15 μL enzyme in buffer, 200 μL buffer and 15 μL solvent (methanol/water for GAL); Abs_sample_ is the absorbance of the sample, containing 15 μL enzyme in buffer, 200 μL buffer and 15 μL solution of the tested compound (in methanol/water for GAL).

### 4.6. Calculation of Physicochemical Properties and Prediction of Pharmacokinetic (PK) Parameters

The main physicochemical properties *pK_a_*, *logP*, *logD_7.4_*, *PSA*, number of *HBD* and *HBA* in the molecules of the tested compounds were calculated using ACD/logD v. 9.08 (ACD Inc., Toronto, Canada). As the novel hybrids are weak bases, at physiological pH they exist as cations as well as neutral molecules. The ionized fraction at pH = 7.4 (*f_BH+_*) was calculated according to the equation:

(2) $$f_{BH^{+}}=\frac{1}{1+10^{\left(7.4-pK_{a}\right)}}.$$

The key PK parameters were predicted by quantitative structure-activity relationships (QSPRs) models derived previously [53,54,55,56,57]. Three PK parameters were modelled: the steady state volume of distribution (*VD^ss^*), free fraction of drug in plasma (*f_u_*) and unbound clearance (*CL_u_*). The *CL* and half-life (*t*_1/2_) were calculated following the equations:

(3) $$CL_{u}=\frac{CL}{f_{u}}$$

(4) $$t_{1/2}=\frac{ln2\times VD^{ss}}{CL}$$

The AChEI galantamine (GAL) is given as a reference compound.

### 4.7. Molecular Dynamics Protocol

The best-scored pose of the most active hybrid in complex with AChE was used as a starting structure for the MD simulation by Amber 18 [66]. The small molecule was parametrized using GAFF2.11 force field and AM1-BCC charges, and the complex was solvated in truncated octahedral box with TIP3P water and 0.15 M NaCl. The system was subjected to energy minimization, heating to 310 K, density equilibration, preproduction equilibration and production dynamics for 100 ns. Frames were saved every 0.1 ns. 

## Figures and Tables

**Table 1:** GoldScore, neurotoxicity and affinity to AChE of the GAL-CU hybrids. The IC_50_ values of the hybrids, less toxic than CU, are given in bold. The binding affinity to AChE was measured only for the less toxic compounds.

ID	GoldScore	*IC_50_* µM Neuro-2A	*IC_50_* µM *ee*AChE	Times More Active Than GAL
**8c**	114.54	25.55	-	-
**6a**	114.50	12.14	-	-
**8g**	111.92	17.80	-	-
**6b**	111.15	7.91	-	-
**8f**	109.22	24.32	-	-
**8h**	108.95	25.00	-	-
**8b**	104.01	**28.87**	0.086	41
**4h**	96.98	21.93	-	-
**4e**	96.07	**42.91**	0.036	98
**4f**	95.05	**34.35**	0.033	110
**4b**	94.31	**52.53**	0.020	186
**4a**	93.74	**30.65**	0.046	75
**4g**	90.71	23.68	-	-
**4c**	88.28	24.11	-	-
**GAL**	74.56 ^a^	>50 ^a^	3.520	1
**CU**	88.93	26.30	67.69 ^b^	

^a^ [37]; ^b^ [48].

**Table 2:** Physicochemical properties and PK parameters of the active GAL-CU hybrids.

Parameter	4a	4b	4e	4f	8b	GAL
***Mw***	459.58	489.60	503.59	473.60	523.62	287.35
***pK_a_***	7.78	7.77	7.77	7.77	6.58	7.92
***logP***	4.53 ± 0.8	4.48 ± 0.81	4.64 ± 0.85	4.99 ± 0.80	4.9 ± 0.85	1.75
***logD_7.4_***	3.97	3.92	4.09	4.43	4.83	1.12
***PSA***	59.00	68.23	77.46	59.00	68.23	41.93
***HBD***	1	1	1	1	1	1
***HBA***	5	6	7	5	6	4
***f_BH+_***	0.71	0.70	0.70	0.70	0.13	0.77
***VD^ss^L/kg***	4.71	4.95	5.88	5.31	1.85	2.30 ^a^
***f_u_***	0.082	0.085	0.094	0.078	0.029	0.83 ^a^
***CL, L/h/kg***	0.466	0.412	0.388	0.445	0.305	0.336 ^a^
***t_1/2_**, **h***	7.01	8.32	10.51	8.28	4.19	4.74

^a^ [54].

## Supplementary Materials

The supplementary material contains: synthesis and analytical data of compounds **4a**–**c**,**e**–**h**; synthesis and analytical data of compounds **6a**,**b**; synthesis and analytical data of compounds **8b**,**c**,**f**,**g**,**h**; copies of ^1^H and ^13^C NMR spectra for the target compounds.
