# Supplementary material for: Galantamine-Curcumin Hybrids as Dual-Site Binding Acetylcholinesterase Inhibitors
Source: Molecules. 2020 Jul 23;25(15):3341. doi: 10.3390/molecules25153341 (PMC7435983; doi:10.3390/molecules25153341)
Supplement: Supplementary file 1 [file molecules-25-03341-s001.pdf]

Supplementary Material

Galantamine-curcumin hybrids as dual-site binding acetylcholinesterase inhibitors

Contents:

1. Synthesis and analytical data of compounds **4a-c,e-h**.
2. Synthesis and analytical data of compounds **6a,b**.
3. Synthesis and analytical data of compounds **8b,c,f,g,h**.
4. Copies of ^{1}H and ^{13}C NMR spectra for the target compounds.

General

Reagents were commercial grade and used without further purification. Thin layer chromatography (TLC) was performed on aluminum sheets pre-coated with Merck Kieselgel 60 F254 0.25 mm (Merck). Flash column chromatography was carried out using Silica Gel 60 230-400 mesh (Fluka). Commercially available solvents were used for reactions, TLC and column chromatography. Melting points were determined in a capillary tube on BUCHI Melting Point B-535 Apparatus 220v (uncorrected). Optical rotation ($[\alpha]_D^{20}$) were measured on JASCO P-2010 polarimeter. The NMR spectra were recorded on a Bruker Avance II+ 600 (600.13 for ^{1}H MHz and 150.92 MHz for ^{13}C NMR) spectrometer with TMS as internal standards for chemical shifts (δ, ppm). Moreover, ^{1}H and ^{13}C NMR data are reported as follows: chemical shift, multiplicity (s = singlet, d = doublet, t = triplet, q = quartet, br = broad, m = multiplet), coupling constants (Hz), integration, identification. The assignment of the ^{1}H and ^{13}C NMR spectra was made on the basis of COSY, HSQC and HMBC experiments. LC–MS analyses were performed using a Q Exactive Plus Orbitrap Mass spectrometer (Thermo Fisher Scientific, Bremen, Germany), equipped with an electrospray (ESI) probe. The spectra were recorded on a positive mode using a MS Full Scan mode.

1. Synthesis and analytical data of compounds **4a-c,e-h**.

6-bromohexan-2-ol

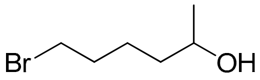

To a stirred solution of 5-bromopentanal (0.347 g, 2.10 mmol) in dry THF (9 ml) under argon atmosphere was added dropwise at 0 °C CH_3MgCl (3M in THF, 1.15 ml, 3.45 mmol). The reaction was stirred for 30 min at r.t., cooled down again to 0 °C and quenched with 10 ml sat.aq.NH_4Cl. The resulting mixture was extracted three times with ether, and the combined organic extracts were dried over $MgSO_4$, filtered and concentrated under reduced pressure. The residue was purified by flash column chromatography (silica gel, petroleum ether/EtOAc = 2:1), to give the product in 87% yield. ^1H NMR ($CDCl_3$, 600 MHz): δ = 3.84-3.79 (m, 1H, H-2), 3.43 (t, *J* = 6.7 Hz, 2H, H-6), 1.92-1.91 (m, 2H, H-3), 1.50-1.45 (m, 4H, H-4, H-5), 1.21 (d, *J* = 6.2 Hz, 3H, H-1) ppm.

6-bromohexan-2-one **11b**

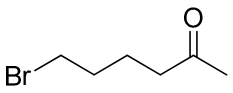

A suspension of 6-bromohexan-2-ol (0.210 g, 1.16 mmol), PCC (0.500 g, 2.32 mmol) and Celite (0.500 g) in CH_2Cl_2 (8 ml) was stirred for 20 h at r.t. The mixture was diluted with petroleum ether (20 ml) and filtered through a pad of Celite. The filtrate was concentrated under reduced pressure and subjected to flash column chromatography (silica gel, CH_2Cl_2) to give the product in 78% yield. ^1H NMR ($CDCl_3$, 600 MHz): δ = 3.41 (t, *J* = 6.7 Hz, 2H, H-6), 2.48 (t, *J* = 7.3 Hz, 2H, H-3), 2.15 (s, 3H, H-1), 1.89-1.84 (m, 2H, H-5), 1.75-1.70 (m, 2H, H-4) ppm.

General procedure for the synthesis of compounds **17a-c,e-h**

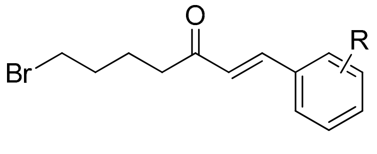

A solution of 6-bromohexan-2-one **11b** (0.179 g, 1 mmol), appropriate benzaldehyde **12a-c,e-h** (1.2 mmol), L-proline (0.017 g, 0.15 mmol) and NEt_3 (0.030 g, 0.03 mmol) in methanol (2.5 ml) was stirred at r.t. for 5 days. The mixture was concentrated till dry and directly subjected to flash column chromatography (silica gel, petroleum ether/EtOAc = 20:1 to 10:1) to give the desired product.

(*E*)-7-bromo-1-phenylhept-1-en-3-one **17a**

Yield: 53%; white crystals; m.p. 65-67 °C. ^1H NMR ($CDCl_3$, 600 MHz): δ = 7.57 (d, *J* = 16.2 Hz, 1H, H-1), 7.56-7.55 (m, 2H, arom.), 7.41-7.40 (m, 3H, arom.), 6.75 (d, *J* = 16.2 Hz, 1H, H-2), 3.45 (t, *J* = 6.7 Hz, 2H, H-7), 2.72 (t, *J* = 7.2 Hz, 2H, H-4), 1.96-1.91 (m, 2H, H-6), 1.87-1.82 (m, 2H, H-5) ppm. ^{13}C NMR ($CDCl_3$, 150.9 MHz): δ = 199.64 (CO), 142.68 (C-1), 134.38 (C, arom.), 130.52 (2CH, arom.), 128.95 (2CH, arom.), 128.27 (CH, arom.), 125.96 (C-2), 39.65 (C-4), 33.33 (C-7), 32.11 (C-6), 22.71 (C-5) ppm.

(*E*)-7-bromo-1-(4-methoxyphenyl)hept-1-en-3-one **17b**

Yield: 57%; white crystals; m.p. 67-69 °C. ^{1}H NMR ($CDCl_3$, 600 MHz): δ = 7.53 (d, *J* = 16.2 Hz, 1H, H-1), 7.50 (d, *J* = 8.5 Hz, 2H, arom.), 6.92 (d, *J* = 8.8 Hz, 2H, arom.), 6.63 (d, *J* = 16.2 Hz, 1H, H-2), 3.85 (s, 3H, OCH_3), 3.44 (t, *J* = 6.5 Hz, 2H, H-7), 2.69 (t, *J* = 7.0 Hz, 2H, H-4), 1.99-1.80 (m, 4H, H-6, H-5) ppm. ^{13}C NMR ($CDCl_3$, 150.9 MHz): δ = 199.51 (CO), 161.66 (C, arom.-OCH_3), 142.44 (C-1), 129.98 (2CH, arom.), 127.15 (C, arom.), 123.89 (C-2), 114.46 (2CH, arom.), 55.39 (OCH_3), 39.57 (C-4), 33.25 (C-7), 32.22 (C-6), 22.92 (C-5) ppm.

(*E*)-7-bromo-1-(3-methoxyphenyl)hept-1-en-3-one **17c**

Yield: 50%; colorless oil. ^{1}H NMR ($CDCl_3$, 600 MHz): δ = 7.53 (d, *J* = 16.1 Hz, 1H, H-1), 7.32 (t, *J* = 7.9 Hz, 1H, arom.), 7.15 (d, *J* = 7.6 Hz, 1H, arom.), 7.07 (br, 1H, arom.), 6.95 (dd, *J* = 8.2, 2.5 Hz, 1H, arom.), 6.72 (d, *J* = 16.1 Hz, 1H, H-2), 3.84 (s, 3H, OCH_3), 3.45 (t, *J* = 6.6 Hz, 2H, H-7), 2.72 (t, *J* = 7.2 Hz, 2H, H-4), 1.96-1.91 (m, 2H, H-6), 1.87-1.83 (m, 2H, H-5) ppm. ^{13}C NMR ($CDCl_3$, 150.9 MHz): δ = 199.62 (CO), 159.89 (C, arom.-OCH_3), 142.59 (C-1), 135.76 (C, arom.), 129.94 (CH, arom.), 126.23 (C-2), 120.99 (CH, arom.), 116.36 (CH, arom.), 113.03 (CH, arom.), 55.30 (OCH_3), 39.61 (C-4), 33.25 (C-7), 32.10 (C-6), 22.69 (C-5) ppm.

(*E*)-1-(benzo[d][1,3]dioxol-5-yl)-7-bromohept-1-en-3-one **17e**

Yield: 41%; white crystals; m.p. 77-79 °C. ^{1}H NMR ($CDCl_3$, 600 MHz): δ = 7.48 (d, *J* = 16.1 Hz, 2H, H-1), 7.06 (d, *J* = 1.7 Hz, 1H, arom.), 7.04 (dd, *J* = 8.1, 1.6 Hz, 1H, arom.), 6.83 (d, *J* = 8.0 Hz, 1H, arom.), 6.58 (d, *J* = 16.1 Hz, 1H, H-2), 6.02 (s, 2H, OCH_2O), 3.44 (t, *J* = 6.6 Hz, 2H, H-7), 2.68 (t, *J* = 7.2 Hz, 2H, H-4), 1.95-1.90 (m, 2H, H-6), 1.86-1.81 (m, 2H, H-5) ppm. ^{13}C NMR ($CDCl_3$, 150.9 MHz): δ = 199.48 (CO), 149.84 (C, arom.), 148.40 (C, arom.), 142.46 (C-1), 128.79 (C, arom.), 124.94 (C-2), 124.05 (CH, arom.), 108.62 (CH, arom.), 106.47 (CH, arom.), 101.61 (OCH_2O), 39.68 (C-4), 33.36 (C-7), 32.13 (C-6), 22.79 (C-5) ppm.

(*E*)-7-bromo-1-(4-methylphenyl)hept-1-en-3-one **17f**

Yield: 49%; white crystals; m.p. 78-80 °C. ^{1}H NMR ($CDCl_3$, 600 MHz): δ = 7.54 (d, *J* = 16.1 Hz, 2H, H-1), 7.45 (d, *J* = 8.2 Hz, 2H, arom.), 7.21 (d, *J* = 7.9 Hz, 2H, arom.), 6.70 (d, *J* = 16.1 Hz, 1H, H-2), 3.45 (t, *J* = 6.6 Hz, 2H, H-7), 2.70 (t, *J* = 7.2 Hz, 2H, H-4), 2.38 (s, 3H, CH_3), 1.96-1.91 (m, 2H, H-6), 1.87-1.81 (m, 2H, H-5) ppm. ^{13}C NMR ($CDCl_3$, 150.9 MHz): δ = 199.74 (CO), 142.74 (C-1), 141.04 (C, arom.), 131.62 (C, arom.), 129.69 (2CH, arom.), 128.28 (2CH, arom.), 125.04 (C-2), 39.55 (C-4), 33.36 (C-7), 32.14 (C-6), 22.77 (C-5), 21.49 (CH_3) ppm.

(*E*)-7-bromo-1-(3-methylphenyl)hept-1-en-3-one **17g**

Yield: 45%; white crystals; m.p. 58-60 °C. ^{1}H NMR ($CDCl_3$, 600 MHz): δ = 7.54 (d, *J* = 16.1 Hz, 2H, H-1), 7.37-7.35 (m, 2H, arom.), 7.29 (t, *J* = 7.5 Hz, 1H, arom.), 7.22 (d, *J* = 7.5 Hz, 1H, arom.), 6.73 (d, *J* = 16.1 Hz, 1H, H-2), 3.45 (t, *J* = 6.6 Hz, 2H, H-7), 2.71 (t, *J* = 7.2 Hz, 2H, H-4), 2.38 (s, 3H, CH_3), 1.96-1.91 (m, 2H, H-6), 1.87-1.82 (m, 2H, H-5) ppm. ^{13}C NMR ($CDCl_3$, 150.9 MHz): δ = 199.67 (CO), 142.87 (C-1), 138.62 (C, arom.), 134.33 (C, arom.), 131.37 (1CH,

arom.), 128.90 (1CH, arom.), 128.83 (1CH, arom.), 125.78 (C-2), 125.49 (1CH, arom.), 39.62 (C-4), 33.34 (C-7), 32.12 (C-6), 22.74 (C-5), 21.31 (CH_3) ppm.

(*E*)-7-bromo-1-(3,4-dimethylphenyl)hept-1-en-3-one **17h**

Yield: 48%; white crystals; m.p. 61-63 °C. ^{1}H NMR ($CDCl_3$, 600 MHz): δ = 7.52 (d, *J* = 16.2 Hz, 1H, H-1), 7.32-7.26 (m, 2H, arom.), 7.15 (d, *J* = 7.6 Hz, 1H, arom.), 6.70 (d, *J* = 16.2 Hz, 1H, H-2), 3.44 (t, *J* = 6.4 Hz, 2H, H-7), 2.70 (t, *J* = 6.9 Hz, 2H, H-4), 2.29 (s, 6H, 2CH_3), 1.99-1.77 (m, 4H, H-6, H-5) ppm. ^{13}C NMR ($CDCl_3$, 150.9 MHz): δ = 199.64 (CO), 142.94 (C-1), 139.78 (C, arom.), 137.20 (C, arom.), 132.13 (C, arom.), 130.26 (CH, arom.), 129.50 (CH, arom.), 125.94 (C-2), 125.00 (CH, arom.) 39.56 (C-4), 33.25 (C-7), 32.21 (C-6), 22.87 (C-5), 19.80 (CH_3), 19.70 (CH_3) ppm.

General procedure for the synthesis of compounds **4a-c,e-h**.

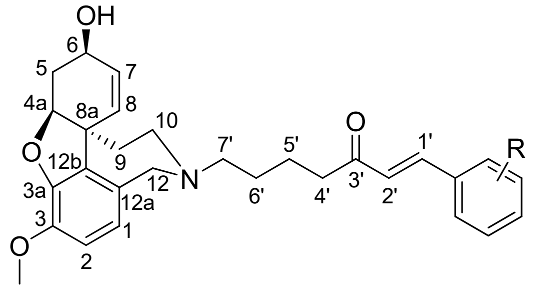

To a solution of norgalanthamine **10** (0.082 g, 0.3 mmol) in anhydrous acetonitrile (10 mL) under argon atmosphere was added appropriate bromide **17a-c,e-h** (0.3 mmol) and anhydrous K_2CO_3 (0.124 g, 0.9 mmol). After stirring for 24 h at 60 °C, the mixture was filtered through a pad of Celite. The filtrate was concentrated under reduced pressure and subjected to purification by flash column chromatography on silica gel (CH_2Cl_2/CH_3OH/NH_4OH = 50/1/0.05) to give the desired product.

(1*E*)-7-[(4a*S*,6*R*,8a*S*)-6-hydroxy-3-methoxy-5,6,9,10-tetrahydro-4a*H*-[1]benzofuro[3a,3,2-ef][2]benzazepin-11(12*H*)-yl]-1-phenylhept-1-en-3-one **4a**

Yield: 64%; white crystals; m.p. 43-44°C. $[\alpha]^{20}_D$ = -93.7 (c 1.071, $CHCl_3$). ^{1}H NMR ($CDCl_3$, 600 MHz) δ = 7.54 (d, *J* = 16.1 Hz, 1H, H-1′), 7.55-7.53 (m, 2H, arom.), 7.40-7.39 (m, 3H, arom.), 6.71 (dd, *J* = 16.1, 0.4 Hz, 1H, H-2′), 6.65 (d, *J* = 8.2 Hz, 1H, H-2), 6.62 (d, *J* = 8.2 Hz, 1H, H-1), 6.08 (d, *J* = 10.3 Hz, 1H, H-8), 6.00 (dd, *J* = 10.3, 5.0 Hz, 1H, H-7), 4.60 (br, 1H, H-6), 4.14-4.12 (m, 1H, H-4a), 4.13 (d, *J* = 15.4 Hz, 1H, H-12), 3.83 (d, *J* = 15.4 Hz, 1H, H-12), 3.82 (s, 3H, OCH_3), 3.36 (t, *J* = 13.7 Hz, 1H, H-10), 3.18 (d, *J* = 14.6 Hz, 1H, H-10), 2.70-2.67 (m, 1H, H-5), 2.68 (t, *J* = 7.3 Hz, 1H, H-4′), 2.58-2.53 (m, 1H, H-7′), 2.53-2.48 (m, 1H, H-7′), 2.41 (br, 1H, OH), 2.05-2.02 (m, 1H, H-9), 2.02-1.98 (m, 1H, H-5), 1.73-1.64 (m, 2H, H-5′), 1.57-1.51 (m, 3H, H-6′, H-9) ppm. ^{13}C NMR ($CDCl_3$, 150.9 MHz) δ = 200.22 (CO), 145.74 (C-3a), 144.07 (C-3), 142.40 (C-1′), 134.46 (C arom.), 133.09 (C-12b), 133.43 (CH arom., C-12a), 128.92 (2CH arom.), 128.21 (2CH arom.), 127.58 (C-7), 126.84 (C-8), 126.07 (C-2′), 122.03 (C-1), 111.06 (C-2), 88.69 (C-6), 62.04 (C-4a), 57.63 (C-12), 55.83 (OCH_3), 51.55 (C-10), 51.00 (C-7′), 48.35 (C-8a), 40.59 (C-4′), 32.87 (C-9), 29.89 (C-5), 26.83 (C-6′), 21.99 (C-5′) ppm. HRMS found for $C_{29}H_{34}NO_4$: m/z 460.2478 $[M+H]^+$, calcd. m/z 460.2482.

(1*E*)-7-[(4a*S*,6*R*,8a*S*)-6-hydroxy-3-methoxy-5,6,9,10-tetrahydro-4a*H*-[1]benzofuro[3a,3,2-ef][2]benzazepin-11(12*H*)-yl]-1-(4-methoxyphenyl)hept-1-en-3-one **4b**

Yield: 51%; white crystals; m.p. 44-46°C. $[\alpha]^{20}_{D}$ = -84.4 (c 1.000, $CHCl_3$). ^{1}H NMR ($CDCl_3$, 600 MHz) δ = 7.50 (d, *J* = 16.1 Hz, 1H, H-1′), 7.49 (d, *J* = 8.7 Hz, 2H, arom.), 6.91 (d, *J* = 8.8 Hz, 2H, arom.), 6.65 (d, *J* = 8.2 Hz, 1H, H-2), 6.62 (d, *J* = 8.2 Hz, 1H, H-1), 6.61 (d, *J* = 16.1 Hz, 1H, H-2′), 6.08 (d, *J* = 10.2 Hz, 1H, H-8), 6.00 (dd, *J* = 10.2, 4.6 Hz, 1H, H-7), 4.61 (br, 1H, H-6), 4.14-4.12 (m, 1H, H-4a), 4.13 (d, *J* = 15.3 Hz, 1H, H-12), 3.85 (s, 3H, OCH_3), 3.84-3.81 (m, 1H, H-12), 3.82 (s, 3H, OCH_3), 3.35 (t, *J* = 13.7 Hz, 1H, H-10), 3.18 (d, *J* = 14.9 Hz, 1H, H-10), 2.70-2.66 (m, 1H, H-5), 2.65 (t, *J* = 7.3 Hz, 1H, H-4′), 2.58-2.52 (m, 1H, H-7′), 2.55-2.48 (m, 1H, H-7′), 2.43 (br, 1H, OH), 2.07-2.02 (m, 1H, H-9), 2.02-1.98 (m, 1H, H-5), 1.72-1.63 (m, 2H, H-5′), 1.57-1.52 (m, 3H, H-6′, H-9) ppm. ^{13}C NMR ($CDCl_3$, 150.9 MHz) δ = 200.24 (CO), 161.50 (C arom.), 145.74 (C-3a), 144.06 (C-3), 142.20 (C-1′), 133.08 (C-12b), 129.92 (2CH arom., C-12a), 127.57 (C-7), 127.10 (C arom.), 126.86 (C-8), 123.91 (C-2′), 122.04 (C-1), 114.36 (2CH arom.), 111.06 (C-2), 88.69 (C-6), 62.04 (C-4a), 57.64 (C-12), 55.83 (OCH_3), 55.37 (OCH_3), 51.53 (C-10), 50.97 (C-7′), 48.35 (C-8a), 40.49 (C-4′), 32.87 (C-9), 29.89 (C-5), 26.87 (C-6′), 22.14 (C-5′) ppm. HRMS found for $C_{30}H_{36}NO_5$: m/z 490.2587 $[M+H]^+$, calcd. m/z 490.2588.

(1*E*)-7-[(4a*S*,6*R*,8a*S*)-6-hydroxy-3-methoxy-5,6,9,10-tetrahydro-4a*H*-[1]benzofuro[3a,3,2-ef][2]benzazepin-11(12*H*)-yl]-1-(3-methoxyphenyl)hept-1-en-3-one **4c**

Yield: 48%; white crystals; m.p. 40-42°C. $[\alpha]^{20}_{D}$ = -88.1 (c 1.151, $CHCl_3$). ^{1}H NMR ($CDCl_3$, 600 MHz) δ = 7.50 (d, *J* = 16.1 Hz, 1H, H-1′), 7.31 (t, *J* = 7.9 Hz, 1H, arom.), 7.13 (d, *J* = 7.6 Hz, 1H, arom.), 7.06 (s, 1H, arom.), 6.95 (dd, *J* = 8.2, 2.5 Hz, 1H, arom.), 6.71 (d, *J* = 16.1 Hz, 1H, H-2′), 6.65 (d, *J* = 8.2 Hz, 1H, H-2), 6.62 (d, *J* = 8.2 Hz, 1H, H-1), 6.08 (d, *J* = 10.3 Hz, 1H, H-8), 6.00 (dd, *J* = 10.2, 5.0 Hz, 1H, H-7), 4.61 (br, 1H, H-6), 4.14-4.12 (m, 1H, H-4a), 4.13 (d, *J* = 15.4 Hz, 1H, H-12), 3.84 (s, 3H, OCH_3), 3.82 (s, 3H, OCH_3), 3.83-3.81 (m, 1H, H-12), 3.36 (t, *J* = 13.6 Hz, 1H, H-10), 3.18 (d, *J* = 14.4 Hz, 1H, H-10), 2.70-2.67 (m, 1H, H-5), 2.68 (t, *J* = 7.4 Hz, 1H, H-4′), 2.58-2.52 (m, 1H, H-7′),2.55-2.48 (m, 1H, H-7′),2.41 (br, 1H, OH), 2.07-2.02 (m, 1H, H-9), 2.02-1.98 (m, 1H, H-5), 1.72-1.62 (m, 2H, H-5′), 1.62-1.51 (m, 3H, H-6′, H-9) ppm. ^{13}C NMR ($CDCl_3$, 150.9 MHz) δ = 200.20 (CO), 159.87 (C arom.), 145.74 (C-3a), 144.07 (C-3), 142.31 (C-1′), 135.84 (C arom.), 133.08 (C-12b), 129.91 (2CH arom., C-12a), 127.58 (C-7), 126.85 (C-8), 126.35 (C-2′), 122.03 (C-1), 120.93 (CH arom.), 116.20 (CH arom.), 113.04 (CH arom.), 111.06 (C-2), 88.69 (C-6), 62.04 (C-4a), 57.60 (C-12), 55.82 (OCH_3), 55.28 (OCH_3), 51.54 (C-10), 51.03 (C-7′), 48.35 (C-8a), 40.57 (C-4′), 32.86 (C-9), 29.89 (C-5), 26.83 (C-6′), 21.97 (C-5′) ppm. HRMS found for $C_{30}H_{36}NO_5$: m/z 490.2582 $[M+H]^+$, calcd. m/z 490.2588.

(1*E*)-1-(1,3-benzodioxol-5-yl)-7-[(4a*S*,6*R*,8a*S*)-6-hydroxy-3-methoxy-5,6,9,10-tetrahydro-4a*H*-[1]benzofuro[3a,3,2-ef][2]benzazepin-11(12*H*)-yl]hept-1-en-3-one **4e**

Yield: 51%; white crystals; m.p. 46-49°C. $[\alpha]^{20}_{D}$ = -86.9 (c 1.132, $CHCl_3$). ^{1}H NMR ($CDCl_3$, 600 MHz) δ = 7.45 (d, *J* = 16.0 Hz, 1H, H-1′),7.05 (d, *J* = 1.6 Hz, 1H, arom.), 7.02 (dd, *J* = 8.1, 1.6 Hz, 1H, arom.), 6.82 (d, *J* = 8.0 Hz, 1H, arom.), 6.65 (d, *J* = 8.2 Hz, 1H, H-2), 6.62 (d, *J* = 8.2 Hz, 1H, H-1), 6.56 (d, *J* = 16.0 Hz, 1H, H-2′), 6.08 (d, *J* = 10.0 Hz, 1H, H-8), 6.02 (s, 2H, OCH_2O), 6.00 (dd, *J* = 10.2, 4.8 Hz, 1H, H-7), 4.61 (br, 1H, H-6), 4.14-4.12 (m, 1H, H-4a), 4.13 (d, *J* = 15.1

Hz, 1H, H-12), 3.84-3.81 (m, 1H, H-12), 3.83 (s, 3H, OCH_3), 3.36 (t, *J* = 13.8 Hz, 1H, H-10), 3.19-3.16 (m,1H, H-10), 2.70-2.67 (m, 1H, H-5), 2.64 (t, *J* = 7.3 Hz, 1H, H-4′), 2.58-2.47 (m, 2H, H-7′), 2.07-2.02 (m, 1H, H-9), 2.02-1.98 (m, 1H, H-5), 1.72-1.62 (m, 2H, H-5′), 1.56-1.51 (m, 3H, H-6′, H-9) ppm. ^{13}C NMR ($CDCl_3$, 150.9 MHz) δ = 200.08 (CO), 149.75 (C arom.), 148.37 (C arom.), 145.74 (C-3a), 144.06 (C-3), 142.19 (C-1′), 130.08 (C-12b), 128.86 (C arom., C-12a), 127.58 (C-7), 126.84 (C-8), 124.84 (CH arom.), 124.16 (C-2′), 122.03 (C-1), 111.05 (C-2), 108.59 (CH arom.), 106.42 (CH arom.), 101.58 (OCH_2O), 88.69 (C-6), 62.04 (C-4a), 57.61 (C-12), 55.82 (OCH_3), 55.83 (OCH_3), 51.53 (C-10), 50.98 (C-7′), 48.35 (C-8a), 40.65 (C-4′), 32.87 (C-9), 29.89 (C-5), 26.85 (C-6′), 22.08 (C-5′) ppm. HRMS found for $C_{30}H_{34}NO_6$: m/z 504.2376 $[M+H]^+$, calcd. m/z 504.2381.

(1*E*)-7-[(4a*S*,6*R*,8a*S*)-6-hydroxy-3-methoxy-5,6,9,10-tetrahydro-4a*H*-[1]benzofuro[3a,3,2-ef][2]benzazepin-11(12*H*)-yl]-1-(4-methylphenyl)hept-1-en-3-one **4f**

Yield: 49%; white crystals; m.p. 38-41°C. $[\alpha]^{20}_D$ = -93.5 (c 0.952, $CHCl_3$). ^1H NMR ($CDCl_3$, 600 MHz) δ = 7.52 (d, *J* = 16.2 Hz, 1H, H-1′), 7.44 (d, *J* = 8.0 Hz, 2H, arom.), 7.20 (d, *J* = 8.0 Hz, 2H, arom.), 6.69 (d, *J* = 16.2 Hz, 1H, H-2′), 6.64 (d, *J* = 8.2 Hz, 1H, H-2), 6.62 (d, *J* = 8.2 Hz, 1H, H-1), 6.08 (d, *J* = 10.3 Hz, 1H, H-8), 6.00 (dd, *J* = 10.2, 5.0 Hz, 1H, H-7), 4.61 (br, 1H, H-6), 4.14-4.11 (m, 1H, H-4a), 4.13 (d, *J* = 15.2 Hz, 1H, H-12), 3.83 (d, *J* = 15.2 Hz, 1H, H-12), 3.82 (s, 3H, OCH_3), 3.35 (t, *J* = 13.7 Hz, 1H, H-10), 3.18 (d, *J* = 14.2 Hz, 1H, H-10), 2.70-2.65 (m, 1H, H-5), 2.66 (t, *J* = 7.2 Hz, 1H, H-4′), 2.58-2.51 (m, 1H, H-7′),2.55-2.48 (m, 1H, H-7′), 2.42 (br, 1H, OH), 2.38 (s, 3H, CH_3), 2.07-2.01 (m, 1H, H-9), 2.02-1.98 (m, 1H, H-5), 1.73-1.62 (m, 2H, H-5′), 1.57-1.52 (m, 3H, H-6′, H-9) ppm. ^{13}C NMR ($CDCl_3$, 150.9 MHz) δ = 200.31 (CO), 145.74 (C-3a), 144.07 (C-3), 142.47 (C-1′), 140.93 (C arom.), 133.09 (C-12b), 131.70 (C arom.), 129.66 (2CH arom., C-12a), 128.22 (2CH arom.), 127.58 (C-7), 126.86 (C-8), 125.16 (C-2′), 122.04 (C-1), 111.06 (C-2), 88.70 (C-6), 62.04 (C-4a), 57.66 (C-12), 55.83 (OCH_3), 51.54 (C-10), 51.13 (C-7′), 48.35 (C-8a), 40.51 (C-4′), 32.87 (C-9), 29.89 (C-5), 26.85 (C-6′), 22.06 (C-5′), 21.48 (CH_3) ppm. HRMS found for $C_{30}H_{36}NO_4$: m/z 474.2630 $[M+H]^+$, calcd. m/z 474.2639.

(1*E*)-7-[(4a*S*,6*R*,8a*S*)-6-hydroxy-3-methoxy-5,6,9,10-tetrahydro-4a*H*-[1]benzofuro[3a,3,2-ef][2]benzazepin-11(12*H*)-yl]-1-(3-methylphenyl)hept-1-en-3-one **4g**

Yield: 63%; white crystals; m.p. 38-40°C. $[\alpha]^{20}_D$ = -92.8 (c 1.108, $CHCl_3$). ^1H NMR ($CDCl_3$, 600 MHz) δ = 7.51 (d, *J* = 16.2 Hz, 1H, H-1′), 7.36-7.37 (m, 2H, arom.), 7.29 (t, *J* = 7.5 Hz, 1H, arom.), 7.21 (d, *J* = 7.5 Hz, 1H, arom.), 6.71 (d, *J* = 16.2 Hz, 1H, H-2′), 6.65 (d, *J* = 8.2 Hz, 1H, H-2), 6.62 (d, *J* = 8.2 Hz, 1H, H-1), 6.08 (d, *J* = 10.1 Hz, 1H, H-8), 6.00 (dd, *J* = 10.3, 4.7 Hz, 1H, H-7), 4.61 (br, 1H, H-6), 4.14 (s, 1H, H-4a), 4.13 (d, *J* = 15.4 Hz, 1H, H-12), 3.83 (d, *J* = 15.4 Hz, 1H, H-12), 3.82 (s, 3H, OCH_3), 3.36 (t, *J* = 13.8 Hz, 1H, H-10), 3.18 (d, *J* = 14.3 Hz, 1H, H-10), 2.70-2.66 (m, 1H, H-5), 2.67 (t, *J* = 7.3 Hz, 1H, H-4′), 2.58-2.52 (m, 1H, H-7′), 2.55-2.48 (m, 1H, H-7′), 2.41 (br, 1H, OH), 2.38 (s, 3H, CH_3), 2.07-2.02 (m, 1H, H-9), 2.02-1.98 (m, 1H, H-5), 1.73-1.63 (m, 2H, H-5′), 1.57-1.51 (m, 3H, H-6′, H-9) ppm. ^{13}C NMR ($CDCl_3$, 150.9 MHz) δ = 200.25 (CO), 145.74 (C-3a), 144.07 (C-3), 142.60 (C-1′), 138.59 (C arom.), 134.40 (C arom.), 133.08 (C-12b), 131.27 (CH arom.), 128.84 (CH arom., C-12a), 128.80 (CH arom.), 127.58 (C-7), 126.83 (C-8), 125.89 (C-2′), 125.44 (CH arom.), 122.04 (C-1), 111.06 (C-2), 88.69 (C-6), 62.04 (C-4a), 57.62 (C-12), 55.82 (OCH_3), 51.54 (C-10), 51.09 (C-7′), 48.35 (C-8a), 40.57 (C-4′), 32.86 (C-9),

29.89 (C-5), 26.83 (C-6′), 22.01 (C-5′), 21.30 (CH_3) ppm. HRMS found for $C_{30}H_{36}NO_4$: m/z 474.2634[M+H]$^+$, calcd. m/z 474.2639.

(1*E*)-7-[(4a*S*,6*R*,8a*S*)-6-hydroxy-3-methoxy-5,6,9,10-tetrahydro-4a*H*-[1]benzofuro[3a,3,2-ef][2]benzazepin-11(12*H*)-yl]-1-(3,4-dimethylphenyl)hept-1-en-3-one **4h**

Yield: 54%; white crystals; m.p. 48-50°C. $[\alpha]^{20}_D$ = -91.9 (c 1.154, $CHCl_3$). ^{1}H NMR ($CDCl_3$, 600 MHz) δ = 7.50 (d, *J* = 16.2 Hz, 1H, H-1′), 7.32 (s, 1H, arom.), 7.28 (dd, *J* = 7.8, 1.6 Hz, 1H, arom.), 7.15 (d, *J* = 7.8 Hz, 1H, arom.), 6.68 (d, *J* = 16.2 Hz, 1H, H-2′), 6.64 (d, *J* = 8.2 Hz, 1H, H-2), 6.62 (d, *J* = 8.2 Hz, 1H, H-1), 6.08 (d, *J* = 10.2 Hz, 1H, H-8), 6.00 (dd, *J* = 10.2, 5.1 Hz, 1H, H-7), 4.61 (br, 1H, H-6), 4.14-4.12 (m, 1H, H-4a), 4.13 (d, *J* = 15.4 Hz, 1H, H-12), 3.83 (d, *J* = 15.4 Hz, 1H, H-12), 3.82 (s, 3H, OCH_3), 3.35 (t, *J* = 13.7 Hz, 1H, H-10), 3.18 (d, *J* = 14.4 Hz, 1H, H-10), 2.70-2.66 (m, 1H, H-5), 2.66 (t, *J* = 7.3 Hz, 1H, H-4′), 2.58-2.51 (m, 1H, H-7′), 2.54-2.48 (m, 1H, H-7′), 2.42 (br, 1H, OH), 2.29 (s, 6H, $2CH_3$), 2.07-2.02 (m, 1H, H-9), 2.02-1.98 (m, 1H, H-5), 1.74-1.62 (m, 2H, H-5′), 1.57-1.51 (m, 3H, H-6′, H-9) ppm. ^{13}C NMR ($CDCl_3$, 150.9 MHz) δ = 200.34 (CO), 145.73 (C-3a), 144.04 (C-3), 142.68 (C-1′), 139.70 (C arom.), 137.18 (C arom.), 133.08 (C-12b), 132.08 (C arom.), 130.19 (CH arom.), 129.40 (CH arom.), 129.15 (C-12a), 127.55 (C-7), 126.86 (C-8), 125.88 (CH arom.), 125.01 (C-2′), 122.03 (C-1), 111.05 (C-2), 88.69 (C-6), 62.04 (C-4a), 57.62 (C-12), 55.82 (OCH_3), 51.52 (C-10), 51.11 (C-7′), 48.35 (C-8a), 40.48 (C-4′), 32.86 (C-9), 29.89 (C-5), 26.86 (C-6′), 22.07 (C-5′), 19.82 (CH_3), 19.74 (CH_3) ppm. HRMS found for $C_{31}H_{38}NO_4$: m/z 488.2788 [M+H]$^+$, calcd. m/z 488.2795.

2. Synthesis and analytical data of compounds **6a,b**.

(3-((*tert*-butyldimethylsilyloxy)methyl)phenyl)methanol

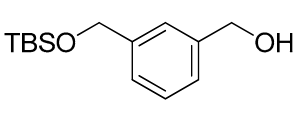

To a solution of 1,3-benzenedimethanol (0.691 g, 5 mmol) in dry THF (10 ml) under argon atmosphere was added NaH (60% in min. oil, 0.240 g, 5 mmol). After 45 min at r.t., the hydrogen evolution seized, the mixture was cooled at 0 ºC and TBDMSCl (0.754 g, 5 mmol) was added. The mixture was stirred for an additional 45 min at r.t. and diluted with ether (90 ml). It was washed with 10% aq.K_2CO_3 (40 ml) and brine (40 ml). The organic phase was dried above $MgSO_4$, filtered and concentrated under reduced pressure. The residue was purified by flash column chromatography (silica gel, petroleum ether/EtOAc = 4:1), to give the product in 52% yield. ^{1}H NMR ($CDCl_3$, 600 MHz): δ = 7.35-7.33 (m, 2H, arom.), 7.28-7.25 (m, 2H, arom.), 4.80 (s, 2H, CH_2OSi), 4.70 (s, 2H, CH_2OH), 0.95 (s, 9H, $C(CH_3)_3$), 0.12 (s, 6H, $Si(CH_2)_2$) ppm.

3-((*tert*-butyldimethylsilyloxy)methyl)benzaldehyde **18a**

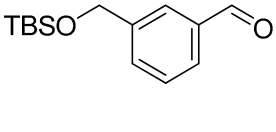

To a solution of (3-((*tert*-butyldimethylsilyloxy)methyl)phenyl)methanol (0.653 g, 2.59 mmol) in CH_2Cl_2 (35 ml) was added MnO_2 (2.59 g). The suspension was stirred overnight at r.t. and filtered through a pad of celite.

The filtrate was concentrated under reduced pressure to give the product in 90% yield. No further purification was necessary. ^{1}H NMR ($CDCl_3$, 600 MHz): δ = 10.03 (s, 1H, CHO), 7.84 (d, *J* = 0.5 Hz, 1H, arom.), 7.77 (d, *J* = 7.6 Hz, 1H, arom.), 7.61 (dd, *J* = 7.6, 0.7 Hz, 1H, arom.), 7.51 (t, *J* = 7.6 Hz, 1H, arom.), 4.81 (s, 2H, CH_2OSi), 0.96 (s, 9H, $C(CH_3)_3$), 0.12 (s, 6H, $Si(CH_2)_2$) ppm.

(*E*)-3-(3-((*tert*-butyldimethylsilyloxy)methyl)phenyl)-1-phenylprop-2-en-1-one **21**

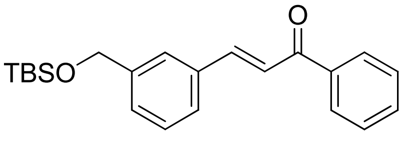

To a solution of acetophenone (0.092 g, 0.77 mmol) and 3-((*tert*-butyldimethylsilyloxy)methyl)benzaldehyde **18a** (0.183 g, 0.73 mmol) in ethanol (5 ml) was added $LiOH.H_2O$ (20 mol%, 0.006 g, 0.146 mmol), and the reaction was stirred overnight at r.t. The mixture was diluted with ether (15 ml) and washed with sat.aq.NH_4Cl, and water. The organic layers were dried above MgSO4, filtered and concentrated. The residue was purified by flash column chromatography (silica gel, petroleum ether/EtOAc = 50:1) to give the product in 71% yield as colourless oil. ^{1}H NMR ($CDCl_3$, 600 MHz): δ = 8.03-8.02 (m, 2H, arom.), 7.83 (d, *J* = 15.7 Hz, 1H, H-3), 7.63 (s, 1H, arom.), 7.61-7.58 (m, 1H, arom.), 7.55 (d, *J* = 15.7 Hz, 1H, H-2), 7.54-7.50 (m, 3H, arom.), 7.40-7.37 (m, 2H, arom.), 4.79 (s, 2H, CH_2OSi), 0.97 (s, 9H, $C(CH_3)_3$), 0.13 (s, 6H, $Si(CH_2)_2$) ppm. ^{13}C NMR ($CDCl_3$, 150.9 MHz): δ = 190.53 (CO), 144.96 (C-3), 142.29 (C, arom.-CO), 138.21 (C, arom.), 134.79 (C, arom.), 132.77 (CH, arom.), 128.84 (CH, arom.), 128.61 (2CH, arom.), 128.48 (2CH, arom.), 128.20 (CH, arom.), 127.08 (CH, arom.), 125.85 (CH, arom.), 121.94 (C-2), 64.50 (CH_2O), 25.93 ($C(\underline{C}H_3)_3$), 18.42 ($\underline{C}(CH_3)_3$), -5.25 ($Si(CH_2)_2$) ppm.

(*E*)-3-(3-(hydroxymethyl)phenyl)-1-phenylprop-2-en-1-one

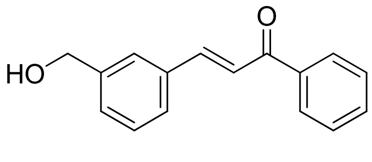

To a solution of silyl ether **21** (0.098 g, 0.278 mmol) in methanol (3 ml) was added 1N HCl (1 ml). It was initially was formed as a precipitate, which cleared in 5 minutes. The mixture was basified with 10% aq.K_2CO_3 and extracted with ether. The combined organic extracts were dried, filtered, concentrated under reduced pressure and subjected to flash column chromatography (silica gel, petroleum ether/EtOAc = 1:1) to give the product in quantitative yield. ^{1}H NMR ($CDCl_3$, 600 MHz): δ = 8.03-8.02 (m, 2H, arom.), 7.81 (d, *J* = 15.7 Hz, 1H, H-3), 7.67 (br, 1H, arom.), 7.61-7.58 (m, 1H, arom.), 7.57-7.54 (m, 1H, arom.), 7.56 (d, *J* = 15.7 Hz, 1H, H-2), 7.52-7.50 (m, 2H, arom.), 7.42-7.41 (m, 2H, arom.), 4.76 (s, 2H, CH_2O) ppm. ^{13}C NMR ($CDCl_3$, 150.9 MHz): δ = 190.53 (CO), 144.61 (C-3), 141.64 (C, arom.), 138.09 (C, arom.), 135.14 (C, arom.), 132.86 (CH, arom.), 129.17 (CH, arom.), 129.02 (CH, arom.), 128.63 (2CH, arom.), 128.51 (2CH, arom.), 127.91 (CH, arom.), 126.55 (CH, arom.), 122.24 (C-2), 64.88 (CH_2O) ppm.

(*E*)-3-(3-(iodomethyl)phenyl)-1-phenylprop-2-en-1-one **22**

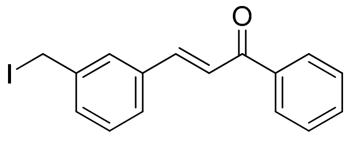

A mixture of PPh_3 (0.094 g, 0.359 mmol), imidazole (0.033 g, 0.478 mmol) and I_2 (0.057 g, 0.359 mmol) in dry THF (3 ml) was stirred under argon atmosphere for 10 min at r.t. A solution of (*E*)-3-(3-(hydroxymethyl)phenyl)-1-phenylprop-2-en-1-one in dry THF (3 ml) was added and the mixture was stirred for 1 hour at r.t. The reaction was quenched with

aq.$Na_2S_2O_3$ and extracted with ether. The combined organic extracts were washed with brine, dried, filtered, concentrated under reduced pressure and subjected to flash column chromatography (silica gel, petroleum ether/EtOAc = 20:1) to give the product in 81% yield. ^{1}H NMR ($CDCl_3$, 600 MHz): δ = 8.04-8.02 (m, 2H, arom.), 7.77 (d, J = 15.7 Hz, 1H, H-3), 7.65 (br, 1H, arom.), 7.62-7.59 (m, 1H, arom.), 7.54 (d, J = 15.7 Hz, 1H, H-2), 7.53-7.51 (m, 3H, arom.), 7.44-7.42 (m, 1H, arom.), 7.36 (t, J = 7.6 Hz, 1H, arom.), 4.48 (s, 2H, CH_2I) ppm. ^{13}C NMR ($CDCl_3$, 150.9 MHz): δ = 190.39 (CO), 144.01 (C-3), 140.18 (C, arom.), 138.02 (C, arom.), 135.47 (C, arom.), 132.90 (CH, arom.), 130.77 (CH, arom.), 129.50 (CH, arom.), 128.65 (2CH, arom.), 128.52 (2CH, arom.), 128.40 (CH, arom.), 127.96 (CH, arom.), 122.59 (C-2), 4.55 (CH_2I) ppm.

(*E*)-1-phenyl-3-(3-[(4a*S*,6*R*,8a*S*)-6-hydroxy-3-methoxy-5,6,9,10-tetrahydro-4a*H*-[1]benzofuro[3a,3,2-ef][2]benzazepin-11(12*H*)-ylmethyl]phenyl)prop-2-en-1-one **6a**

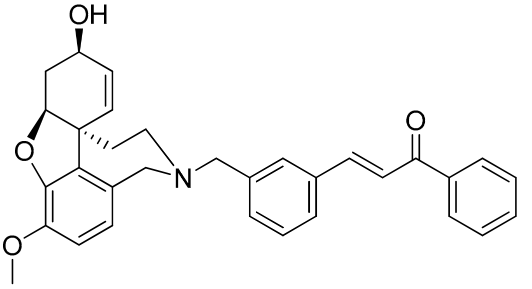

To a solution of norgalanthamine **10** (0.053 g, 0.19 mmol) in anhydrous acetonitrile (6 mL) under argon atmosphere was added iodide **22** (0.056 g, 0.16 mmol) and anhydrous K_2CO_3 (0.067 g, 0.48 mmol). After stirring for 22 h at 60 °C, the mixture was filtered through a pad of celite. The filtrate was concentrated under reduced pressure and subjected to purification by flash column chromatography on silica gel (CH_2Cl_2/CH_3OH/NH_4OH = 50/1/0.05) to give the desired product in 76% yield. White crystals; m.p. 78-82°C. $[\alpha]^{20}_D$ = -94.8 (c 0.914, $CHCl_3$). ^{1}H NMR ($CDCl_3$, 600 MHz) δ = 8.02 (d, J = 7.3 Hz, 2H, arom.), 7.81 (d, J = 15.7 Hz, 1H, H-3′), 7.61-7.58 (m, 2H, arom.), 7.55-7.50 (m, 3H, arom.), 7.51 (d, J = 15.7 Hz, 1H, H-2′), 7.39-7.34 (m, 2H, arom.), 6.66 (d, J = 8.1 Hz, 1H, H-2), 6.49 (d, J = 8.1 Hz, 1H, H-1), 6.12 (d, J = 10.2 Hz, 1H, H-8), 6.02 (dd, J = 10.2, 5.0 Hz, 1H, H-7), 4.68 (br, 1H, H-6), 4.17-4.14 (m, 1H, H-4a), 4.12 (d, J = 14.5 Hz, 1H, H-12), 3.84 (s, 3H, OCH_3), 3.72-3.70 (m, 3H, H-12, NCH_2), 3.48-3.43 (m, 1H, H-10), 3.22 (d, J = 13.8 Hz, 1H, H-10), 2.73-2.70 (m, 1H, H-5), 2.42 (br, 1H, OH), 2.16-2.12 (m, 1H, H-9), 2.04-2.00 (m, 1H, H-5), 1.56 (d, J = 12.8 Hz, 1H, H-9) ppm. ^{13}C NMR ($CDCl_3$, 150.9 MHz) δ = 190.54 (CO), 145.85 (C-3a), 144.83 (C-3′), 144.16 (C-3), 138.14 (2C arom.), 134.92 (C arom.), 133.29 (C-12b), 132.80 (CH arom.), 131.25 (CH arom.), 128.97 (CH arom., C-12a), 128.81 (CH arom.), 128.61 (2CH arom.), 128.50 (2CH arom.), 127.74 (C-7), 127.59 (CH arom.), 126.75 (C-8), 122.20 (C-1), 122.03 (C-2′), 111.11 (C-2), 88.75 (C-6), 62.04 (C-4a), 57.09 (C-12), 55.87 (OCH_3, NCH_2), 51.83 (C-10), 48.44 (C-8a), 33.15 (C-9), 29.92 (C-5) ppm. HRMS found for $C_{32}H_{32}NO_4$: m/z 494.2319 $[M+H]^+$, calcd. m/z 494.2326.

(3-(bromomethyl)phenyl)methanol

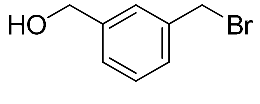

To a solution of 1,3-benzenedimethanol (1 g, 7.25 mmol) in toluene (10 ml) was added 48% aq. HBr (1 ml, 8.7 mmol). The two phase mixture was heated at 60 °C for 2 h and quenched with aq. $NaHCO_3$. It was extracted with ether and the combined organic extracts were dried above $MgSO_4$, filtered, concentrated under reduced pressure, and subjected to flash column chromatography (silica gel, petroleum

ether/EtOAc = 4:1) to give the product in 70% yield. ^{1}H NMR ($CDCl_3$, 600 MHz): δ = 7.41 (s, 1H, arom.), 7.36-7.32 (m, 2H, arom.), 7.31-7.29 (m, 1H, arom.), 4.70 (s, 2H, CH_2O), 4.50 (s, 2H, CH_2Br) ppm.

3-(bromomethyl)benzaldehyde **18b**

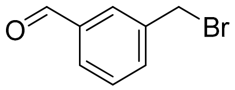

To a solution of (3-(bromomethyl)phenyl)methanol (1.021 g, 5.08 mmol) in CH_2Cl_2 (50 ml) was added MnO_2 (5.08 g). The suspension was stirred 20 h at r.t. and filtered through a pad of Celite. The filtrate was concentrated under reduced pressure and subjected to flash column chromatography (silica gel, petroleum ether/EtOAc = 9:1) to give the product in 80% yield. ^{1}H NMR ($CDCl_3$, 600 MHz): δ = 10.02 (s, 1H, CHO), 7.91 (s, 1H, arom.), 7.83 (d, *J* = 7.6 Hz, 1H, arom.), 7.67 (d, *J* = 7.6 Hz, 1H, arom.), 7.54 (t, *J* = 7.6 Hz, 1H, arom.), 4.55 (s, 2H, CH_2Br) ppm.

(*E*)-3-(3-(bromomethyl)phenyl)-1-(4-methoxyphenyl)prop-2-en-1-one **23**

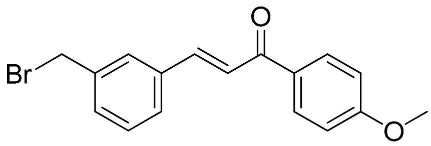

To a solution of acetanisole (0.075 g, 0.5 mmol) and 3-(bromomethyl)benzaldehyde **18b** (0.100 g, 0.5 mmol) in ethanol (5 ml) was added $LiOH.H_2O$ (20 mol%, 0.004 g, 0.1 mmol) and the reaction was stirred overnight at r.t. The mixture was concentrated under reduced pressure and directly subjected to flash column chromatography (silica gel, petroleum ether/EtOAc = 10:1) to give the product in 40% yield as white crystals; m.p. 115-117 °C. ^{1}H NMR ($CDCl_3$, 600 MHz): δ = 8.06 (d, *J* = 8.9 Hz, 2H, arom.), 7.78 (d, *J* = 15.7 Hz, 1H, H-3), 7.67 (br, 1H, arom.), 7.58-7.55 (m, 1H, arom.), 7.57 (d, *J* = 15.7 Hz, 1H, H-2), 7.45-7.39 (m, 2H, arom.), 7.00 (d, *J* = 8.9 Hz, 2H, arom.), 4.53 (s, 2H, CH_2Br), 3.90 (s, 3H, OCH_3) ppm. ^{13}C NMR ($CDCl_3$, 150.9 MHz): δ = 188.59 (CO), 163.55 ($\underline{C}$-OCH_3), 143.15 (C-3), 138.60 (C, arom.), 135.72 (C, arom.), 130.94 (C, arom.), 130.91 (2CH, arom.), 130.85 (CH, arom.), 129.48 (CH, arom.), 128.66 (CH, arom.), 128.48 (CH, arom.), 122.51 (C-2), 113.91 (2CH arom.), 55.55 (OCH_3), 40.83 (CH_2Br) ppm.

(*E*)-1-(4-methoxyphenyl)-3-(3-[(4a*S*,6*R*,8a*S*)-6-hydroxy-3-methoxy-5,6,9,10-tetrahydro-4a*H*-[1]benzofuro[3a,3,2-ef][2]benzazepin-11(12*H*)-ylmethyl]phenyl)prop-2-en-1-one **6b**

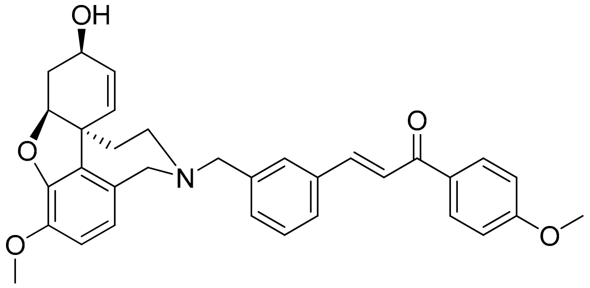

To a solution of norgalanthamine **10** (0.082 g, 0.299 mmol) in anhydrous acetonitrile (9 mL) under argon atmosphere was added bromide **23** (0.090 g, 0.272 mmol) and anhydrous K_2CO_3 (0.113 g, 0.816 mmol). After stirring for 20 h at 60 °C, the mixture was filtered through a pad of Celite. The filtrate was concentrated under reduced pressure and subjected to purification by flash column chromatography on silica gel ($CH_2Cl_2/CH_3OH/NH_4OH$ = 40/1/0.04) to give the desired product in 59% yield. White crystals; m.p. 80-82°C. $[\alpha]^{20}_D$ = -89.8 (c 0.938, $CHCl_3$). ^{1}H NMR ($CDCl_3$, 600 MHz) δ = 8.05 (d, *J* = 8.8 Hz, 2H, arom.), 7.80 (d, *J* = 15.7 Hz, 1H, H-3′), 7.58-7.53 (m, 3H, H-2′, 2arom.), 7.38 (t, *J* = 7.6 Hz, 1H, arom.), 7.34 (d, *J* = 7.6 Hz, 1H, arom.), 6.99 (d, *J* = 8.9 Hz, 2H, arom.), 6.67 (d, *J* = 8.2 Hz, 1H, H-2), 6.49 (d, *J* = 8.2 Hz, 1H, H-1), 6.12 (d, *J* = 10.3 Hz, 1H, H-8), 6.03 (dd, *J* = 10.3, 4.3 Hz, 1H, H-7), 4.69 (br, 1H, H-6), 4.17-4.14 (m, 1H, H-4a), 4.15 (d, *J* = 14.9 Hz, 1H, H-12), 3.90 (s, 3H, OCH_3), 3.85 (s, 3H, OCH_3), 3.72 (s, 2H,

NCH$_2$), 3.71 (d, *J* = 15.0 Hz, 1H, H-12), 3.46 (t, *J* = 13.6 Hz, 1H, H-10), 3.23 (d, *J* = 14.4 Hz, 1H, H-10), 2.73-2.70 (m, 1H, H-5), 2.42 (d, *J* = 9.5 Hz, 1H, OH), 2.16-2.12 (m, 1H, H-9), 2.05-2.01 (m, 1H, H-5), 1.57 (d, *J* = 11.5 Hz, 1H, H-9) ppm. ^{13}C NMR (CDCl$_3$, 150.9 MHz) δ = 188.67 (CO), 163.41 (C arom.), 145.84 (C-3a), 144.13 (C-3), 143.93 (C-3′), 135.10 (2C arom.), 133.30 (C-12b), 131.02 (CH arom.), 130.81 (2CH arom.), 129.13 (C-12a), 128.92 (CH arom.), 128.70 (CH arom.), 127.72 (C-7), 127.46 (CH arom.), 126.80 (C-8), 122.20 (C-1), 121.81 (C-2′), 113.80 (3CH arom.), 111.12 (C-2), 88.75 (C-6), 62.04 (C-4a), 57.14 (C-12), 55.87 (OCH$_3$, NCH$_2$), 55.48 (OCH$_3$), 51.75 (C-10), 48.44 (C-8a), 33.25 (C-9), 29.92 (C-5) ppm. HRMS found for $C_{33}H_{34}NO_5$: m/z 524.2428 [M+H]$^+$, calcd. m/z 524.2431.

3. Synthesis and analytical data of compounds **8b,c,f,g,h**.

1-(3-((*tert*-butyldimethylsilyloxy)methyl)phenyl)ethanol

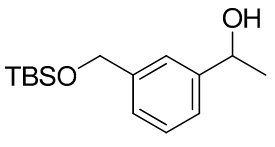

To a stirred under argon atmosphere solution of 3-((*tert*-butyldimethylsilyloxy)methyl)benzaldehyde (0.682 g, 2.72 mmol) in dry THF (12 ml) was added dropwise at 0 °C CH$_3$MgCl (3M in THF, 1.8 ml, 5.45 mmol). The reaction was stirred for 1 h at r.t., cooled down again to 0 °C and quenched with 10 ml sat.aq.NH$_4$Cl. The resulting mixture was extracted three times with ether, and the combined organic extracts were dried over MgSO$_4$, filtered and concentrated under reduced pressure. The residue was purified by flash column chromatography (silica gel, petroleum ether/EtOAc = 10:1) to give the product in 95% yield as colourless oil. ^{1}H NMR (CDCl$_3$, 600 MHz): δ = 7.34-7.33 (m, 1H, arom.), 7.31 (d, *J* = 7.5 Hz, 1H, arom.), 7.26-7.24 (m, 2H, arom.), 4.90 (q, *J* = 6.5 Hz, 1H, CH-OH), 4.75 (s, 2H, -CH$_2$-OSi), 1.50 (d, *J* = 6.5 Hz, CH$_3$), 0.95 (s, 9H, C(CH$_3$)$_3$), 0.10 (s, 6H, Si(CH$_3$)$_2$) ppm. ^{13}C NMR (CDCl$_3$, 150.9 MHz): δ = 145.72 (C arom.), 141.71 (C arom.), 128.42 (CH arom.), 125.21 (CH arom.), 123.95 (CH arom.), 123.03 (CH arom.), 70.45 (CH-OH), 64.90 (-CH$_2$-OSi), 25.94 (CH$_3$), 25.10 (C($\underline{C}$H$_3$)$_3$), 18.43 ($\underline{C}$(CH$_3$)$_3$), -5.25 (Si(CH$_3$)$_2$) ppm.

1-(3-((*tert*-butyldimethylsilyloxy)methyl)phenyl)ethanone **24**

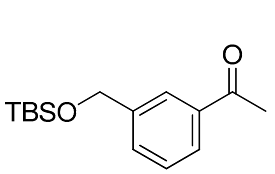

A suspension of 1-(3-((*tert*-butyldimethylsilyloxy)methyl)phenyl)ethanol (0.680 g, 2.55 mmol), PCC (1.1 g, 5.10 mmol) and Celite (1.1 g) in CH$_2$Cl$_2$ (15 ml) was stirred for 20 h at r.t. The mixture was diluted with petroleum ether (30 ml) and filtered through a pad of Celite. The filtrate was concentrated under reduced pressure and subjected to flash column chromatography (silica gel, petroleum ether/EtOAc = 10:1) to give the product in 88% yield as colourless oil. ^{1}H NMR (CDCl$_3$, 600 MHz): δ = 7.92-7.91 (m, 1H, arom.), 7.85 (dd, *J* = 7.7, 0.6 Hz, 1H, arom.), 7.55 (dd, *J* = 7.7, 0.7 Hz, 1H, arom.), 7.44 (t, *J* = 7.7 Hz, 1H, arom.), 4.80 (s, 2H, -CH$_2$-OSi), 2.61 (s, 3H, CH$_3$CO), 0.96 (s, 9H, C(CH$_3$)$_3$), 0.12 (s, 6H, Si(CH$_3$)$_2$) ppm. ^{13}C NMR (CDCl$_3$, 150.9 MHz): δ = 198.26 (CO), 142.00 (C arom.), 137.05 (C arom.), 130.68 (CH arom.), 128.50 (CH arom.), 126.87 (CH arom.), 125.81 (CH arom.), 64.45 (-CH$_2$-OSi), 26.69 (CH$_3$), 25.90 (C($\underline{C}$H$_3$)$_3$), 18.38 ($\underline{C}$(CH$_3$)$_3$), -5.28 (Si(CH$_3$)$_2$) ppm.

1-(3-(hydroxymethyl)phenyl)ethanone **25**

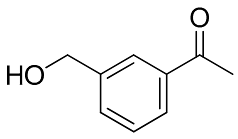

To a solution of silyl ether **24** (0.384 g, 1.44 mmol) in methanol (15 ml) was added 1N HCl (4.5 ml). The mixture was stirred for 30 min at r.t., basified with 10% aq.K_2CO_3 and extracted with ether. The combined organic extracts were dried, filtered, concentrated under reduced pressure and subjected to flash column chromatography (silica gel, petroleum ether/EtOAc = 1:1) to give the product in quantitative yield as colourless oil. ^{1}H NMR ($CDCl_3$, 600 MHz): δ = 7.96-7.95 (m, 1H, arom.), 7.88 (dd, *J* = 7.7, 1.1 Hz, 1H, arom.), 7.58 (dd, *J* = 7.7, 0.7 Hz, 1H, arom.), 7.46 (dt, *J* = 7.7, 2.0 Hz, 1H, arom.), 4.77 (d, *J* = 2.0 Hz, 2H, -$\underline{CH_2}$-OH), 2.61 (d, *J* = 2.5 Hz, 3H, CH_3CO) ppm. ^{13}C NMR ($CDCl_3$, 150.9 MHz): δ = 198.31 (CO), 141.44 (C arom.), 137.32 (C arom.), 131.61 (CH arom.), 128.85 (CH arom.), 127.60 (CH arom.), 126.63 (CH arom.), 64.76 (-CH_2-OH), 26.75 (CH_3) ppm.

General procedure for the synthesis of compounds **26b,c,f,g,h**.

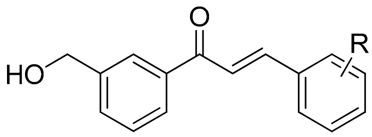

To a solution of 1-(3-(hydroxymethyl)phenyl)ethanone **25** (0.100 g, 0.666 mmol) and appropriate benzaldehyde **12b,c,f,g,h** (0.8 mmol) in ethanol (4 ml) was added $LiOH.H_2O$ (20 mol%, 0.006 g, 0.133 mmol) and the reaction was stirred overnight at r.t. The mixture was concentrated under reduced pressure and directly subjected to flash column chromatography (silica gel, petroleum ether/EtOAc = 10:1) to give the desired product.

(*E*)-1-(3-(hydroxymethyl)phenyl)-3-(4-methoxyphenyl)prop-2-en-1-one **26b**

Yield: 87%; yellow crystals; m.p. 62-65 °C. ^{1}H NMR ($CDCl_3$, 600 MHz): δ = 8.00 (br, 1H, arom.), 7.93 (d, *J* = 7.7 Hz, 1H, arom.), 7.79 (d, *J* = 15.6 Hz, 1H, H-3), 7.61 (d, *J* = 8.8 Hz, 2H, arom.), 7.59 (d, *J* = 8.2 Hz, 1H, arom.), 7.49 (t, *J* = 7.7 Hz, 1H, arom.), 7.42 (d, *J* = 15.6 Hz, 1H, H-2), 6.94 (d, *J* = 8.8 Hz, 2H, arom.), 4.79 (s, 2H, CH_2O), 3.86 (s, 3H, OCH_3) ppm. ^{13}C NMR ($CDCl_3$, 150.9 MHz): δ = 190.46 (CO), 161.70 ($\underline{C}$-OCH3), 144.90 (C-3), 138.72 ($\underline{C}$-CH2OH), 131.04 (CH arom.), 130.29 (2CH arom.), 128.79 (CH arom.), 127.64 (CH arom.), 127.51 (C arom.), 126.73 (CH arom.), 119.60 (C-2), 114.39 (2CH arom.), 64.87 (CH_2O), 55.41 (OCH_3) ppm.

(*E*)-1-(3-(hydroxymethyl)phenyl)-3-(3-methoxyphenyl)prop-2-en-1-one **26c**

Yield: 88%; white crystals; m.p. 60-63 °C. ^{1}H NMR ($CDCl_3$, 600 MHz): δ = 8.01 (d, *J* = 0.5 Hz, 1H, arom.), 7.94 (d, *J* = 7.7 Hz, 1H, arom.), 7.78 (d, *J* = 15.7 Hz, 1H, H-3), 7.60 (dd, *J* = 7.6, 0.6 Hz, 1H, arom.), 7.51 (d, *J* = 15.7 Hz, 1H, H-2), 7.50 (t, *J* = 7.7 Hz, 1H, arom.), 7.34 (t, *J* = 7.8 Hz, 1H, arom.), 7.25 (d, *J* = 7.6 Hz, 1H, arom.), 7.16 (t, *J* = 2.2 Hz, 1H, arom.), 6.97 (ddd, *J* = 8.2, 2.6, 0.8 Hz, 1H, arom.), 4.80 (s, 2H, CH_2O), 3.86 (s, 3H, OCH_3) ppm. ^{13}C NMR ($CDCl_3$, 150.9 MHz): δ = 190.44 (CO), 159.90 ($\underline{C}$-OCH3), 144.95 (C-3), 141.44 (C arom.), 138.39 (C arom.), 136.15 (C arom.), 131.27 (CH arom.), 129.94 (CH arom.), 128.85 (CH arom.), 127.74 (CH arom.), 126.80 (CH arom.), 122.23 (CH arom.), 121.14 (C-2), 116.34 (CH arom.), 113.45 (CH arom.), 64.82 (CH_2O), 55.36 (OCH_3) ppm.

(*E*)-1-(3-(hydroxymethyl)phenyl)-3-p-tolylprop-2-en-1-one **26f**

Yield: 82%; white crystals; m.p. 119-121 ºC. ^{1}H NMR ($CDCl_3$, 600 MHz): δ = 8.01 (d, *J* = 0.5 Hz, 1H, arom.), 7.93 (d, *J* = 7.7 Hz, 1H, arom.), 7.80 (d, *J* = 15.7 Hz, 1H, H-3), 7.59 (dd, *J* = 7.7, 0.6 Hz, 1H, arom.), 7.55 (d, *J* = 8.2 Hz, 2H, arom.), 7.50 (d, *J* = 15.7 Hz, 1H, H-2), 7.49 (t, *J* = 7.7 Hz, 1H, arom.), 7.23 (d, *J* = 8.0 Hz, 2H, arom.), 4.80 (s, 2H, CH_2O), 2.40 (s, 3H, CH_3) ppm. ^{13}C NMR ($CDCl_3$, 150.9 MHz): δ = 190.53 (CO), 145.14 (C-3), 141.42 (C arom.), 141.19 (C arom.), 138.57 (C arom.), 132.04 (C arom.), 131.14 (CH arom.), 129.70 (CH arom.), 128.81 (CH arom.), 128.53 (2CH arom.), 127.70 (CH arom.), 126.77 (CH arom.), 120.91 (C-2), 64.85 (CH_2O), 21.54 (CH_3) ppm.

(*E*)-1-(3-(hydroxymethyl)phenyl)-3-m-tolylprop-2-en-1-one **26g**

Yield: 79%; white crystals; m.p. 58-61 ºC. ^{1}H NMR ($CDCl_3$, 600 MHz): δ = 8.01 (s, 1H, arom.), 7.94 (d, *J* = 7.7 Hz, 1H, arom.), 7.79 (d, *J* = 15.7 Hz, 1H, H-3), 7.60 (d, *J* = 7.6 Hz, 1H, arom.), 7.52 (d, *J* = 15.7 Hz, 1H, H-2), 7.50 (t, *J* = 7.6 Hz, 1H, arom.), 7.45 (d, *J* = 8.8 Hz, 2H, arom.), 7.31 (t, *J* = 7.5 Hz, 1H, arom.), 7.24 (t, *J* = 7.5 Hz, 1H, arom.), 4.80 (s, 2H, CH_2O), 2.40 (s, 3H, CH_3) ppm. ^{13}C NMR ($CDCl_3$, 150.9 MHz): δ = 190.48 (CO), 145.26 (C-3), 141.44 (C arom.), 138.62 (C arom.), 138.47 (C arom.), 134.71 (C arom.), 131.48 (CH arom.), 131.21 (CH arom.), 129.05 (CH arom.), 128.83 (2CH arom.), 127.73 (CH arom.), 126.79 (CH arom.), 125.78 (CH arom.), 121.69 (C-2), 64.84 (CH_2O), 21.33 (CH_3) ppm.

(*E*)-3-(3,4-dimethylphenyl)-1-(3-(hydroxymethyl)phenyl)prop-2-en-1-one **26h**

Yield: 89%; yellow crystals; m.p. 114-116 ºC. ^{1}H NMR ($CDCl_3$, 600 MHz): δ = 8.00 (s, 1H, arom.), 7.93 (d, *J* = 7.7 Hz, 1H, arom.), 7.78 (d, *J* = 15.7 Hz, 1H, H-3), 7.59 (d, *J* = 7.6 Hz, 1H, arom.), 7.49 (d, *J* = 15.7 Hz, 1H, H-2), 7.50 (t, *J* = 7.8 Hz, 1H, arom.), 7.42 (s, 1H, arom.), 7.39 (d, *J* = 7.7 Hz, 1H, arom.), 7.12 (t, *J* = 7.5 Hz, 1H, arom.), 4.79 (s, 2H, CH_2O), 2.31 (s, 3H, CH_3), 2.30 (s, 3H, CH_3) ppm. ^{13}C NMR ($CDCl_3$, 150.9 MHz): δ = 190.56 (CO), 145.38 (C-3), 141.40 (C arom.), 139.99 (C arom.), 138.61 (C arom.), 137.23 (C arom.), 132.43 (C arom.), 131.11 (CH arom.), 130.23 (CH arom.), 129.64 (CH arom.), 128.79 (CH arom.), 127.69 (CH arom.), 126.77 (CH arom.), 126.24 (CH arom.), 120.72 (C-2), 64.86 (CH_2O), 19.89 (CH_3), 19.75 (CH_3) ppm.

General procedure for the synthesis of compounds **27b,c,f,g,h**.

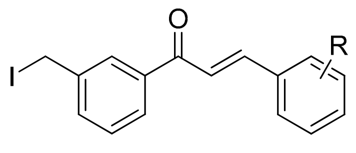

A mixture of PPh_3 (0.220 g, 0.84 mmol), imidazole (0.076 g, 1.12 mmol) and I_2 (0.213 g, 0.84 mmol) in dry THF (7 ml) was stirred under argon atmosphere for 10 min at r.t. A solution of appropriate alcohol **26b,c,f,g,h** (0.56 mmol) in dry THF (5 ml) was added and the mixture was stirred for 1 hour at r.t. The reaction was quenched with aq.$Na_2S_2O_3$ and extracted with ether. The combined organic extracts were washed with brine, dried, filtered, concentrated

under reduced pressure and subjected to flash column chromatography (silica gel, petroleum ether/EtOAc = 10:1) to give the desired product.

(*E*)-1-(3-(iodomethyl)phenyl)-3-(4-methoxyphenyl)prop-2-en-1-one **27b**

Yield: 81%; yellow crystals; m.p. 75-78 ºC. ^{1}H NMR ($CDCl_3$, 600 MHz): δ = 8.00 (t, *J* = 1.7 Hz, 1H, arom.), 7.88 (dt, *J* = 7.7, 1.3 Hz, 1H, arom.), 7.79 (d, *J* = 15.6 Hz, 1H, H-3), 7.62 (d, *J* = 8.7 Hz, 2H, arom.), 7.59 (d, *J* = 8.2 Hz, 1H, arom.), 7.44 (t, *J* = 7.7 Hz, 1H, arom.), 7.38 (d, *J* = 15.6 Hz, 1H, H-2), 6.95 (d, *J* = 8.7 Hz, 2H, arom.), 4.52 (s, 2H, CH_2I), 3.86 (s, 3H, OCH_3) ppm. ^{13}C NMR ($CDCl_3$, 150.9 MHz): δ = 189.96 (CO), 161.76 (C-OCH3), 145.16 (C-3), 139.93 (C arom.), 139.04 (C arom.), 132.79 (CH arom.), 130.32 (2CH arom.), 129.14 (CH arom.), 128.48 (CH arom.), 127.83 (CH arom.), 127.45 (C arom.), 119.47 (C-2), 114.42 (2CH arom.), 55.42 (OCH_3), 4.48 (CH_2I) ppm.

(*E*)-1-(3-(iodomethyl)phenyl)-3-(3-methoxyphenyl)prop-2-en-1-one **27c**

Yield: 77%; yellow crystals; m.p. 61-63 ºC. ^{1}H NMR ($CDCl_3$, 600 MHz): δ = 8.01 (t, *J* = 1.7 Hz, 1H, arom.), 7.88 (dt, *J* = 7.8, 1.5 Hz, 1H, arom.), 7.78 (d, *J* = 15.7 Hz, 1H, H-3), 7.61 (ddd, *J* = 7.7, 1.7, 1.1 Hz, 1H, arom.), 7.47 (d, *J* = 15.7 Hz, 1H, H-2), 7.45 (t, *J* = 7.6 Hz, 1H, arom.), 7.35 (t, *J* = 7.8 Hz, 1H, arom.), 7.27-7.25 (m, 1H, arom.), 7.17 (t, *J* = 2.2 Hz, 1H, arom.), 6.98 (ddd, *J* = 8.2, 2.6, 0.9 Hz, 1H arom.), 4.52 (s, 2H, CH_2I), 3.87 (s, 3H, OCH_3) ppm. ^{13}C NMR ($CDCl_3$, 150.9 MHz): δ = 189.96 (CO), 159.92 (C-OCH3), 145.22 (C-3), 140.06 (C arom.), 138.71 (C arom.), 136.09 (C arom.), 133.05 (CH arom.), 129.98 (CH arom.), 129.22 (CH arom.), 128.56 (CH arom.), 127.93 (CH arom.), 122.12 (CH arom.), 121.14 (C-2), 116.39 (CH arom.), 113.52 (CH arom.), 55.37 (OCH_3), 4.33 (CH_2I) ppm.

(*E*)-1-(3-(iodomethyl)phenyl)-3-*p*-tolylprop-2-en-1-one **27f**

Yield: 82%; yellow crystals; m.p. 99-101 ºC. ^{1}H NMR ($CDCl_3$, 600 MHz): δ = 8.00 (t, *J* = 1.7 Hz, 1H, arom.), 7.88 (dt, *J* = 7.8, 1.5 Hz, 1H, arom.), 7.80 (d, *J* = 15.7 Hz, 1H, H-3), 7.61 (ddd, *J* = 7.7, 1.7, 1.2 Hz, 1H, arom.), 7.56 (d, *J* = 8.1 Hz, 2H, arom.), 7.46 (d, *J* = 15.7 Hz, 1H, H-2), 7.45 (t, *J* = 7.7 Hz, 1H, arom.), 7.24 (d, *J* = 7.9 Hz, 2H, arom.), 4.52 (s, 2H, CH_2I), 2.40 (s, 3H, CH_3) ppm. ^{13}C NMR ($CDCl_3$, 150.9 MHz): δ = 190.02 (CO), 145.40 (C-3), 141.28 (C arom.), 139.98 (C arom.), 138.88 (C arom.), 132.91 (CH arom.), 131.98 (CH arom.), 129.72 (2CH arom.), 129.17 (CH arom.), 128.55 (2CH arom.), 128.52 (CH arom.), 127.88 (CH arom.), 120.78 (C-2), 21.56 (CH_3), 4.42 (CH_2I) ppm.

(*E*)-3-(3,4-dimethylphenyl)-1-(3-(iodomethyl)phenyl)prop-2-en-1-one **27h**

Yield: 77%; yellow crystals; m.p. 113-115 ºC. ^{1}H NMR ($CDCl_3$, 600 MHz): δ = 8.00 (t, *J* = 1.7 Hz, 1H, arom.), 7.89 (dt, *J* = 7.9, 1.3 Hz, 1H, arom.), 7.78 (d, *J* = 15.7 Hz, 1H, H-3), 7.59 (ddd, *J* = 7.7, 1.7, 1.2 Hz, 1H, arom.), 7.45 (d, *J* = 15.7 Hz, 1H, H-2), 7.44 (t, *J* = 7.7 Hz, 1H, arom.), 7.44-7.43 (m, 1H, arom.), 7.40 (dd, *J* = 7.8, 1.6 Hz, 1H, arom.), 7.19 (d, *J* = 7.7 Hz, 1H, arom.), 4.52 (s, 2H, CH_2I), 2.32 (s, 3H, CH_3), 2.31 (s, 3H, CH_3) ppm. ^{13}C NMR ($CDCl_3$, 150.9 MHz): δ

= 190.04 (CO), 145.64 (C-3), 140.09 (C arom.), 139.96 (C arom.), 138.94 (C arom.), 137.26 (C arom.), 132.88 (CH arom.), 132.38 (C arom.), 130.26 (CH arom.), 129.68 (CH arom.), 129.15 (CH arom.), 128.50 (CH arom.), 127.89 (CH arom.), 126.25 (CH arom.), 120.60 (C-2), 19.91 (CH_3), 19.77 (CH_3), 4.45 (CH_2I) ppm.

General procedure for the synthesis of compounds **8b,c,f,g,h**.

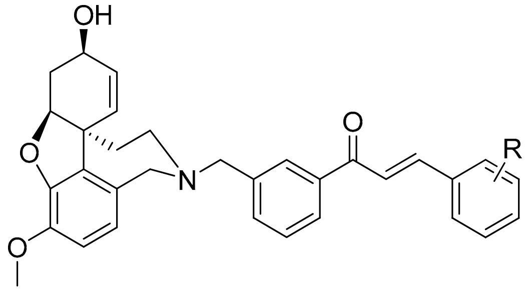

To a solution of norgalanthamine **10** (0.082 g, 0.3 mmol) in anhydrous acetonitrile (10 mL) under argon atmosphere was added appropriate iodide **27b,c,f,g,h** (0.3 mmol) and anhydrous K_2CO_3 (0.124 g, 0.9 mmol). After stirring for 1 h at 60 °C, the mixture was filtered through a pad of Celite. The filtrate was concentrated under reduced pressure and subjected to purification by flash column chromatography on silica gel (CH_2Cl_2/CH_3OH/NH_4OH = 50/1/0.05) to give the desired product.

(*E*)-3-(4-methoxyphenyl)-1-(3-[(4a*S*,6*R*,8a*S*)-6-hydroxy-3-methoxy-5,6,9,10-tetrahydro-4a*H*-[1]benzofuro[3a,3,2-ef][2]benzazepin-11(12*H*)-ylmethyl]phenyl)prop-2-en-1-one **8b**

Yield: 65%; yellow crystals; m.p. 81-84°C. $[\alpha]^{20}_D$ = -100.8 (c 1.064, $CHCl_3$). ^1H NMR ($CDCl_3$, 600 MHz) δ = 7.93 (d, *J* = 8.1 Hz, 2H, arom.), 7.79 (d, *J* = 15.6 Hz, 1H, H-3′), 7.60 (d, *J* = 8.6 Hz, 2H, arom.), 7.53 (d, *J* = 7.4 Hz, 1H, arom.), 7.46 (t, *J* = 7.5 Hz, 1H, arom.), 7.41 (d, *J* = 15.6 Hz, 1H, H-2′), 6.94 (d, *J* = 8.7 Hz, 2H, arom.), 6.66 (d, *J* = 8.1 Hz, 1H, H-2), 6.49 (d, *J* = 8.1 Hz, 1H, H-1), 6.12 (d, *J* = 10.3 Hz, 1H, H-8), 6.02 (dd, *J* = 10.3, 5.0 Hz, 1H, H-7), 4.68 (br, 1H, H-6), 4.17-4.14 (m, 1H, H-4a), 4.15 (d, *J* = 15.4 Hz, 1H, H-12), 3.86 (s, 3H, OCH_3), 3.84 (s, 3H, OCH_3), 3.76 (s, 2H, NCH_2), 3.71 (d, *J* = 15.4 Hz, 1H, H-12), 3.45 (t, *J* = 13.5 Hz, 1H, H-10), 3.22 (d, *J* = 14.5 Hz, 1H, H-10), 2.73-2.70 (m, 1H, H-5), 2.42 (br, 1H, OH), 2.14 (t, *J* = 13.3 Hz, 1H, H-9), 2.04-2.00 (m, 1H, H-5), 1.56 (d, *J* = 13.5 Hz, 1H, H-9) ppm. ^{13}C NMR ($CDCl_3$, 150.9 MHz) δ = 190.45 (CO), 161.65 (C arom.), 145.84 (C-3a), 144.61 (C-3′), 144.13 (C-3), 138.54 (2C arom.), 133.29 (C-12b), 133.19 (CH arom.), 130.24 (2CH arom., C-12a), 128.29 (C arom.), 128.62 (CH arom.), 127.70 (C-7), 127.58 (CH arom.), 127.33 (CH arom.), 126.82 (C-8), 122.18 (C-1), 119.72 (C-2′), 114.37 (2CH arom.), 111.13 (C-2), 88.75 (C-6), 62.05 (C-4a), 57.19 (C-12), 55.86 (OCH_3, NCH_2), 55.40 (OCH_3), 51.79 (C-10), 48.44 (C-8a), 33.33 (C-9), 29.92 (C-5) ppm. HRMS found for $C_{33}H_{34}NO_5$: m/z 524.2428 $[M+H]^+$, calcd. m/z 524.2431.

(*E*)-3-(3-methoxyphenyl)-1-(3-[(4a*S*,6*R*,8a*S*)-6-hydroxy-3-methoxy-5,6,9,10-tetrahydro-4a*H*-[1]benzofuro[3a,3,2-ef][2]benzazepin-11(12*H*)-ylmethyl]phenyl)prop-2-en-1-one **8c**

Yield: 50%; yellow crystals; m.p. 66-69°C. $[\alpha]^{20}_D$ = -93.0 (c 1.099, $CHCl_3$). ^1H NMR ($CDCl_3$, 600 MHz) δ = 7.94 (d, *J* = 7.3 Hz, 2H, arom.), 7.78 (d, *J* = 15.7 Hz, 1H, H-3′), 7.55-7.46 (m, 3H, H-2′, 2 arom.), 7.35 (t, *J* = 7.9 Hz, 1H, arom.), 7.24 (d, *J* = 7.6 Hz, 1H, arom.), 7.17 (s, 1H, arom.), 6.98 (dd, *J* = 8.2, 2.3 Hz, 1H, arom.), 6.66 (d, *J* = 8.2 Hz, 1H, H-2), 6.49 (d, *J* = 8.2 Hz, 1H, H-1),

6.12 (d, *J* = 10.4 Hz, 1H, H-8), 6.02 (dd, *J* = 10.2, 5.0 Hz, 1H, H-7), 4.68 (br, 1H, H-6), 4.17-4.15 (m, 1H, H-12), 4.15 (s, 1H, H-4a), 3.86 (s, 3H, OCH_3), 3.84 (s, 3H, OCH_3), 3.77 (s, 2H, NCH_2), 3.73-3.70 (m, 1H, H-12), 3.48-3.44 (m, 1H, H-10), 3.22 (d, *J* = 14.5 Hz, 1H, H-10), 2.73-2.70 (m, 1H, H-5), 2.42 (br, 1H, OH), 2.14 (t, *J* = 12.3 Hz, 1H, H-9), 2.04-2.00 (m, 1H, H-5), 1.57 (d, *J* = 13.6 Hz, 1H, H-9) ppm. ^{13}C NMR ($CDCl_3$, 150.9 MHz) δ = 190.42 (CO), 159.89 (C arom.), 145.86 (C-3a), 144.68 (C-3′), 144.18 (C-3), 138.23 (C arom.), 136.23 (2C arom.), 133.47 (C-12b), 133.27 (CH arom.), 129.92 (CH arom., C-12a), 129.00 (CH arom.), 128.70 (2CH arom.), 127.74 (C-7), 126.81 (C-8), 122.36 (C-1, C-2′), 121.08 (CH arom.), 116.07 (CH arom.), 113.61 (CH arom.), 111.12 (C-2), 88.73 (C-6), 62.03 (C-4a), 57.11 (C-12), 55.84 (OCH_3, NCH_2), 55.35 (OCH_3), 51.78 (C-10), 48.42 (C-8a), 33.28 (C-9), 29.91 (C-5) ppm. HRMS found for $C_{33}H_{34}NO_5$: m/z 524.2428 $[M+H]^+$, calcd. m/z 524.2431.

(*E*)-1-(3-[(4a*S*,6*R*,8a*S*)-6-hydroxy-3-methoxy-5,6,9,10-tetrahydro-4a*H*-[1]benzofuro[3a,3,2-ef][2]benzazepin-11(12*H*)-ylmethyl]phenyl)-3-p-tolylprop-2-en-1-one **8f**

Yield: 84%; yellow crystals; m.p. 76-79°C. $[\alpha]^{20}_D$ = -105.2 (c 1.035, $CHCl_3$). ^{1}H NMR ($CDCl_3$, 600 MHz) δ = 7.93 (d, *J* = 8.4 Hz, 2H, arom.), 7.80 (d, *J* = 15.7 Hz, 1H, H-3′), 7.55-7.53 (m, 3H, arom.), 7.49 (d, *J* = 15.7 Hz, 1H, H-2′), 7.46 (t, *J* = 7.5 Hz, 1H, arom.), 7.23 (d, *J* = 7.8 Hz, 2H, arom.), 6.65 (d, *J* = 8.1 Hz, 1H, H-2), 6.49 (d, *J* = 8.1 Hz, 1H, H-1), 6.12 (d, *J* = 10.3 Hz, 1H, H-8), 6.02 (dd, *J* = 10.3, 5.0 Hz, 1H, H-7), 4.68 (br, 1H, H-6), 4.15 (d, *J* = 15.6 Hz, 1H, H-12), 4.15-4.13 (m, 1H, H-4a), 3.86 (s, 3H, OCH_3), 3.76 (s, 2H, NCH_2), 3.71 (d, *J* = 15.5 Hz, 1H, H-12), 3.45 (t, *J* = 13.5 Hz, 1H, H-10), 3.22 (d, *J* = 14.0 Hz, 1H, H-10), 2.71 (d, *J* = 15.5 Hz, 1H, H-5), 2.42 (d, *J* = 11.5 Hz, 1H, OH), 2.40 (s, 3H, CH_3), 2.14 (t, *J* = 12.4 Hz, 1H, H-9), 2.04-2.01 (m, 1H, H-5), 1.56 (d, *J* = 13.4 Hz, 1H, H-9) ppm. ^{13}C NMR ($CDCl_3$, 150.9 MHz) δ = 190.53 (CO), 145.84 (C-3a), 144.85 (C-3′), 144.14 (C-3), 141.10 (2C arom.), 138.39 (C arom.), 133.30 (CH arom.), 132.11 (C arom., C-12b), 129.67 (2CH arom., C-12a), 128.95 (CH arom.), 128.65 (CH arom.), 128.49 (2CH arom.), 127.70 (C-7), 127.38 (CH arom.), 126.80 (C-8), 122.19 (C-1), 121.05 (C-2′), 111.12 (C-2), 88.75 (C-6), 62.05 (C-4a), 57.14 (C-12), 55.84 (OCH_3, NCH_2), 51.81 (C-10), 48.44 (C-8a), 33.34 (C-9), 29.92 (C-5), 21.54 (CH_3) ppm. HRMS found for $C_{33}H_{34}NO_4$: m/z 508.2481 $[M+H]^+$, calcd. m/z 508.2482.

(*E*)-1-(3-[(4a*S*,6*R*,8a*S*)-6-hydroxy-3-methoxy-5,6,9,10-tetrahydro-4a*H*-[1]benzofuro[3a,3,2-ef][2]benzazepin-11(12*H*)-ylmethyl]phenyl)-3-m-tolylprop-2-en-1-one **8g**

Yield: 60%; yellow crystals; m.p. 67-70°C. $[\alpha]^{20}_D$ = -84.2 (c 1.002, $CHCl_3$). ^{1}H NMR ($CDCl_3$, 600 MHz) δ = 7.94 (d, *J* = 8.0 Hz, 2H, arom.), 7.79 (d, *J* = 15.7 Hz, 1H, H-3′), 7.55-7.45 (m, 5H, 4 arom., H-2′), 7.32 (t, *J* = 7.6 Hz, 1H, arom.), 7.24 (d, *J* = 7.4 Hz, 1H, arom.), 6.65 (d, *J* = 8.2 Hz, 1H, H-2), 6.49 (d, *J* = 8.2 Hz, 1H, H-1), 6.12 (d, *J* = 10.3 Hz, 1H, H-8), 6.02 (dd, *J* = 10.1, 5.0 Hz, 1H, H-7), 4.68 (br, 1H, H-6), 4.17-4.14 (m, 1H, H-4a), 4.16 (d, *J* = 15.0 Hz, 1H, H-12), 3.83 (s, 3H, OCH_3), 3.76 (s, 2H, NCH_2), 3.71 (d, *J* = 15.5 Hz, 1H, H-12), 3.45 (t, *J* = 13.6 Hz, 1H, H-10), 3.22 (d, *J* = 13.8 Hz, 1H, H-10), 2.71 (d, *J* = 15.7 Hz, 1H, H-5), 2.41 (m, 4H, OH, CH_3), 2.14 (t, *J* = 12.4 Hz, 1H, H-9), 2.04-2.00 (m, 1H, H-5), 1.56 (d, *J* = 13.3 Hz, 1H, H-9) ppm. ^{13}C NMR ($CDCl_3$, 150.9 MHz) δ = 190.48 (CO), 145.85 (C-3a), 145.00 (C-3′),144.15 (C-3), 138.62 (C arom.), 138.31 (C arom.), 134.80 (C arom.), 133.39 (C arom.), 133.27 (C-12b), 131.41 (CH arom.), 129.13 (CH arom.), 128.98 (C-12a), 128.81 (3CH arom.), 128.66 (CH arom.), 127.72 (C-

7), 127.44 (CH arom.), 126.76 (C-8), 125.63 (CH arom.), 122.19 (C-1), 121.85 (C-2′), 111.13 (C-2), 88.74 (C-6), 62.04 (C-4a), 57.18 (C-12), 55.85 (OCH_3, NCH_2), 51.76 (C-10), 48.44 (C-8a), 33.31 (C-9), 29.91 (C-5), 21.33 (CH_3) ppm. HRMS found for $C_{33}H_{34}NO_4$: m/z 508.2481 $[M+H]^+$, calcd. m/z 508.2482.

(*E*)-3-(3,4-dimethylphenyl)-1-(3-[(4a*S*,6*R*,8a*S*)-6-hydroxy-3-methoxy-5,6,9,10-tetrahydro-4a*H*-[1]benzofuro[3a,3,2-ef][2]benzazepin-11(12*H*)-ylmethyl]phenyl)prop-2-en-1-one **8h**

Yield: 67%; yellow crystals; m.p. 76-80°C. $[\alpha]^{20}_{D}$ = -98.0 (c 1.116, $CHCl_3$). ^1H NMR ($CDCl_3$, 600 MHz) δ = 7.93 (d, *J* = 8.6 Hz, 2H, arom.), 7.78 (d, *J* = 15.7 Hz, 1H, H-3′), 7.54 (d, *J* = 7.5 Hz, 1H, arom.), 7.48 (d, *J* = 15.7 Hz, 1H, H-2′), 7.46 (t, *J* = 7.5 Hz, 1H, arom.), 7.42 (s, 1H, arom.), 7.39 (d, *J* = 7.7 Hz, 1H, arom.), 7.19 (d, *J* = 7.7 Hz, 1H, arom.), 6.65 (d, *J* = 8.2 Hz, 1H, H-2), 6.50 (d, *J* = 8.2 Hz, 1H, H-1), 6.12 (d, *J* = 10.2 Hz, 1H, H-8), 6.02 (dd, *J* = 10.2, 5.0 Hz, 1H, H-7), 4.68 (br, 1H, H-6), 4.17-4.14 (m, 1H, H-4a), 4.15 (d, *J* = 15.1 Hz, 1H, H-12), 3.83 (s, 3H, OCH_3), 3.76 (s, 2H, NCH_2), 3.71 (d, *J* = 15.6 Hz, 1H, H-12), 3.45 (t, *J* = 13.6 Hz, 1H, H-10), 3.22 (d, *J* = 14.3 Hz, 1H, H-10), 2.73-2.69 (m, 1H, H-5), 2.42 (d, *J* = 10.9 Hz, 1H, OH), 2.31 (s, 3H, CH_3), 2.30 (s, 3H, CH_3), 2.16-2.12 (m, 1H, H-9), 2.04-2.00 (m, 1H, H-5), 1.56 (d, *J* = 13.4 Hz, 1H, H-9) ppm. ^{13}C NMR ($CDCl_3$, 150.9 MHz) δ = 190.57 (CO), 145.83 (C-3a), 145.11 (C-3′),144.14 (C-3), 139.90 (C arom.), 138.45 (C arom.), 137.21 (2C arom.), 133.27 (CH arom.), 132.51 (C arom., C-12b), 130.21 (CH arom.), 129.70 (CH arom., C-12a), 128.93 (CH arom.), 128.62 (CH arom.), 127.70 (C-7), 127.39 (CH arom.), 126.79 (C-8), 126.11 (CH arom.), 121.21 (C-1), 120.89 (C-2′), 111.13 (C-2), 88.74 (C-6), 62.04 (C-4a), 57.20 (C-12), 55.84 (OCH_3, NCH_2), 51.74 (C-10), 48.44 (C-8a), 33.31 (C-9), 29.91 (C-5), 19.88 (CH_3), 19.76 (CH_3) ppm. HRMS found for $C_{34}H_{36}NO_4$: m/z 522.2637 $[M+H]^+$, calcd. m/z 522.2639.

4. Copies of ^{1}H and ^{13}C NMR spectra of the target compounds.

Compound 4a:

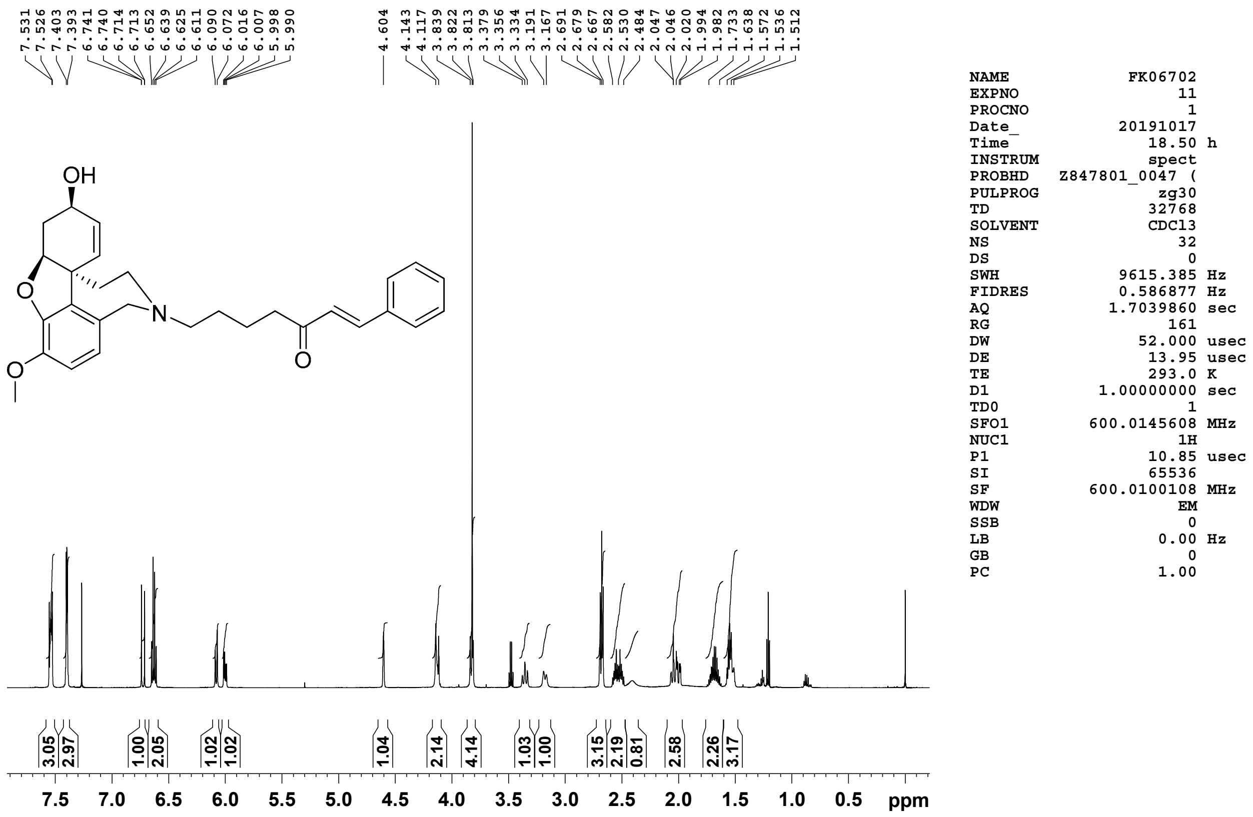

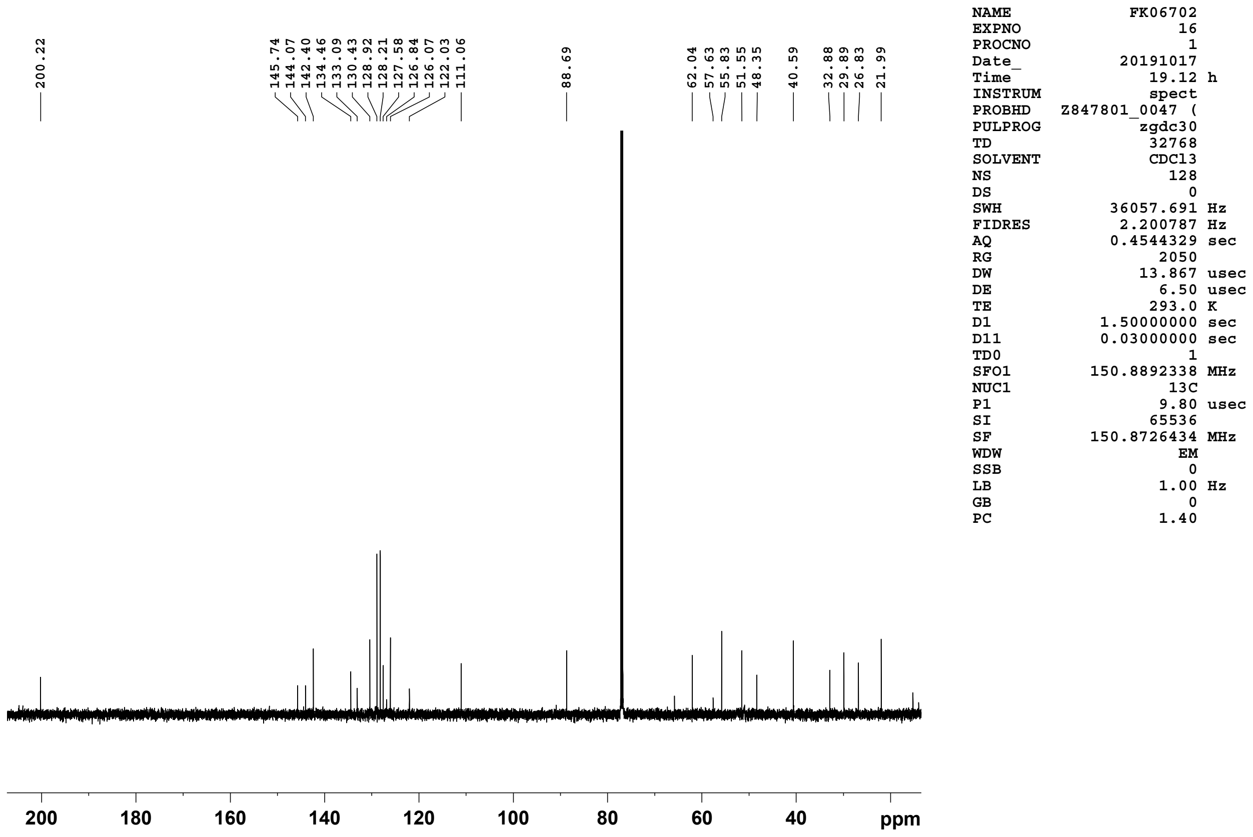

Compound 4b:

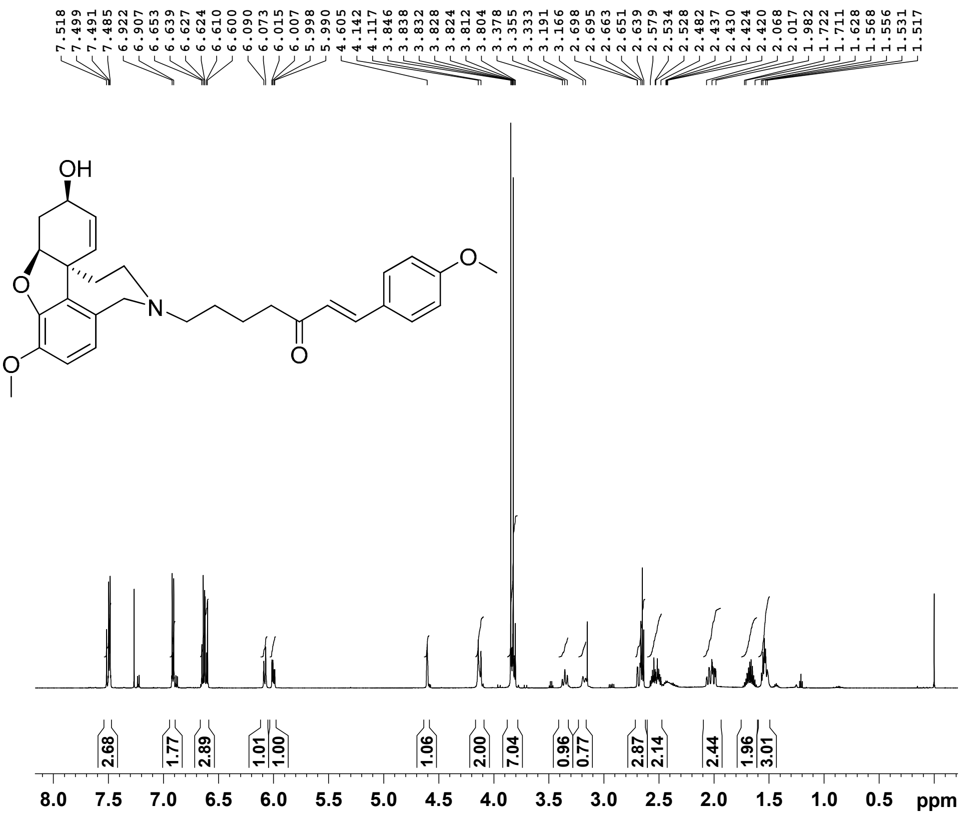

NAME	FK07202	
EXPNO	11	
PROCNO	1	
Date_	20191017	
Time	19.39	h
INSTRUM	spect	
PROBHD	Z847801_0047 (	
PULPROG	zg30	
TD	32768	
SOLVENT	CDCl3	
NS	32	
DS	0	
SWH	9615.385	Hz
FIDRES	0.586877	Hz
AQ	1.7039860	sec
RG	161	
DW	52.000	usec
DE	13.95	usec
TE	293.0	K
D1	1.00000000	sec
TD0	1	
SFO1	600.0145608	MHz
NUC1	1H	
P1	10.85	usec
SI	65536	
SF	600.0100100	MHz
WDW	EM	
SSB	0	
LB	0.00	Hz
GB	0	
PC	1.00	

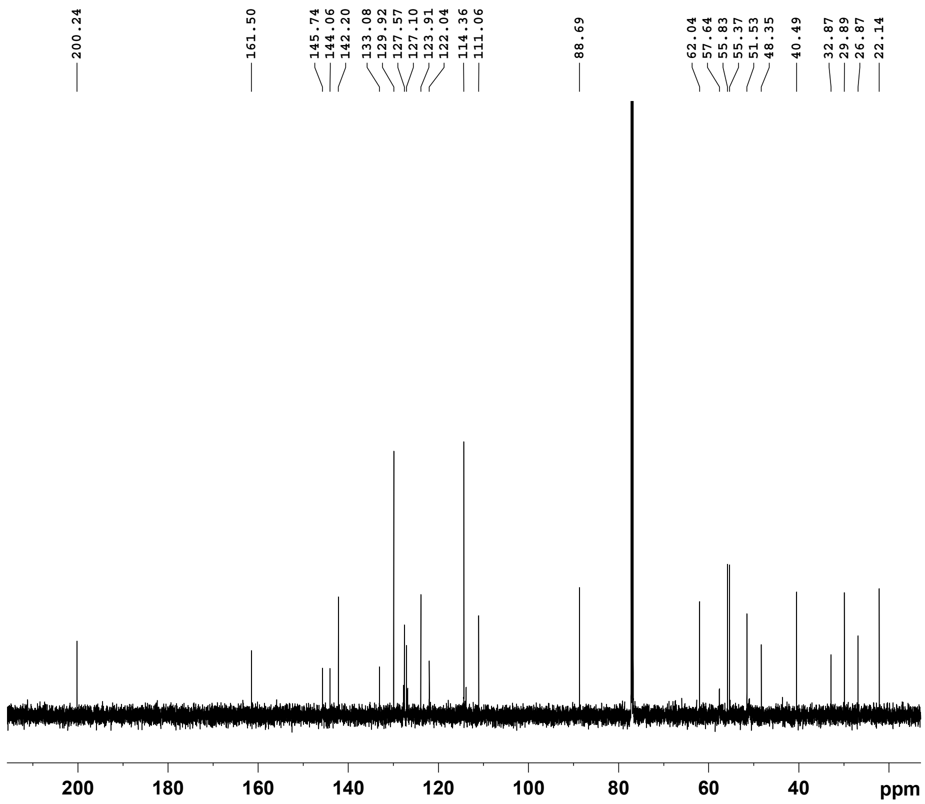

NAME	FK07202	
EXPNO	16	
PROCNO	1	
Date_	20191017	
Time	20.01	h
INSTRUM	spect	
PROBHD	Z847801_0047 (	
PULPROG	zgdc30	
TD	32768	
SOLVENT	CDCl3	
NS	128	
DS	0	
SWH	36057.691	Hz
FIDRES	2.200787	Hz
AQ	0.4544329	sec
RG	2050	
DW	13.867	usec
DE	6.50	usec
TE	293.0	K
D1	1.50000000	sec
D11	0.03000000	sec
TD0	1	
SFO1	150.8892338	MHz
NUC1	13C	
P1	9.80	usec
SI	65536	
SF	150.8726435	MHz
WDW	EM	
SSB	0	
LB	1.00	Hz
GB	0	
PC	1.40	

Compound 4c:

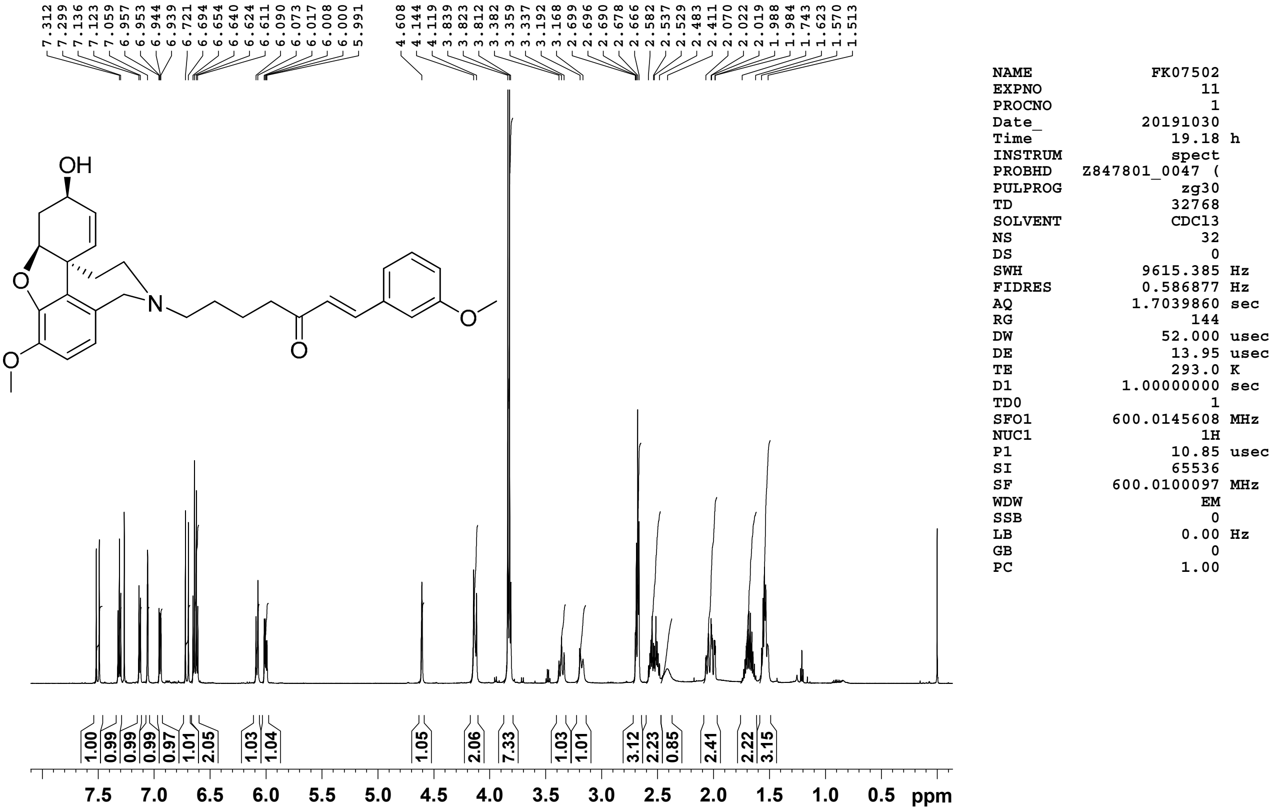
7.312 7.299 7.136 7.123 7.059 6.957 6.953 6.944 6.939 6.721 6.694 6.654 6.640 6.624 6.611 6.090 6.073 6.017 6.008 6.000 5.991
4.608 4.144 4.119 3.839 3.823 3.812 3.382 3.359 3.337 3.192 3.168 2.699 2.696 2.690 2.678 2.666 2.582 2.537 2.529 2.483 2.411 2.070 2.022 2.019 1.988 1.984 1.743 1.623 1.570 1.513
OH
O
N
O
O
O
1.00 0.99 0.99 0.99 0.97 1.01 2.05 1.03 1.04 1.05 2.06 7.33 1.03 1.01 3.12 2.23 0.85 2.41 2.22 3.15
7.5 7.0 6.5 6.0 5.5 5.0 4.5 4.0 3.5 3.0 2.5 2.0 1.5 1.0 0.5 ppm
NAME FK07502
EXPNO 11
PROCNO 1
Date_ 20191030
Time 19.18 h
INSTRUM spect
PROBHD Z847801_0047 (
PULPROG zg30
TD 32768
SOLVENT CDCl3
NS 32
DS 0
SWH 9615.385 Hz
FIDRES 0.586877 Hz
AQ 1.7039860 sec
RG 144
DW 52.000 usec
DE 13.95 usec
TE 293.0 K
D1 1.00000000 sec
TD0 1
SFO1 600.0145608 MHz
NUC1 1H
P1 10.85 usec
SI 65536
SF 600.0100097 MHz
WDW EM
SSB 0
LB 0.00 Hz
GB 0
PC 1.00

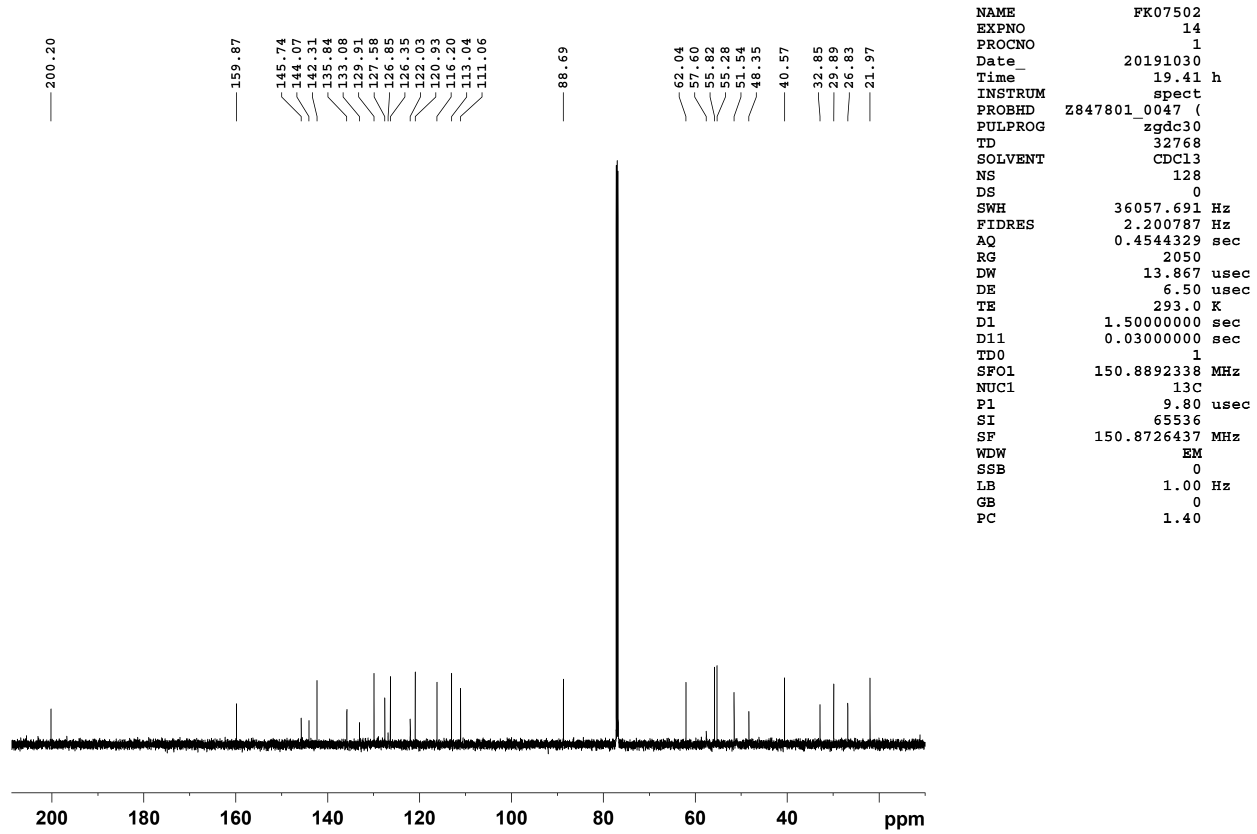
200.20
159.87
145.74 144.07 142.31 135.84 133.08 129.91 127.58 126.85 126.35 122.03 120.93 116.20 113.04 111.06
88.69
62.04 57.60 55.82 55.28 51.54 48.35
40.57
32.85 29.89 26.83 21.97
200 180 160 140 120 100 80 60 40 ppm
NAME FK07502
EXPNO 14
PROCNO 1
Date_ 20191030
Time 19.41 h
INSTRUM spect
PROBHD Z847801_0047 (
PULPROG zgdc30
TD 32768
SOLVENT CDCl3
NS 128
DS 0
SWH 36057.691 Hz
FIDRES 2.200787 Hz
AQ 0.4544329 sec
RG 2050
DW 13.867 usec
DE 6.50 usec
TE 293.0 K
D1 1.50000000 sec
D11 0.03000000 sec
TD0 1
SFO1 150.8892338 MHz
NUC1 13C
P1 9.80 usec
SI 65536
SF 150.8726437 MHz
WDW EM
SSB 0
LB 1.00 Hz
GB 0
PC 1.40

Compound 4e:

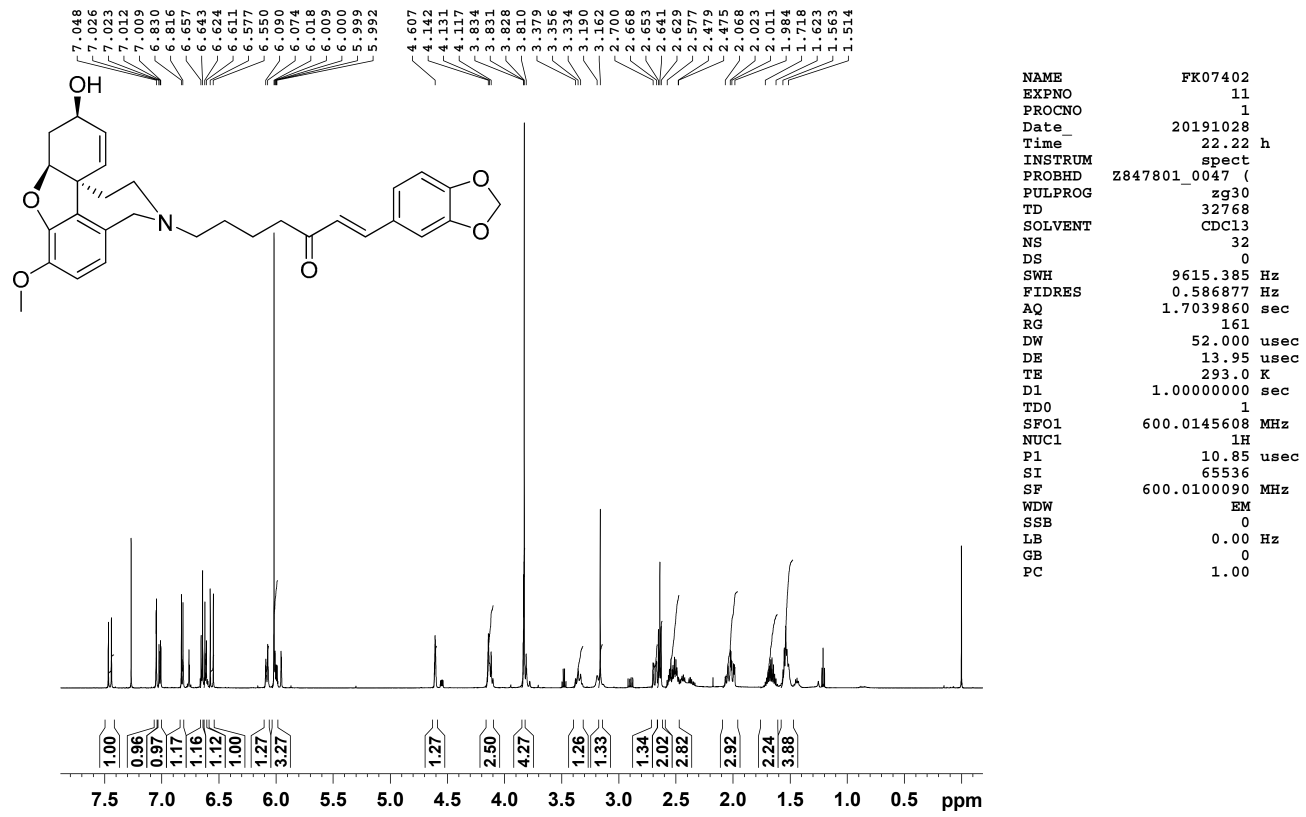
7.048
7.026
7.023
7.012
7.009
6.830
6.816
6.657
6.643
6.624
6.611
6.577
6.550
6.090
6.074
6.018
6.009
6.000
5.999
5.992
4.607
4.142
4.131
4.117
3.834
3.831
3.828
3.810
3.379
3.356
3.334
3.190
3.162
2.700
2.668
2.653
2.641
2.629
2.577
2.479
2.475
2.068
2.023
2.011
1.984
1.718
1.623
1.563
1.514
OH
O
N
O
O
O
O
1.00
0.96
0.97
1.17
1.16
1.12
1.00
1.27
3.27
1.27
2.50
4.27
1.26
1.33
1.34
2.02
2.82
2.92
2.24
3.88
7.5 7.0 6.5 6.0 5.5 5.0 4.5 4.0 3.5 3.0 2.5 2.0 1.5 1.0 0.5 ppm
NAME FK07402
EXPNO 11
PROCNO 1
Date_ 20191028
Time 22.22 h
INSTRUM spect
PROBHD Z847801_0047 (
PULPROG zg30
TD 32768
SOLVENT CDCl3
NS 32
DS 0
SWH 9615.385 Hz
FIDRES 0.586877 Hz
AQ 1.7039860 sec
RG 161
DW 52.000 usec
DE 13.95 usec
TE 293.0 K
D1 1.00000000 sec
TD0 1
SFO1 600.0145608 MHz
NUC1 1H
P1 10.85 usec
SI 65536
SF 600.0100090 MHz
WDW EM
SSB 0
LB 0.00 Hz
GB 0
PC 1.00

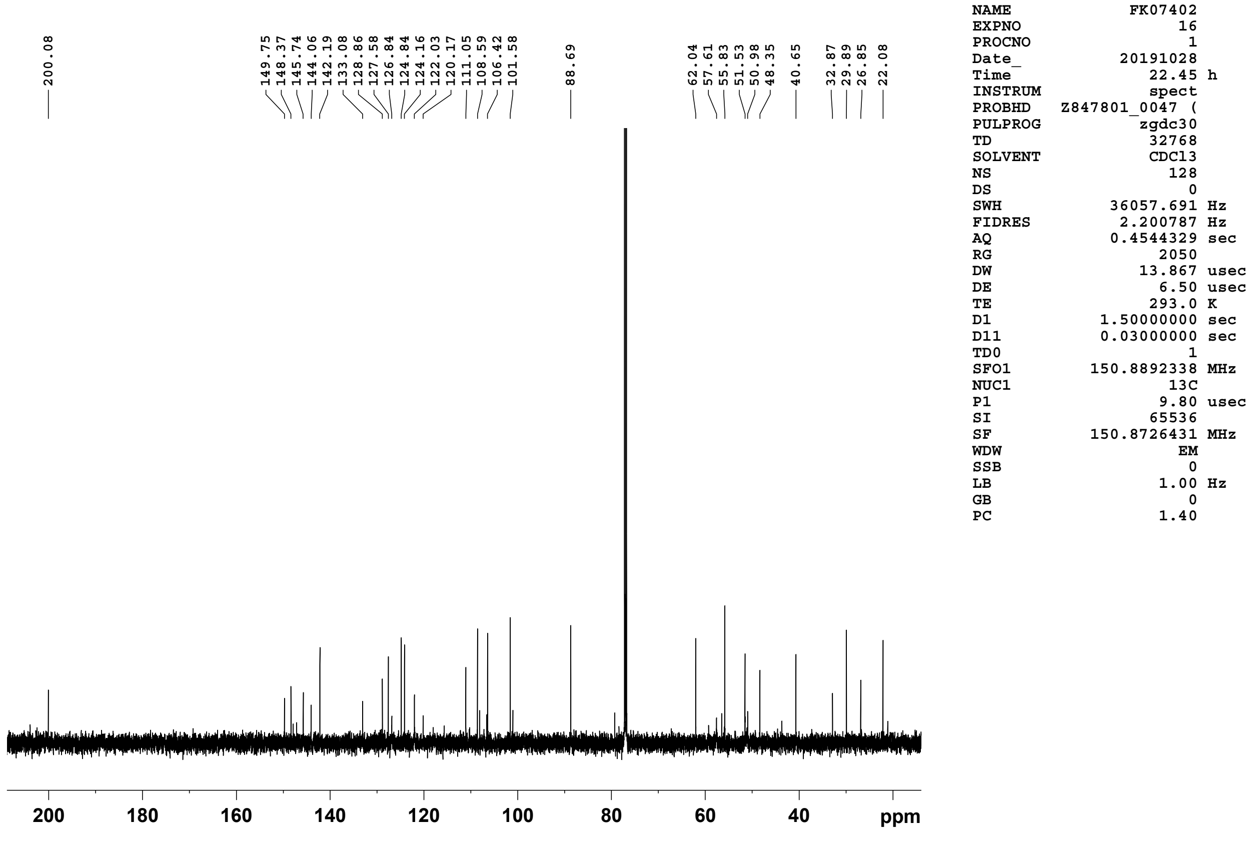
200.08
149.75
148.37
145.74
144.06
142.19
133.08
128.86
127.58
126.84
124.84
124.16
122.03
120.17
111.05
108.59
106.42
101.58
88.69
62.04
57.61
55.83
51.53
50.98
48.35
40.65
32.87
29.89
26.85
22.08
200 180 160 140 120 100 80 60 40 ppm
NAME FK07402
EXPNO 16
PROCNO 1
Date_ 20191028
Time 22.45 h
INSTRUM spect
PROBHD Z847801_0047 (
PULPROG zgdc30
TD 32768
SOLVENT CDCl3
NS 128
DS 0
SWH 36057.691 Hz
FIDRES 2.200787 Hz
AQ 0.4544329 sec
RG 2050
DW 13.867 usec
DE 6.50 usec
TE 293.0 K
D1 1.50000000 sec
D11 0.03000000 sec
TD0 1
SFO1 150.8892338 MHz
NUC1 13C
P1 9.80 usec
SI 65536
SF 150.8726431 MHz
WDW EM
SSB 0
LB 1.00 Hz
GB 0
PC 1.40

Compound 4f:

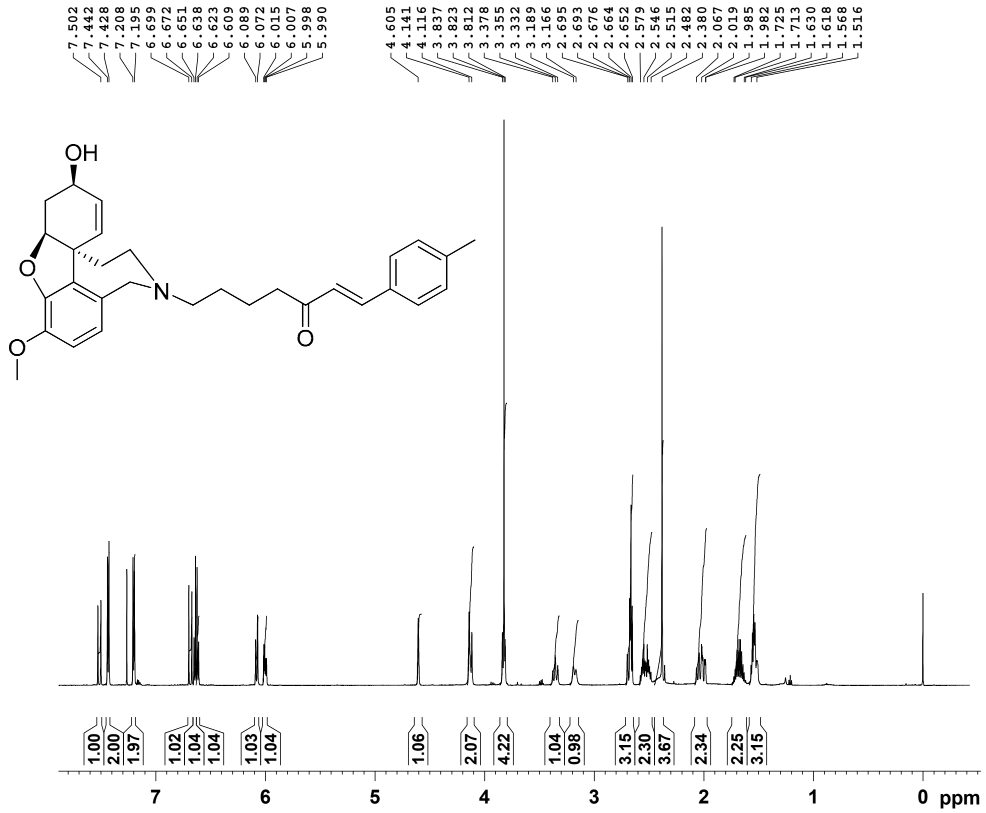

NAME	GC1432	
EXPNO	11	
PROCNO	1	
Date_	20200130	
Time	21.23	h
INSTRUM	spect	
PROBHD	Z847801_0047 (	
PULPROG	zg30	
TD	32768	
SOLVENT	CDCl3	
NS	32	
DS	0	
SWH	9615.385	Hz
FIDRES	0.586877	Hz
AQ	1.7039860	sec
RG	161	
DW	52.000	usec
DE	13.95	usec
TE	293.0	K
D1	1.00000000	sec
TD0	1	
SFO1	600.0145608	MHz
NUC1	1H	
P1	10.85	usec
SI	65536	
SF	600.0100112	MHz
WDW	EM	
SSB	0	
LB	0.00	Hz
GB	0	
PC	1.00	

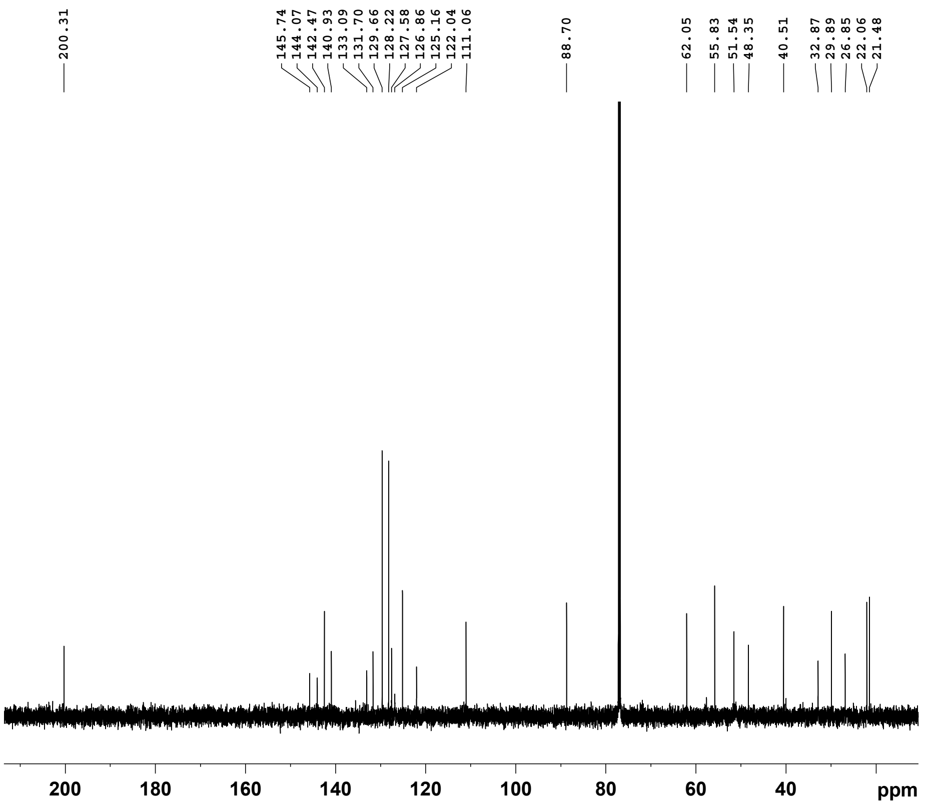

NAME	GC1432	
EXPNO	12	
PROCNO	1	
Date_	20200130	
Time	21.28	h
INSTRUM	spect	
PROBHD	Z847801_0047 (	
PULPROG	zgdc30	
TD	32768	
SOLVENT	CDCl3	
NS	128	
DS	0	
SWH	36057.691	Hz
FIDRES	2.200787	Hz
AQ	0.4544329	sec
RG	2050	
DW	13.867	usec
DE	6.50	usec
TE	293.1	K
D1	1.50000000	sec
D11	0.03000000	sec
TD0	1	
SFO1	150.8892338	MHz
NUC1	13C	
P1	9.80	usec
SI	65536	
SF	150.8726431	MHz
WDW	EM	
SSB	0	
LB	1.00	Hz
GB	0	
PC	1.40	

Compound 4g:

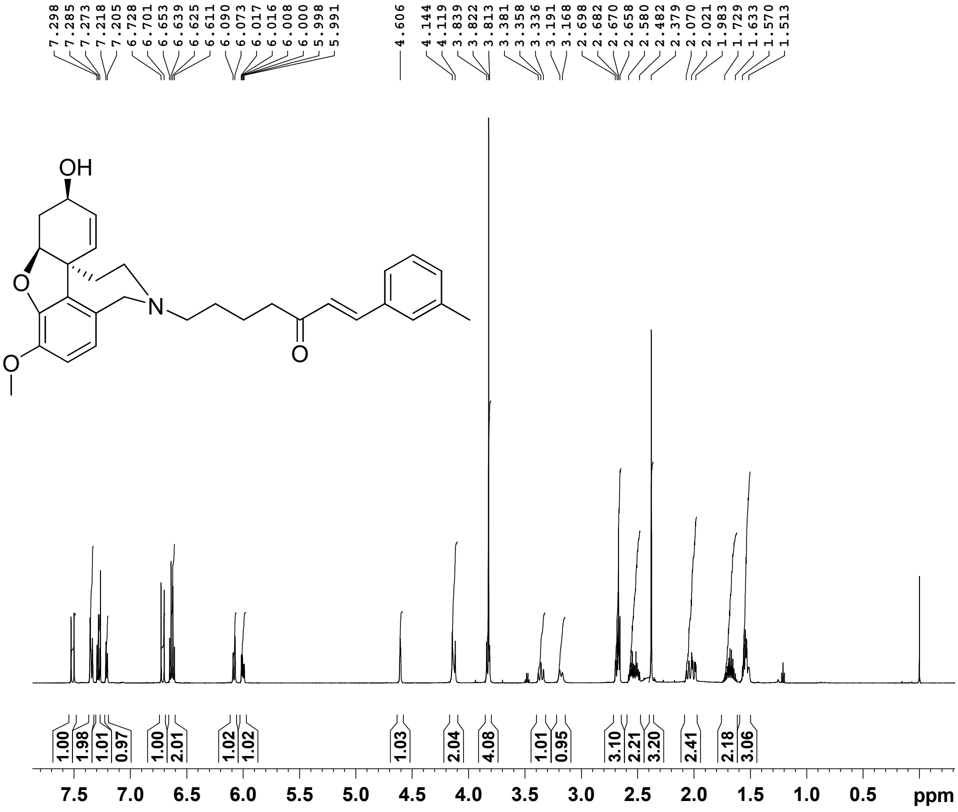

NAME	FK07702
EXPNO	11
PROCNO	1
Date_	20191106
Time	0.01 h
INSTRUM	spect
PROBHD	Z847801_0047 (
PULPROG	zg30
TD	32768
SOLVENT	CDCl3
NS	32
DS	0
SWH	9615.385 Hz
FIDRES	0.586877 Hz
AQ	1.7039860 sec
RG	144
DW	52.000 usec
DE	13.95 usec
TE	293.0 K
D1	1.00000000 sec
TD0	1
SFO1	600.0145608 MHz
NUC1	1H
P1	10.85 usec
SI	65536
SF	600.0100104 MHz
WDW	EM
SSB	0
LB	0.00 Hz
GB	0
PC	1.00

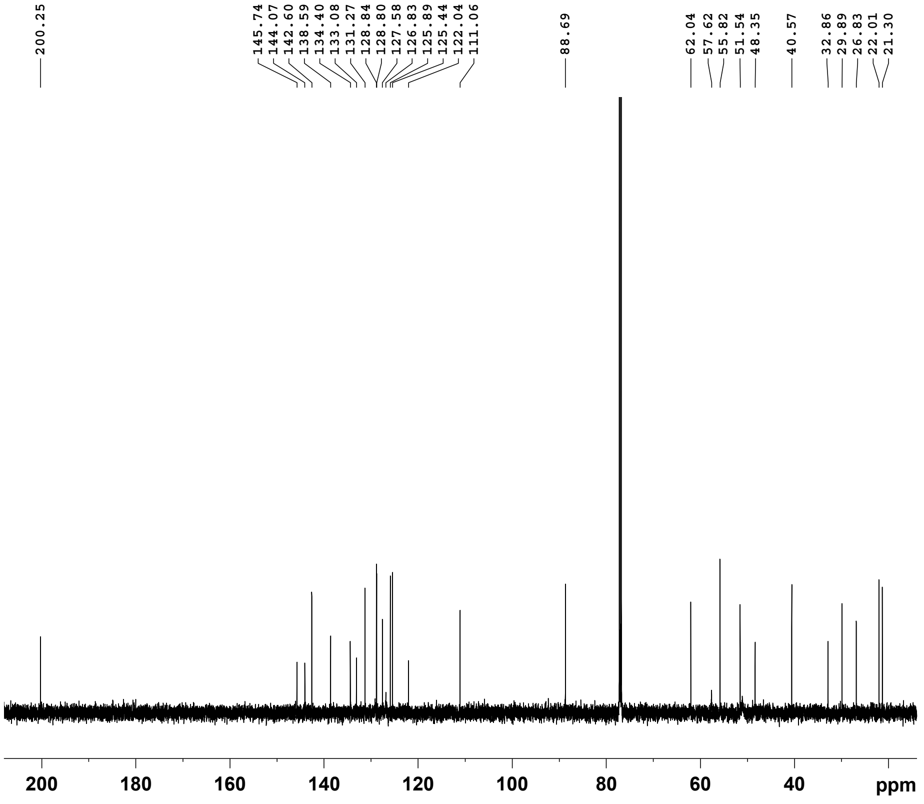

NAME	FK07702
EXPNO	16
PROCNO	1
Date_	20191106
Time	0.24 h
INSTRUM	spect
PROBHD	Z847801_0047 (
PULPROG	zgdc30
TD	32768
SOLVENT	CDCl3
NS	128
DS	0
SWH	36057.691 Hz
FIDRES	2.200787 Hz
AQ	0.4544329 sec
RG	2050
DW	13.867 usec
DE	6.50 usec
TE	293.0 K
D1	1.50000000 sec
D11	0.03000000 sec
TD0	1
SFO1	150.8892338 MHz
NUC1	13C
P1	9.80 usec
SI	65536
SF	150.8726435 MHz
WDW	EM
SSB	0
LB	1.00 Hz
GB	0
PC	1.40

Compound 4h:

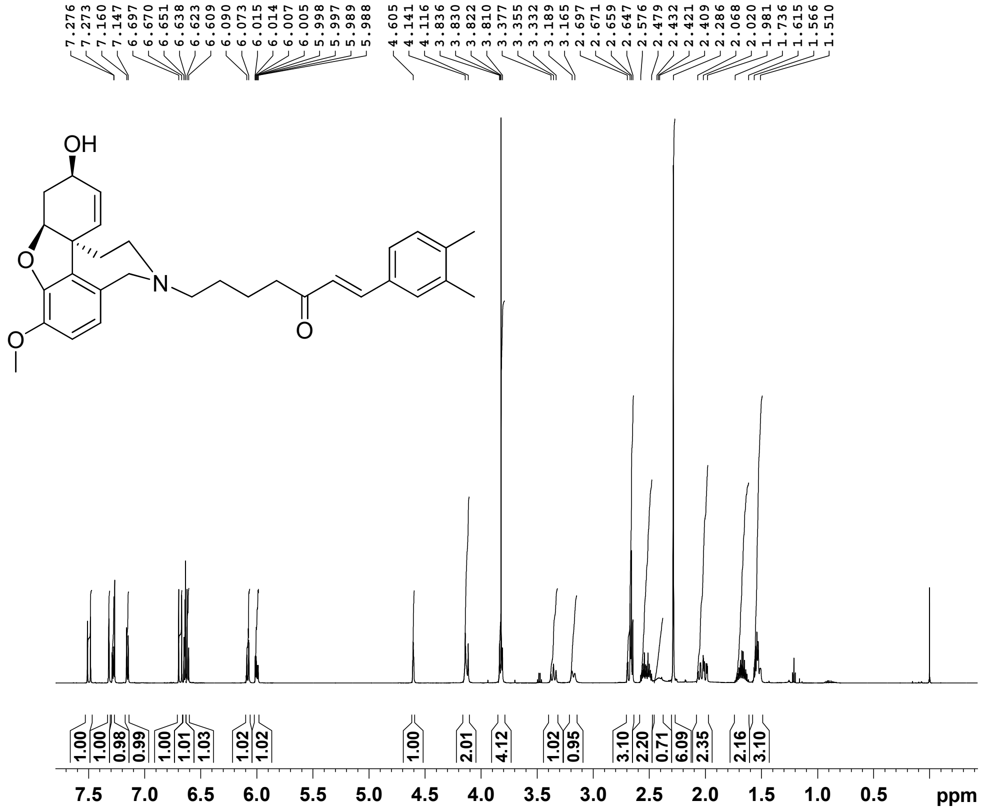

NAME	FK07602
EXPNO	11
PROCNO	1
Date_	20191105
Time	23.26 h
INSTRUM	spect
PROBHD	Z847801_0047 (
PULPROG	zg30
TD	32768
SOLVENT	CDCl3
NS	32
DS	0
SWH	9615.385 Hz
FIDRES	0.586877 Hz
AQ	1.7039860 sec
RG	114
DW	52.000 usec
DE	13.95 usec
TE	293.0 K
D1	1.00000000 sec
TD0	1
SFO1	600.0145608 MHz
NUC1	1H
P1	10.85 usec
SI	65536
SF	600.0100105 MHz
WDW	EM
SSB	0
LB	0.00 Hz
GB	0
PC	1.00

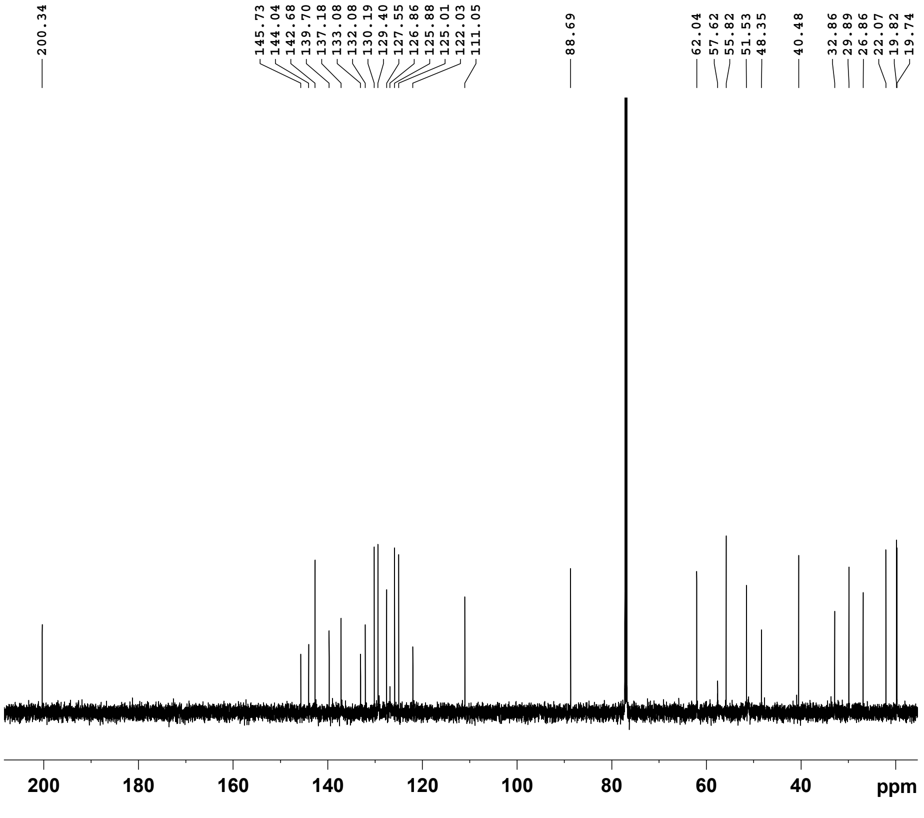

NAME	FK07602
EXPNO	16
PROCNO	1
Date_	20191105
Time	23.49 h
INSTRUM	spect
PROBHD	Z847801_0047 (
PULPROG	zgdc30
TD	32768
SOLVENT	CDCl3
NS	128
DS	0
SWH	36057.691 Hz
FIDRES	2.200787 Hz
AQ	0.4544329 sec
RG	2050
DW	13.867 usec
DE	6.50 usec
TE	293.0 K
D1	1.50000000 sec
D11	0.03000000 sec
TD0	1
SFO1	150.8892338 MHz
NUC1	13C
P1	9.80 usec
SI	65536
SF	150.8726442 MHz
WDW	EM
SSB	0
LB	1.00 Hz
GB	0
PC	1.40

Compound 6a:

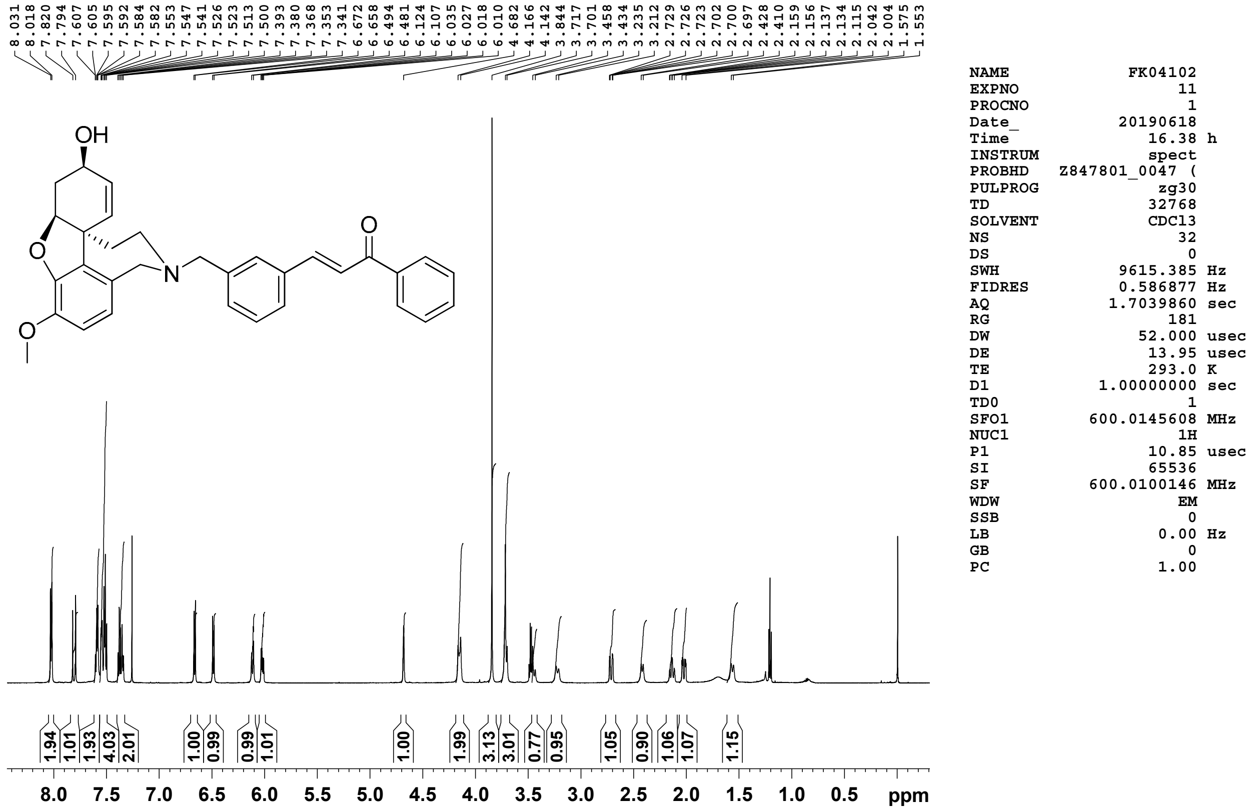
8.031
8.018
7.820
7.794
7.607
7.605
7.595
7.592
7.584
7.582
7.553
7.547
7.541
7.526
7.523
7.513
7.500
7.393
7.380
7.368
7.353
7.341
6.672
6.658
6.494
6.481
6.124
6.107
6.035
6.027
6.018
6.010
4.682
4.166
4.142
3.844
3.717
3.701
3.458
3.434
3.235
3.212
2.729
2.726
2.723
2.702
2.700
2.697
2.428
2.410
2.159
2.156
2.137
2.134
2.115
2.042
2.004
1.575
1.553
OH
O
N
O
1.94
1.01
1.93
4.03
2.01
1.00
0.99
0.99
1.01
1.00
1.99
3.13
3.01
0.77
0.95
1.05
0.90
1.06
1.07
1.15
8.0 7.5 7.0 6.5 6.0 5.5 5.0 4.5 4.0 3.5 3.0 2.5 2.0 1.5 1.0 0.5 ppm
NAME FK04102
EXPNO 11
PROCNO 1
Date_ 20190618
Time 16.38 h
INSTRUM spect
PROBHD Z847801_0047 (
PULPROG zg30
TD 32768
SOLVENT CDCl3
NS 32
DS 0
SWH 9615.385 Hz
FIDRES 0.586877 Hz
AQ 1.7039860 sec
RG 181
DW 52.000 usec
DE 13.95 usec
TE 293.0 K
D1 1.00000000 sec
TD0 1
SFO1 600.0145608 MHz
NUC1 1H
P1 10.85 usec
SI 65536
SF 600.0100146 MHz
WDW EM
SSB 0
LB 0.00 Hz
GB 0
PC 1.00

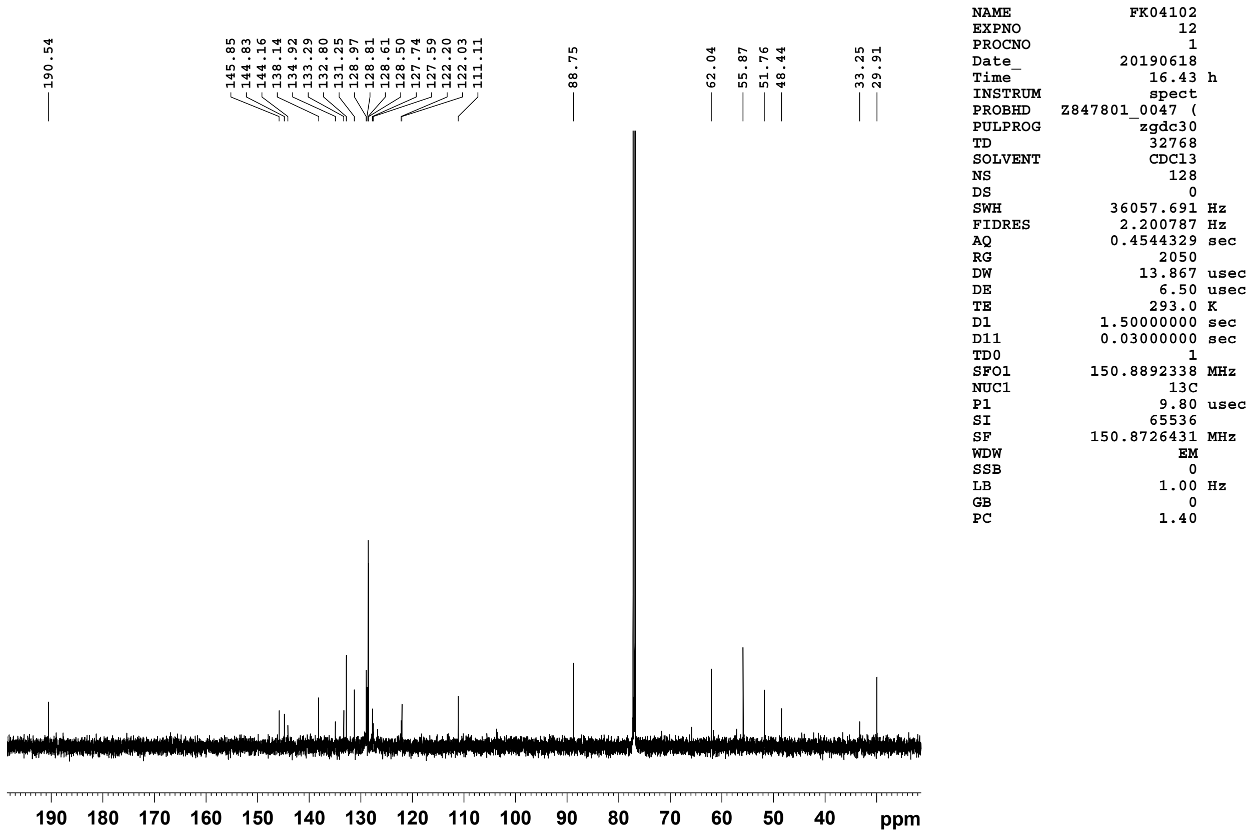
190.54
145.85
144.83
144.16
138.14
134.92
133.29
132.80
131.25
128.97
128.81
128.61
128.50
127.74
127.59
122.20
122.03
111.11
88.75
62.04
55.87
51.76
48.44
33.25
29.91
190 180 170 160 150 140 130 120 110 100 90 80 70 60 50 40 ppm
NAME FK04102
EXPNO 12
PROCNO 1
Date_ 20190618
Time 16.43 h
INSTRUM spect
PROBHD Z847801_0047 (
PULPROG zgdc30
TD 32768
SOLVENT CDCl3
NS 128
DS 0
SWH 36057.691 Hz
FIDRES 2.200787 Hz
AQ 0.4544329 sec
RG 2050
DW 13.867 usec
DE 6.50 usec
TE 293.0 K
D1 1.50000000 sec
D11 0.03000000 sec
TD0 1
SFO1 150.8892338 MHz
NUC1 13C
P1 9.80 usec
SI 65536
SF 150.8726431 MHz
WDW EM
SSB 0
LB 1.00 Hz
GB 0
PC 1.40

Compound 6b:

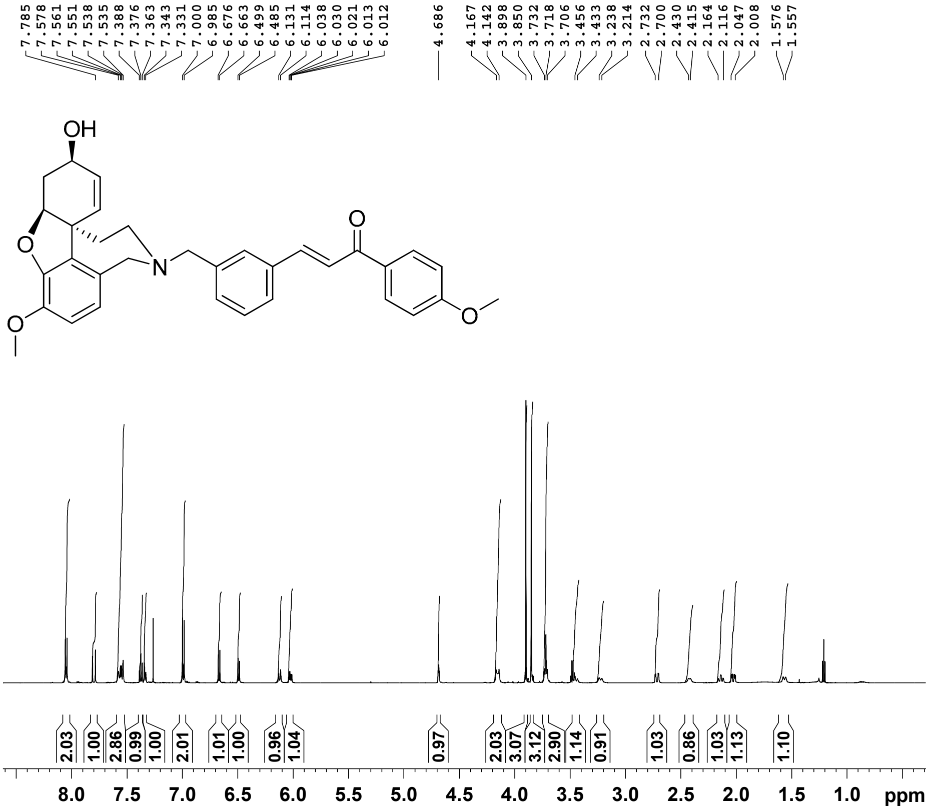

NAME	GC1252	
EXPNO	11	
PROCNO	1	
Date_	20200218	
Time	17.33	h
INSTRUM	spect	
PROBHD	Z847801_0047 (	
PULPROG	zg30	
TD	32768	
SOLVENT	CDCl3	
NS	16	
DS	0	
SWH	9615.385	Hz
FIDRES	0.586877	Hz
AQ	1.7039860	sec
RG	144	
DW	52.000	usec
DE	13.95	usec
TE	293.0	K
D1	1.00000000	sec
TD0	1	
SFO1	600.0145608	MHz
NUC1	1H	
P1	10.85	usec
SI	65536	
SF	600.0100112	MHz
WDW	EM	
SSB	0	
LB	0.00	Hz
GB	0	
PC	1.00	

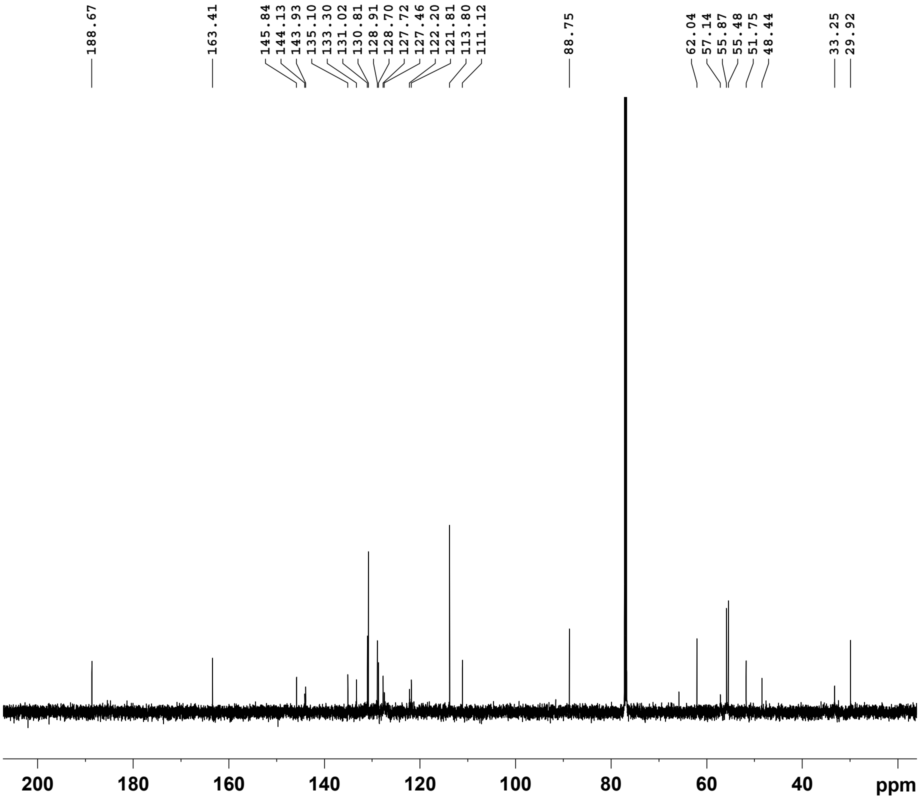

NAME	GC1252	
EXPNO	12	
PROCNO	1	
Date_	20200218	
Time	17.38	h
INSTRUM	spect	
PROBHD	Z847801_0047 (	
PULPROG	zgdc30	
TD	32768	
SOLVENT	CDCl3	
NS	128	
DS	0	
SWH	36057.691	Hz
FIDRES	2.200787	Hz
AQ	0.4544329	sec
RG	2050	
DW	13.867	usec
DE	6.50	usec
TE	293.1	K
D1	1.50000000	sec
D11	0.03000000	sec
TD0	1	
SFO1	150.8892338	MHz
NUC1	13C	
P1	9.80	usec
SI	65536	
SF	150.8726435	MHz
WDW	EM	
SSB	0	
LB	1.00	Hz
GB	0	
PC	1.40	

Compound 8b:

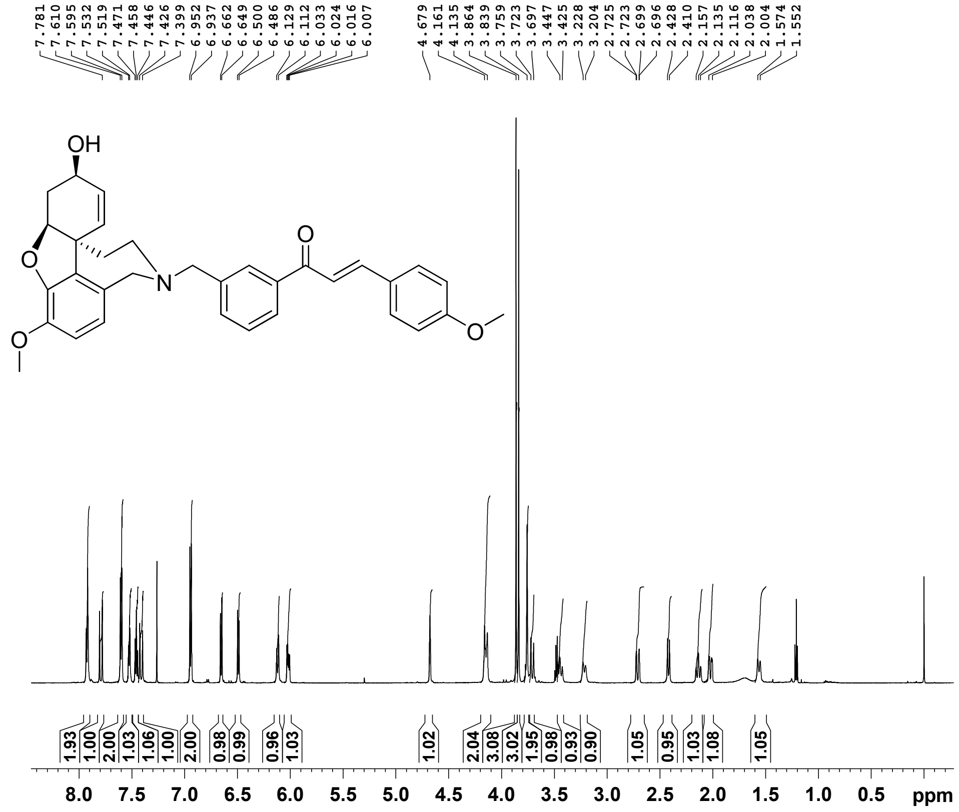

NAME	FL02102
EXPNO	11
PROCNO	1
Date_	20200130
Time	18.52 h
INSTRUM	spect
PROBHD	Z847801_0047 (
PULPROG	zg30
TD	32768
SOLVENT	CDCl3
NS	32
DS	0
SWH	9615.385 Hz
FIDRES	0.586877 Hz
AQ	1.7039860 sec
RG	161
DW	52.000 usec
DE	13.95 usec
TE	293.0 K
D1	1.00000000 sec
TD0	1
SFO1	600.0145608 MHz
NUC1	1H
P1	10.85 usec
SI	65536
SF	600.0100119 MHz
WDW	EM
SSB	0
LB	0.00 Hz
GB	0
PC	1.00

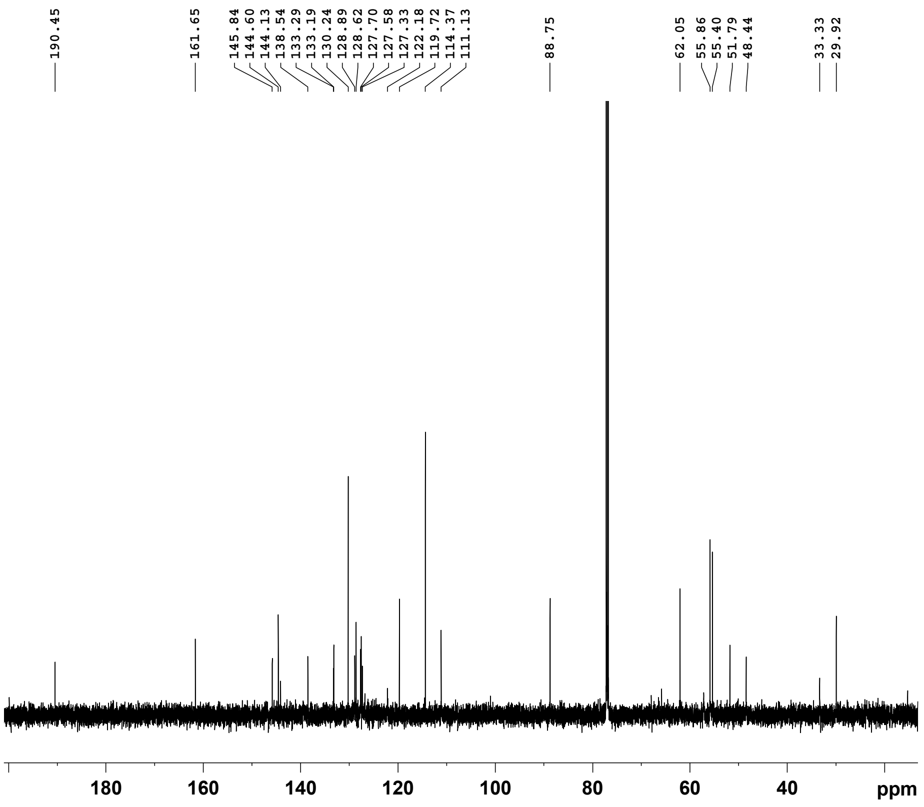

NAME	FL02102
EXPNO	12
PROCNO	1
Date_	20200130
Time	18.57 h
INSTRUM	spect
PROBHD	Z847801_0047 (
PULPROG	zgdc30
TD	32768
SOLVENT	CDCl3
NS	128
DS	0
SWH	36057.691 Hz
FIDRES	2.200787 Hz
AQ	0.4544329 sec
RG	2050
DW	13.867 usec
DE	6.50 usec
TE	293.1 K
D1	1.50000000 sec
D11	0.03000000 sec
TD0	1
SFO1	150.8892338 MHz
NUC1	13C
P1	9.80 usec
SI	65536
SF	150.8726432 MHz
WDW	EM
SSB	0
LB	1.00 Hz
GB	0
PC	1.40

Compound 8c:

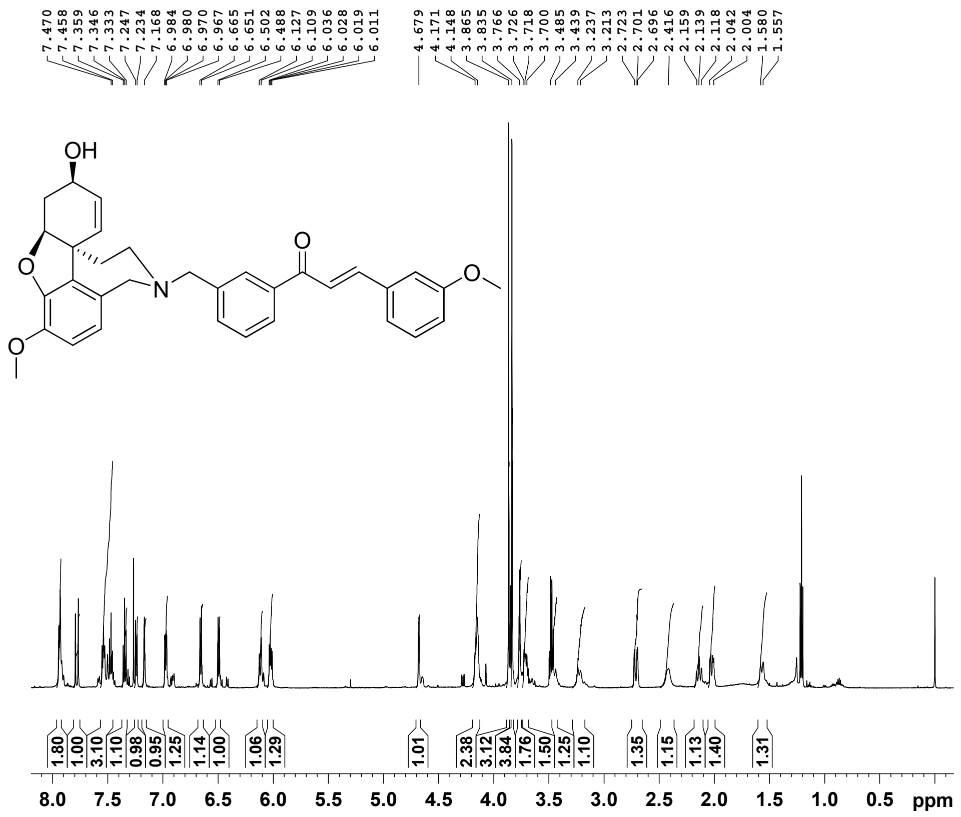

NAME	GC1382	
EXPNO	11	
PROCNO	1	
Date_	20200130	
Time	20.53	h
INSTRUM	spect	
PROBHD	Z847801_0047 (	
PULPROG	zg30	
TD	32768	
SOLVENT	CDCl3	
NS	32	
DS	0	
SWH	9615.385	Hz
FIDRES	0.586877	Hz
AQ	1.7039860	sec
RG	144	
DW	52.000	usec
DE	13.95	usec
TE	293.0	K
D1	1.00000000	sec
TD0	1	
SFO1	600.0145608	MHz
NUC1	1H	
P1	10.85	usec
SI	65536	
SF	600.0100113	MHz
WDW	EM	
SSB	0	
LB	0.00	Hz
GB	0	
PC	1.00	

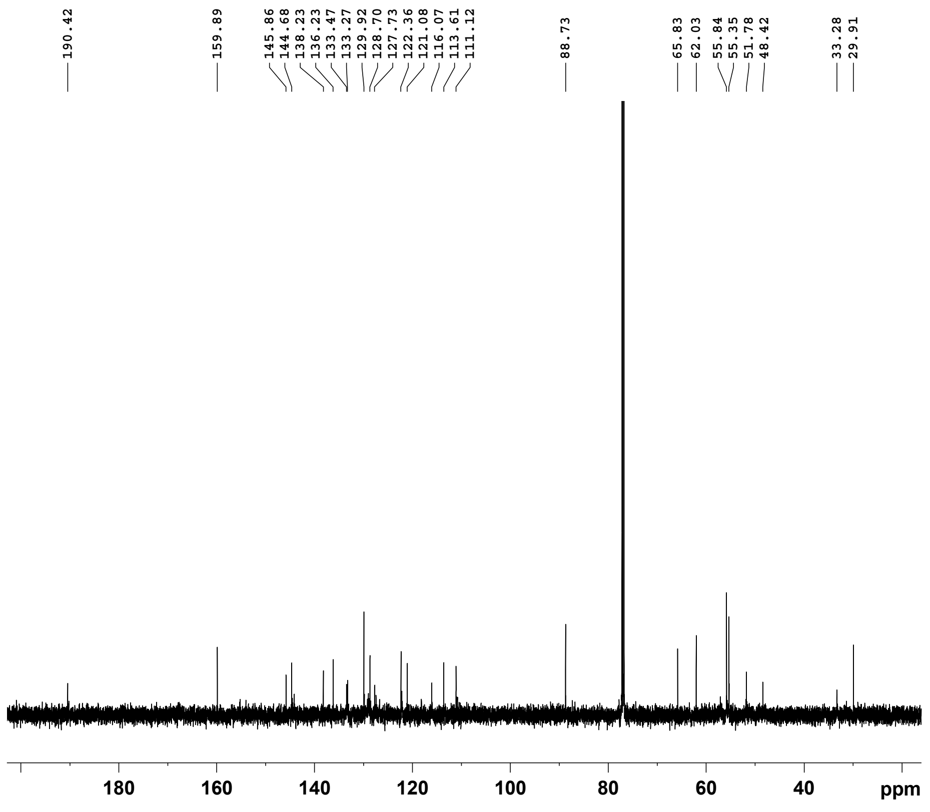

NAME	GC1382	
EXPNO	12	
PROCNO	1	
Date_	20200130	
Time	20.58	h
INSTRUM	spect	
PROBHD	Z847801_0047 (	
PULPROG	zgdc30	
TD	32768	
SOLVENT	CDCl3	
NS	128	
DS	0	
SWH	36057.691	Hz
FIDRES	2.200787	Hz
AQ	0.4544329	sec
RG	2050	
DW	13.867	usec
DE	6.50	usec
TE	293.1	K
D1	1.50000000	sec
D11	0.03000000	sec
TD0	1	
SFO1	150.8892338	MHz
NUC1	13C	
P1	9.80	usec
SI	65536	
SF	150.8726435	MHz
WDW	EM	
SSB	0	
LB	1.00	Hz
GB	0	
PC	1.40	

Compound 8f:

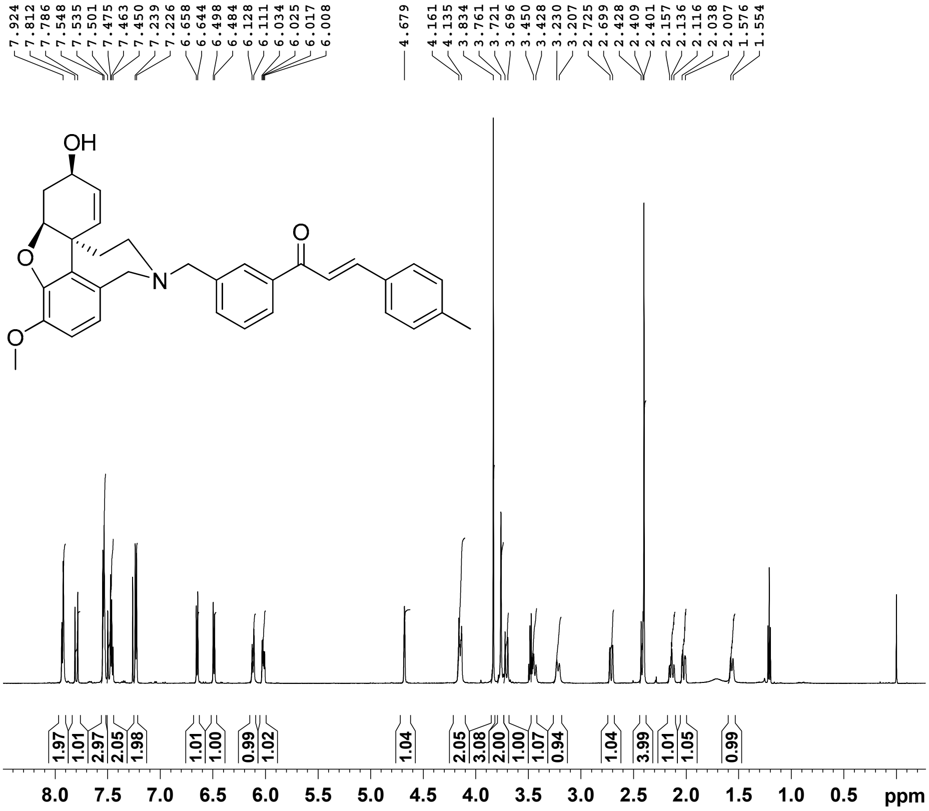

NAME	FL02302
EXPNO	11
PROCNO	1
Date_	20200130
Time	19.22 h
INSTRUM	spect
PROBHD	Z847801_0047 (
PULPROG	zg30
TD	32768
SOLVENT	CDCl3
NS	32
DS	0
SWH	9615.385 Hz
FIDRES	0.586877 Hz
AQ	1.7039860 sec
RG	144
DW	52.000 usec
DE	13.95 usec
TE	293.0 K
D1	1.00000000 sec
TD0	1
SFO1	600.0145608 MHz
NUC1	1H
P1	10.85 usec
SI	65536
SF	600.0100126 MHz
WDW	EM
SSB	0
LB	0.00 Hz
GB	0
PC	1.00

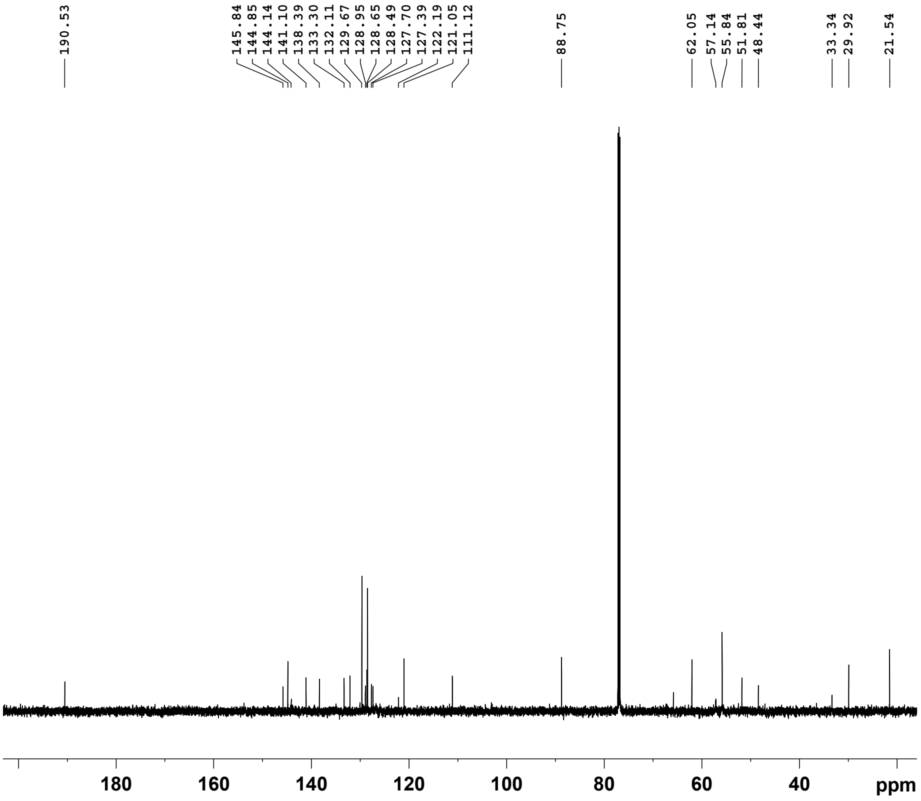

NAME	FL02302
EXPNO	12
PROCNO	1
Date_	20200130
Time	19.27 h
INSTRUM	spect
PROBHD	Z847801_0047 (
PULPROG	zgdc30
TD	32768
SOLVENT	CDCl3
NS	128
DS	0
SWH	36057.691 Hz
FIDRES	2.200787 Hz
AQ	0.4544329 sec
RG	2050
DW	13.867 usec
DE	6.50 usec
TE	293.0 K
D1	1.50000000 sec
D11	0.03000000 sec
TD0	1
SFO1	150.8892338 MHz
NUC1	13C
P1	9.80 usec
SI	65536
SF	150.8726435 MHz
WDW	EM
SSB	0
LB	1.00 Hz
GB	0
PC	1.40

Compound 8g:

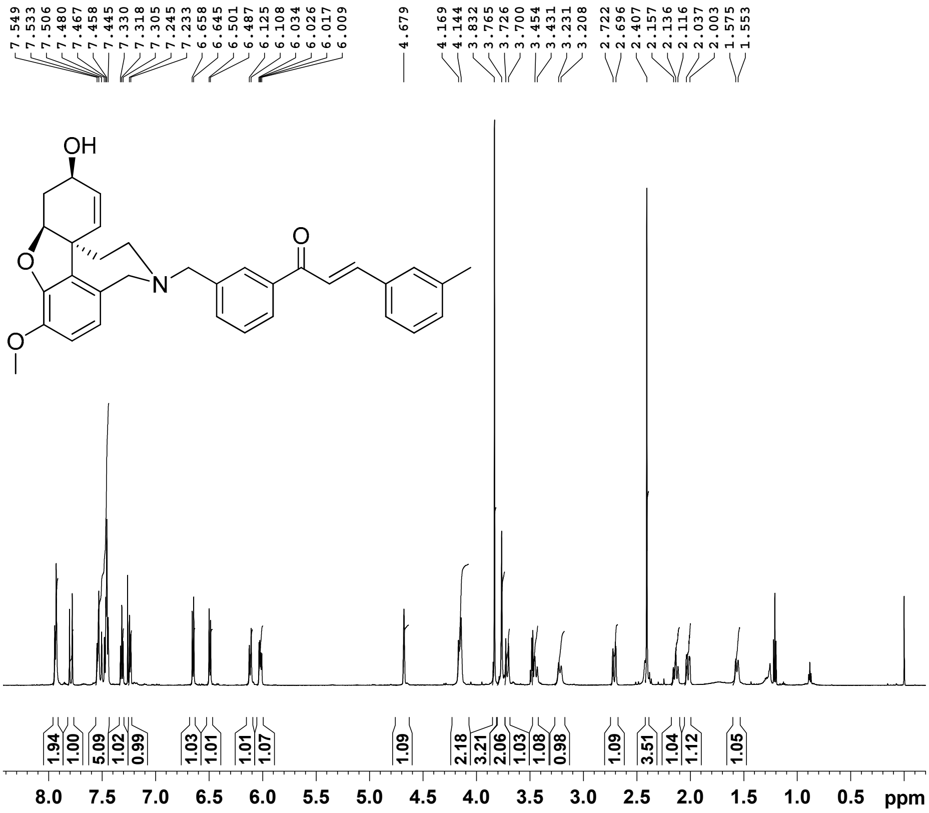

NAME	GC1362
EXPNO	11
PROCNO	1
Date_	20200130
Time	20.22 h
INSTRUM	spect
PROBHD	Z847801_0047 (
PULPROG	zg30
TD	32768
SOLVENT	CDCl3
NS	32
DS	0
SWH	9615.385 Hz
FIDRES	0.586877 Hz
AQ	1.7039860 sec
RG	144
DW	52.000 usec
DE	13.95 usec
TE	293.0 K
D1	1.00000000 sec
TD0	1
SFO1	600.0145608 MHz
NUC1	1H
P1	10.85 usec
SI	65536
SF	600.0100128 MHz
WDW	EM
SSB	0
LB	0.00 Hz
GB	0
PC	1.00

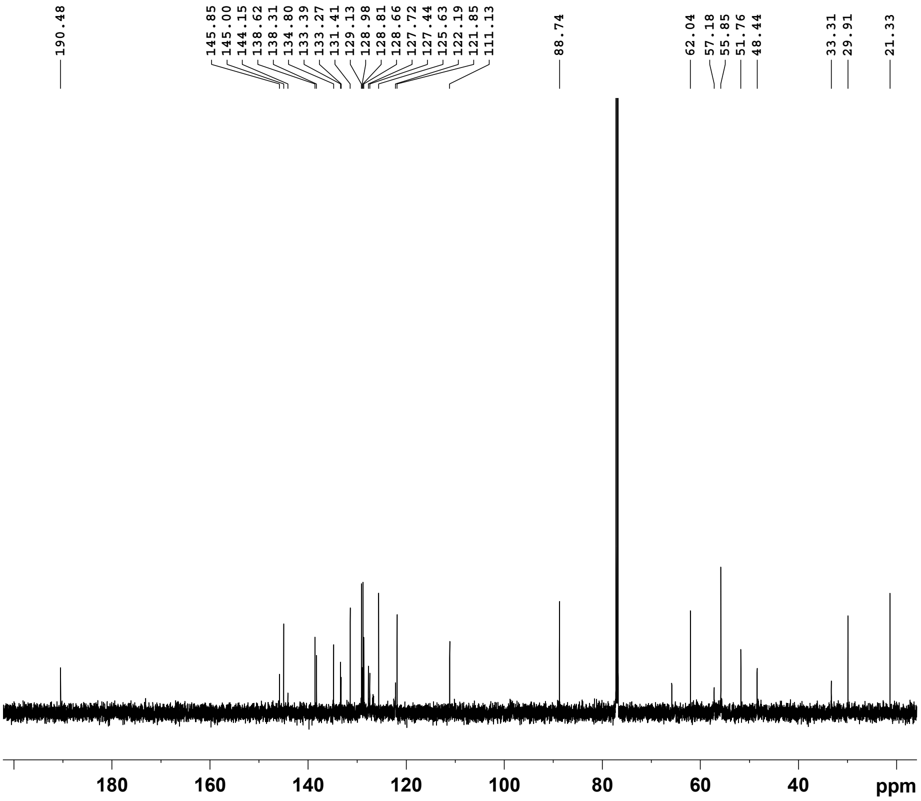

NAME	GC1362
EXPNO	12
PROCNO	1
Date_	20200130
Time	20.27 h
INSTRUM	spect
PROBHD	Z847801_0047 (
PULPROG	zgdc30
TD	32768
SOLVENT	CDCl3
NS	128
DS	0
SWH	36057.691 Hz
FIDRES	2.200787 Hz
AQ	0.4544329 sec
RG	2050
DW	13.867 usec
DE	6.50 usec
TE	293.0 K
D1	1.50000000 sec
D11	0.03000000 sec
TD0	1
SFO1	150.8892338 MHz
NUC1	13C
P1	9.80 usec
SI	65536
SF	150.8726434 MHz
WDW	EM
SSB	0
LB	1.00 Hz
GB	0
PC	1.40

Compound 8h:

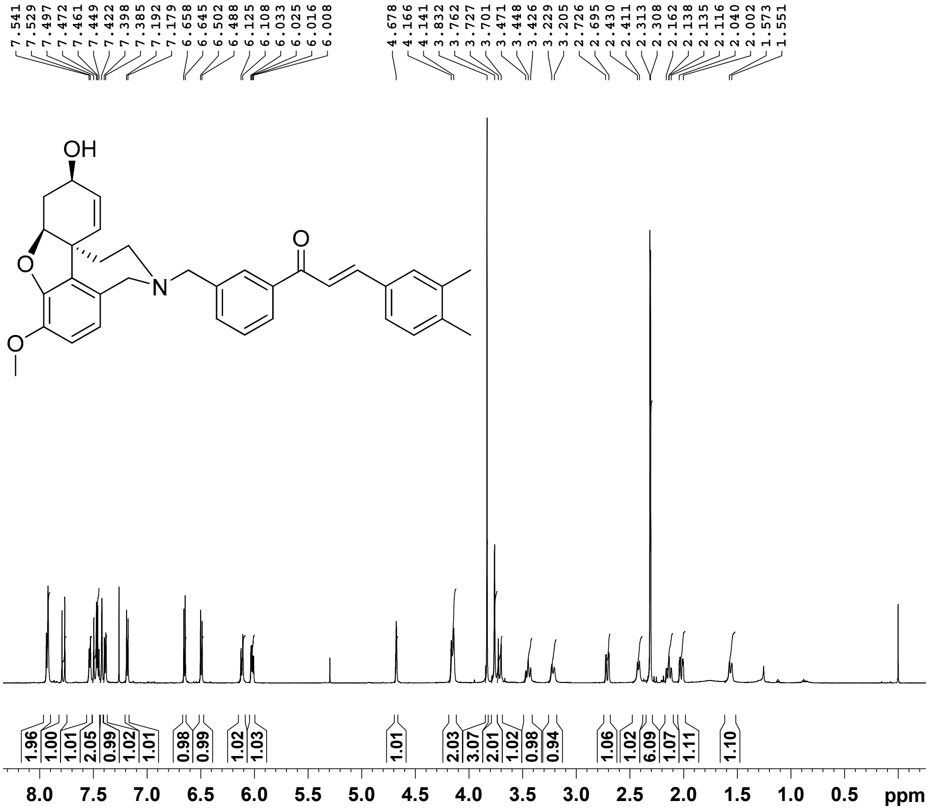

NAME	FL02502	
EXPNO	11	
PROCNO	1	
Date_	20200130	
Time	19.53	h
INSTRUM	spect	
PROBHD	Z847801_0047 (	
PULPROG	zg30	
TD	32768	
SOLVENT	CDCl3	
NS	32	
DS	0	
SWH	9615.385	Hz
FIDRES	0.586877	Hz
AQ	1.7039860	sec
RG	128	
DW	52.000	usec
DE	13.95	usec
TE	293.0	K
D1	1.00000000	sec
TD0	1	
SFO1	600.0145608	MHz
NUC1	1H	
P1	10.85	usec
SI	65536	
SF	600.0100130	MHz
WDW	EM	
SSB	0	
LB	0.00	Hz
GB	0	
PC	1.00	

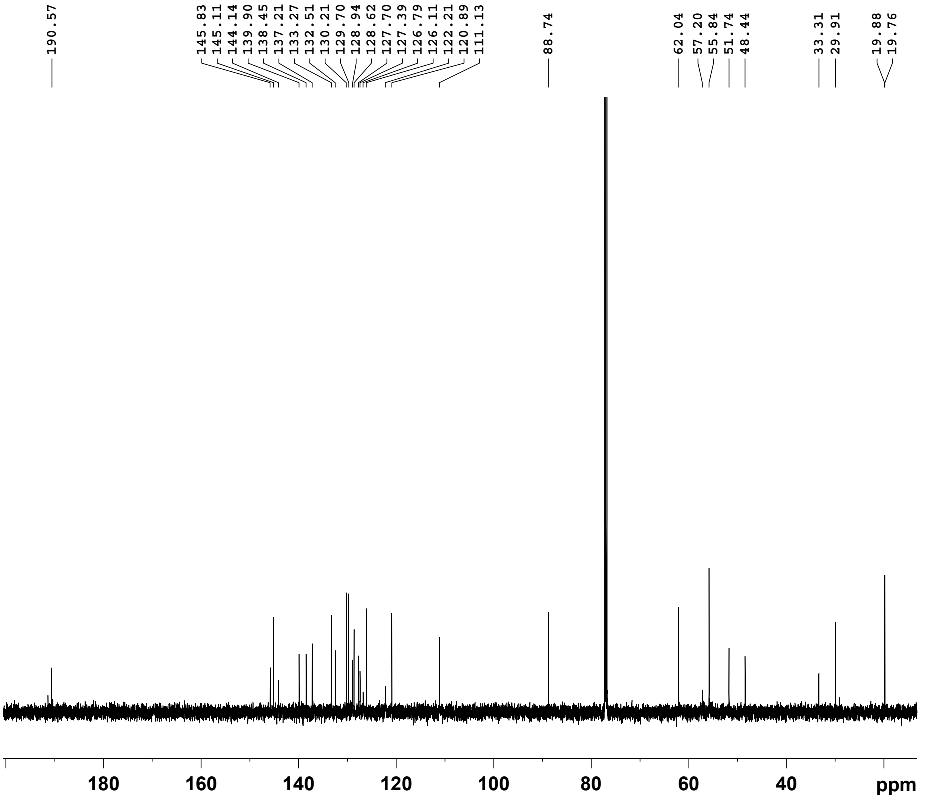

NAME	FL02502	
EXPNO	12	
PROCNO	1	
Date_	20200130	
Time	19.58	h
INSTRUM	spect	
PROBHD	Z847801_0047 (	
PULPROG	zgdc30	
TD	32768	
SOLVENT	CDCl3	
NS	128	
DS	0	
SWH	36057.691	Hz
FIDRES	2.200787	Hz
AQ	0.4544329	sec
RG	2050	
DW	13.867	usec
DE	6.50	usec
TE	293.1	K
D1	1.50000000	sec
D11	0.03000000	sec
TD0	1	
SFO1	150.8892338	MHz
NUC1	13C	
P1	9.80	usec
SI	65536	
SF	150.8726441	MHz
WDW	EM	
SSB	0	
LB	1.00	Hz
GB	0	
PC	1.40	
